# An Integrated Approach toward NanoBRET Tracers for Analysis of GPCR Ligand Engagement

**DOI:** 10.3390/molecules26102857

**Published:** 2021-05-12

**Authors:** Michael P. Killoran, Sergiy Levin, Michelle E. Boursier, Kristopher Zimmerman, Robin Hurst, Mary P. Hall, Thomas Machleidt, Thomas A. Kirkland, Rachel Friedman Ohana

**Affiliations:** 1 Promega Corporation, 2800 Woods Hollow, Fitchburg, WI 53711, USA; mike.killoran@promega.com (M.P.K.); michelle.boursier@gmail.com (M.E.B.); Kris.Zimmerman@promega.com (K.Z.); robin.hurst@promega.com (R.H.); Mary.Hall@promega.com (M.P.H.); Thomas.Machleidt@promega.com (T.M.); 2 Promega Biosciences LLC, 277 Granada Drive, San Luis Obispo, CA 93401, USA; serge.levin@gmail.com (S.L.); thomas.kirkland@promega.com (T.A.K.)

**Keywords:** GPCRS, ligand-engagement, NanoBRET, HiBiT, in silico screen

## Abstract

Gaining insight into the pharmacology of ligand engagement with G-protein coupled receptors (GPCRs) under biologically relevant conditions is vital to both drug discovery and basic research. NanoLuc-based bioluminescence resonance energy transfer (NanoBRET) monitoring competitive binding between fluorescent tracers and unmodified test compounds has emerged as a robust and sensitive method to quantify ligand engagement with specific GPCRs genetically fused to NanoLuc luciferase or the luminogenic HiBiT peptide. However, development of fluorescent tracers is often challenging and remains the principal bottleneck for this approach. One way to alleviate the burden of developing a specific tracer for each receptor is using promiscuous tracers, which is made possible by the intrinsic specificity of BRET. Here, we devised an integrated tracer discovery workflow that couples machine learning-guided in silico screening for scaffolds displaying promiscuous binding to GPCRs with a blend of synthetic strategies to rapidly generate multiple tracer candidates. Subsequently, these candidates were evaluated for binding in a NanoBRET ligand-engagement screen across a library of HiBiT-tagged GPCRs. Employing this workflow, we generated several promiscuous fluorescent tracers that can effectively engage multiple GPCRs, demonstrating the efficiency of this approach. We believe that this workflow has the potential to accelerate discovery of NanoBRET fluorescent tracers for GPCRs and other target classes.

## 1. Introduction

G protein-coupled receptors (GPCRs) are among the most widely studied pharmacological targets [1,2]. They are substantially involved in regulation of multiple physiological processes and their malfunction has been implicated in numerous diseases [1,2]. Consequentially, detailed understanding of their engagement with bioactive compounds, including binding affinity and kinetics, is imperative to both drug discovery and basic research. The realization that the cellular environment can influence these measurements has prompted the development of cell-based ligand-engagement assays as an alternative to traditional biochemical radioligand binding. These assays exploit the inherent distance constraints of resonance energy transfer to detect molecular proximity between GPCRs labeled with an energy donor (i.e., fluorescent protein, chromophore, or luciferase) and their ligands labeled with an energy acceptor fluorophore (fluorescent tracers) [3,4]. Among these assays, NanoLuc luciferase-based bioluminescence resonance energy transfer (NanoBRET) has been reported to be sensitive, robust, and capable of real-time measurements [5,6,7,8]. The bright luminescence generated by NanoLuc-based energy donors (i.e., full length NanoLuc or HiBiT complemented with LgBiT, a complementation system derived from NanoLuc) [9,10] enabled GPCRs ligand-engagement measurements previously impossible with other luciferases [5,6,7,11]. The HiBiT/LgBiT high affinity complementation reporter has been especially useful for GPCRs studies [5,12]. This is predominantly due to the cell impermeability of LgBiT, which limits signal from complementation with HiBiT-tagged GPCRs to the cell surface, eliminating intracellular background luminescence and resulting in assays with overall greater sensitivity, dynamic range, and robustness [5].

Resonance energy transfer ligand-engagement assays offer specific and sensitive measurements of molecular proximity between fluorescent tracers and their cognate GPCRs genetically fused to an energy donor. Typically, engagement characteristics for unmodified ligands are revealed through competitive displacement of a bound fluorescent tracer. However, rapid and efficient generation of receptor specific tracers is often challenging. Conjugation of a fluorophore to a bioactive compound introduces a significant modification to the chemical structure, frequently disrupting critical binding interactions and altering physiochemical properties [13,14]. Therefore, judicious choice of fluorophore, linker chemistry, and conjugation site are all important for preserving the relevant properties of a parental compound. Optimizing each of these aspects can be a labor-intensive process. 

The exquisite specificity of resonance energy transfer ligand-engagement assays governed by binding to targets tethered to an energy donor offers an opportunity to utilize promiscuous fluorescent tracers [15,16]. Such tracers can enable ligand-engagement assays for multiple targets, thereby reducing the requirement of developing a selective tracer for each target. However, unlike other target classes such as kinases [16], GPCR families share little structural homology in their ligand binding pockets or cross reactivity among their natural ligands [1,2,17]. Therefore, identification of compounds with a promiscuous GPCR binding profile that are suitable for development into fluorescent tracers poses a significant challenge. Taken together, there is a need for a creative approach to accelerate the identification, modification, and evaluation of fluorescent tracer candidates for GPCR ligand-engagement assays.

Ligand-based in silico screening can help to address part of this challenge by providing a means to rapidly search a vast chemical space for bioactive compounds with a promiscuous binding profile [17,18,19,20]. When designed to exploit the chemical information of known ligands, it can identify patterns associated with receptor interactions in an automated fashion without any knowledge of a receptor’s 3D structure [17,19]. Machine learning algorithms, are particularly well suited for pattern recognition and generalization tasks. When trained to identify a shared set of chemical features among compounds known to bind a specific target or target family, they can use those chemical signatures to identify other compounds likely to interact with the targets. This approach can guide an automated, preliminary selection of promiscuous compounds for highly diverse and selective target classes like GPCRs, for which manual searches would be very challenging.

Compound modification with a fluorophore provides an additional opportunity to improve the efficiency of tracer development. As mentioned above, the attachment of a fluorophore to a bioactive compound requires careful selection of a conjugation site. Often, the optimal site cannot be rationalized via structure activity relationships (SAR) and/or in silico docking analysis, requiring empirical evaluation of several conjugation sites. In addition, synthetic accessibility to such sites through de novo total synthesis is often complex and can be labor and time intensive. Recently, late-stage modification of organic compounds has emerged as a powerful approach for rapid exploration of chemical space [21,22,23]. Compared to de novo synthesis, this approach often results in significant time and cost savings by allowing modification of commercially available compounds. However, the structural complexity of many bioactive compounds remains a significant challenge for a site selective late-stage modification. Still, the toolbox of late-stage functionalization chemistries continues to expand, providing additional tools for rapid modification of an ever-growing list of chemical moieties.

Here, we describe an integrated workflow utilizing the approaches outlined above to accelerate the generation and evaluation of fluorescent tracers for GPCRs. To demonstrate the efficiency of this workflow, we used a machine learning model to predict the binding profiles of 17 model scaffolds and a blend of synthetic strategies to conjugate these scaffolds to a fluorophore. The resulting panel of fluorescent tracer candidates was evaluated in a NanoBRET ligand-engagement screen across a library of 184 HiBiT-tagged GPCRs. The screen revealed a good overall correlation with model predictions and provided valuable feedback on tracers’ binding profile, suitable conjugation site, and ultimately potential to deliver robust ligand-engagement assays. Thus far, this strategy has resulted in several fluorescent tracers that can effectively engage multiple receptors from one or more GPCR families. We believe this workflow has the potential to accelerate tracer discovery and to broaden the target classes amenable for NanoBRET ligand-engagement analyses.

## 2. Results and Discussion

### 2.1. Design of a Machine Learning Model for Predicting Interactions between Ligands and Their GPCR Target Families

The high specificity afforded by NanoBRET allows utilization of promiscuous fluorescent tracers for selective measurements at specific HiBiT-GPCRs, reducing the need to develop a specific tracer for each target. Given the abundance of available information on GPCR-ligand interactions in public databases, we sought to design an in silico screening strategy to identify compounds exhibiting a propensity for promiscuous binding interactions. We were motivated to use only chemical structures of known GPCR ligands as input data for machine learning since this would provide access to the most comprehensive compound set available, including those targeting GPCRs lacking 3-D structural information. The resulting model could be used to screen compounds with a minimal amount of chemical information needed.

The intent of our model design was to recognize common structural patterns shared by ligands of specific GPCR families and then use that information to predict the target family interactions for other compounds in an automated fashion. We chose to categorize compounds based on their target GPCR families rather than individual receptors for two reasons. First, the primary application of the model is to provide guidance for identifying compounds that could interact promiscuously across a broad range of GPCRs rather than with specific receptors or GPCR families. Second, it simplifies the modeling task by providing greater differentiation between classes, under the assumption that ligands interacting with receptors from different families are more likely to exhibit larger differences, which in turn are easier to distinguish by the model. In practice, this approach has made in silico screening of large datasets more tractable. Promiscuity across different GPCR families is expected to be a rare property and by grouping compounds into larger GPCR-family target classes it was easier to identify those with a high probability for multi-target interactions.

Throughout model development, we identified several steps critical to the success of this strategy including aspects of data processing as well as methods of model training and validation. Briefly, we processed our chemical structure dataset by focusing on minimizing sources of redundancy and bias while maximizing the diversity of chemical information available for machine learning. Using chemical structures encoded as molecular fingerprints we trained the model to recognize patterns of shared molecular features among compounds that bind the same target class (GPCR family). The capacity of the model to use those signature patterns to classify compounds into their GPCR target families was validated using a test set comprising 30% of the compounds, which were withheld during training.

### 2.2. Data Acquisition and Preparation for Machine Learning

Training a machine learning model to recognize patterns of molecular interactions with different GPCR families requires a large, representative dataset comprising similarly diverse ligands for each family. Among available data sources, we chose the GPCR ligand-associated (GLASS) database as it provided an easily accessible and comprehensive dataset of known GPCR ligands [24]. However, since this approach requires only chemical structures and their known GPCR target families as input, other data sources such as results from high-throughput screenings or alternative databases could be used instead or in supplement to the information used in this study.

**Figure 1:**
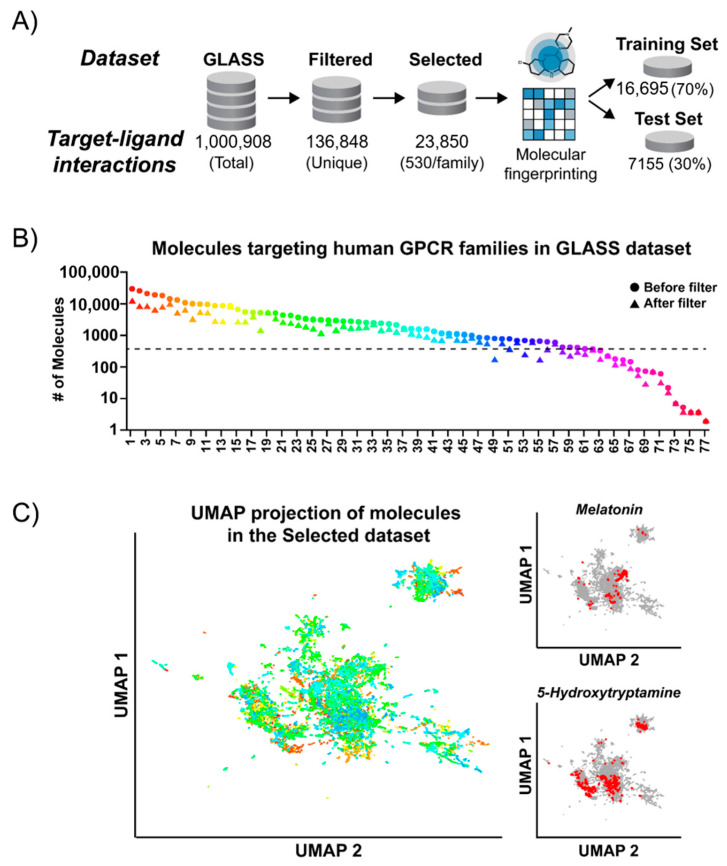
Processing and analysis of GPCR-ligand interaction data. (**A**) Data processing scheme for preparing the training and test datasets for machine learning. Compounds from the GLASS database with known CHEMBL IDs were filtered for unique interactions with human GPCRs followed by removal of redundant structures and random selection of 530 unique interactions for each GPCR family. Compounds in the Selected dataset were encoded as molecular fingerprints and split into training and test datasets. (**B**) The number of compounds interacting with each GPCR family in the GLASS dataset before and after processing. GPCR family names are shown in Supplementary Materials Table S1. (**C**) UMAP projection of compounds in the Selected dataset highlights the spatial clustering of unique scaffolds used to train the machine learning model. For visualization, the UMAP algorithm embeds the multi-dimensional molecular fingerprints of the training set into a two-dimensional representation (UMAP 1 and UMAP 2) while preserving the essential topological structure of the data. Each compound is represented by a single point and colored according to its GPCR family interaction as in (**B**). Inset UMAP projections (right) show examples of the dense clustering observed for compounds interacting with specific GPCR families, highlighting the separation of family-specific ligands.

Data acquired from the GLASS database contained over a million ligand-receptor interactions for GPCRs from multiple species. Since we were primarily focused on developing NanoBRET tracers to interrogate ligand engagement of human GPCRs, we first filtered out all redundant molecular structures and subsequently selected for ligands known to target human receptors (Figure 1A). Comparison of the GLASS datasets before and after processing indicated that 45 GPCR families each retained over 500 unique compounds (Figure 1B). Ideally, the subsets used to train and evaluate the machine learning model would uniformly represent the diversity of ligands associated with each GPCR target family. Accordingly, to eliminate family imbalances and retain most of the starting chemical diversity, we assembled a “Selected dataset” comprising an equal number of 530 random compounds targeting each of the remaining GPCR families. We used 70% of these compounds (i.e., 371 compounds per family) as a training set while the remaining 30%, which were withheld during training, were used as a test set. During the development of the model, we explored a range of training set sizes and found that these selection criteria provided the best predictive classification for compounds in our test set (Supplementary Materials Figure S1).

The 23,850 compounds in our Selected dataset were converted into a vector format, which can serve as input for machine learning by encoding chemical structures as molecular fingerprints. Chemical information encoded as molecular fingerprints has been successfully used in machine learning tasks [25] and can include many formats ranging from the presence of substructures to descriptors of topological states or electronic environments [26,27,28]. Throughout the development of our final machine learning model, we systematically evaluated over a dozen different models utilizing single or multiple molecular fingerprinting schemes for their capacity to classify compounds in our test set (Supplementary Materials Figure S2). For each compound, the GPCR family with the highest classification probability was compared to the known target family as annotated in the GLASS database. We found that models based on fingerprint schemes exhibiting high overall accuracy (i.e., the true positive and negative predictions) but having low correlation among their true positive predictions performed best in combination, synergistically improving classification accuracy for compounds in our test set. However, improvements in test set accuracy did not always translate to higher correlation between model predictions and empirical NanoBRET screening data across all the tested GPCR families. In the end, our focus on identifying scaffolds exhibiting promiscuous binding properties led us to select the model with the highest accuracy on empirical NanoBRET data rather than on the test set. Overall, we found that the effectiveness of training the machine learning model was highly dependent on the structure of the training dataset, choice of molecular fingerprint representations, and the classification task the model is applied to.

### 2.3. Analysis of the Machine Learning Dataset Using UMAP

Given the dependence of our model’s performance on the structure of the training set, we sought to further investigate the structural relationships among compounds used for machine learning. Using the chemical information encoded by molecular fingerprints, we analyzed the underlying structure of the Selected dataset via Uniform Manifold Approximation and Projection (UMAP), an unsupervised learning algorithm [29]. UMAP reduces the high dimensional chemical information of fingerprints into lower dimensions while preserving the global structure of the dataset, enabling visualization and identification of latent patterns. Representation of the Selected dataset in this manner, which can be used as a measure of global chemical similarity, resulted in several large, independent clusters (Figure 1C). In addition, many small but dense single-color areas, which represent highly similar compounds that target specific GPCR families were noticeable. This latter type of pattern would be expected in data from large repositories that aggregate results for many target-specific scaffolds and closely related analogs.

Comparing the clustering patterns in UMAP space for individual GPCR families with their model’s performance metrics provided additional insights into the influence of data structure on model fitting. For example, compounds associated with GPCR families for which the model delivered classifications with high accuracy and sensitivity, such as the Melatonin receptor family, also displayed tight clustering in UMAP space (Figure 1C, top right). This suggested these compounds share a greater number of common structural features, which could be more easily associated with their target family during model training. In contrast, compounds associated with GPCR families for which the model delivered classifications with lower sensitivity such as 5-Hydroxytryptamine receptors, displayed a larger spread in UMAP space. This likely reflects fewer common patterns among the chemical features of compounds associated with these families, which makes fitting with a model more difficult (Figure 1C, bottom right).

One of the limitations using data acquired from large databases in this way is the difficulty of confirming information aggregated from multiple sources. Accordingly, several explanations are possible for the lower accuracy and misclassification of the model for some individual GPCR families, including low affinity interactions or assay-specific results. Still, the observed family-specific clustering in UMAP space for the majority of compounds indicates that our training set encoded sufficient chemical information to separate these high dimensional patterns when classifying compounds by their target GPCR family.

### 2.4. Development of a Machine Learning Model Classifying GPCR-Ligand Interactions

To provide a functional association between ligands and their known GPCR target families, we trained a random forest model to recognize relevant patterns of molecular fingerprints in our training set and to classify them by their annotated GPCR target families in the GLASS database. The simultaneous use of many uncorrelated decision trees by random forest algorithms provides several advantages for modeling the type of data in our training set. For example, they perform well on large datasets with multiple features, are robust to outliers and redundancy, and excel at error minimization during multi-class predictions tasks [30]. After training the model, we used our test set to independently evaluate its performance. For the 7155 compounds across 45 different GPCR families we observed a total classification accuracy of 80.9%. Overall, this result indicated that the machine learning model was able to successfully fit a function relating molecular fingerprints to specific GPCR target families and to make accurate predictions on a set of previously unseen compounds. As a control, we trained a model with randomized labels of ligand-GPCR family associations. We found that classification accuracy for the test set dropped significantly, down to 0.02%, the expected value for random classification across the 45 GPCR families (Supplementary Materials Figure S1). This result confirmed the model was specific in associating molecular fingerprint patterns with GPCR target families.

### 2.5. Evaluation of Model Predictions for Individual GPCR Families

**Figure 2:**
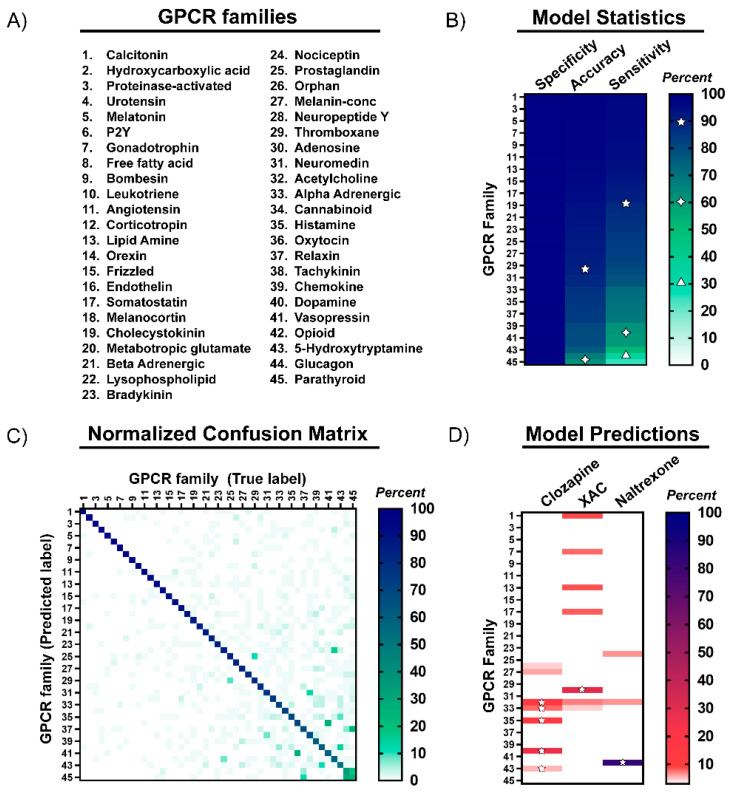
Metrics for machine learning model performance and example predictions. (**A**) GPCR families associated with the numbering scheme used in the figure. (**B**) Accuracy, sensitivity, and specificity metrics (%) of model predictions for compounds in the test dataset were calculated for each family and shown as a heatmap. Indicators of specific values in the legend for 30%, 60%, 90% (triangle, diamond, and star, respectively) are shown within each heatmap as a visual reference. (**C**) Confusion matrix comparing classification of compounds in the test dataset into their predicted versus true (annotated) GPCR target family. The value at each position in the heatmap represent the percentage of compounds in the test set classified into each GPCR family. (**D**) Example of model predictions for three unmodified molecular scaffolds. Model predictions are shown as a heatmap specifying the classification probability (%) for each GPCR family. Stars are used to mark predictions of known GPCR family interactions. Numerical values for all heatmaps are included as tables in Figures S3–S5.

To understand our model’s performance in more detail, we calculated accuracy, sensitivity, and specificity metrics for each GPCR family (Figure 2B, Figure S3). Accuracy on a per-family basis was calculated as the percentage of compounds correctly classified to their target family, using their GLASS database annotation as the ground truth. Sensitivity and specificity were calculated as the rate of positive and negative interactions that were correctly classified, respectively. We note that given the large number of true negative interactions being predicted across GPCR families, the specificity metric was potentially biased toward high values.

We found that the model had 60% or better classification accuracy across all GPCR families in our test set with 30/45 families exhibiting greater than 90% accuracy, indicating a high level of performance for the majority of compounds in our test set. Sensitivity for the same 30 GPCR families was 80% or better. Notably, the sensitivity values for Glucagon and Parathyroid receptors were below 50%, which is likely due to the high rate of misclassification among compounds targeting these two families, as discussed below. Finally, all GPCR families in our test set exhibited specificity values greater than 98%, consistent with the high values that might be expected for multiclass classification tasks with predominantly negative predictions.

Analysis of test set predictions as a confusion matrix provided additional insights into model performance for individual GPCR target families (Figure 2C, Figure S4). For most families, model accuracy was high with small errors dispersed among classes. In the few cases with higher levels of misclassification, errors could often be attributed to compounds targeting closely related families. For example, the model frequently misclassified compounds targeting the Vasopressin and Oxytocin families, which interact with similar peptide ligands and share functions in neural cognition and behavior [31] (Figure S4). Similarly, the model misclassified compounds targeting the Glucagon and Parathyroid families, which are also peptide-binding receptors that share a high level of similarity in their structural mechanism for ligand binding [32]. Therefore, the low sensitivity for these families could be explained by the natural promiscuity of their ligands. In addition, compounds targeting these families displayed a diffuse spatial pattern in UMAP space, indicating a high degree of heterogeneity in their molecular fingerprints, which is challenging for identification of family-specific patterns. While our model delivered good classification accuracy across many peptide and lipid binding GPCRs, we cannot exclude the possibility that our use of molecular fingerprint designed for small molecules may have contributed to lower sensitivity for some receptors that naturally bind peptides, polypeptides, or lipids. Further refinement of the machine learning strategy to accommodate the unique properties of ligands targeting these specific GPCR families would be expected to improve the sensitivity of the model. This could be achieved by training models with molecular fingerprints representations optimized for specific ligand types, although exploring this strategy was outside the scope of this study.

### 2.6. Extending Model Predictions to Identify Promiscuous GPCR Ligands

Having observed good classification accuracy on our test set, we extended the use of our model’s predictions to identify compound promiscuity across GPCR families. Up to now, for each compound, the GPCR family with the highest classification probability was chosen as the predicted target class. We hypothesized that the generalizable principles associating molecular fingerprint patterns with specific GPCR families might be reflected in the classification probability distribution across families, thereby providing insight into a compound’s propensity for promiscuous binding. Accordingly, to screen for multi-target binders we used an empirically determined minimum threshold to include lower probability predictions as potential interactions. Repurposing the classification probabilities output of our random forest model in this way enabled us to extract additional information and predict compound binding to all 45 GPCR target families in our dataset.

To evaluate use of the model for this purpose, we tested its ability to predict interactions for three known GPCR ligands chosen on the basis of their varying selectivity for different GPCR families: Clozapine [33], Xanthine amine congener (XAC) [34], and Naltrexone [35]. Clozapine, an atypical antipsychotic drug, represents a known promiscuous ligand that interacts with receptors in the Acetylcholine, alpha-Adrenergic, Histamine, Dopamine, and 5-Hydroxytryptamine families. In contrast, XAC and the addiction treatment drug Naltrexone are regarded as family-selective ligands of Adenosine and Opioid receptors, respectively. For all three ligands, the model’s classification probabilities output successfully predicted their known GPCR target families (Figure 2D and Supplementary Materials Figure S5). For Clozapine, the model predicted interactions with all five of its known GPCR target families in addition to two unvalidated interactions with Orphan and Melanin-concentrating receptors. For XAC and Naltrexone, the highest predicted probability matched their known GPCR target families. Other predictions above the designated probability threshold identified few additional unvalidated target families. Several of these unknown interactions were later confirmed by NanoBRET ligand-engagement screens, demonstrating the value of our machine learning-guided approach for scaffold selection (see Results below). Together, these examples confirmed that the model could predict known interactions of both selective and promiscuous GPCR ligands, making it a useful in silico screening tool for identifying candidate scaffolds for development into NanoBRET tracers. With that, we extended the model’s predictions to include binding profiles for a total of 17 model scaffolds (Supplementary Materials Figure S6).

### 2.7. Synthesis of Fluorescent Tracers from Selected Scaffolds

Once suitable scaffolds are selected, the next step toward generation of fluorescent tracers is synthesis of their modifiable analogues. It is rather rare that a compound possesses an easily modifiable group at a position that tolerates modification without impact on binding properties. Often, introduction of a modifiable functionality (usually an amino-group for amide coupling) requires either de novo total synthesis or application of sophisticated late-stage chemistries. In addition, in many cases a site suitable for modification without significant impact on binding properties is unknown, requiring empirical evaluation of several sites. Below we highlight several examples taking advantage of different synthetic strategies to generate modifiable analogues via a reasonable number of steps.

**Figure 3:**
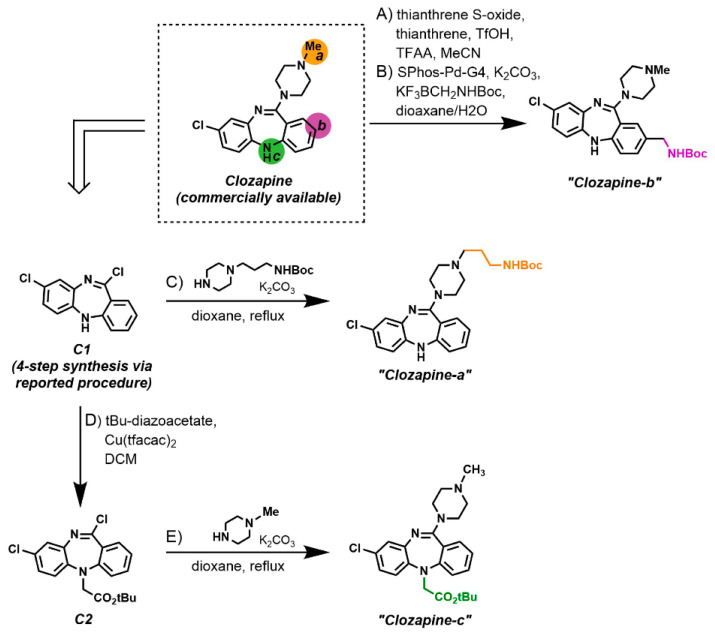
Synthesis of modifiable Clozapine analogues. (**A**,**B**) Boc-protected “Clozapine-b” was prepared from commercially available Clozapine by a two-step procedure utilizing late-stage Ritter arene thianthrenation followed by Suzuki-Miyaura cross-coupling with potassium Boc-aminomethyltrifluoroborate. (**C**) Boc-protected “Clozapine-a” was synthesized from advanced intermediate C1 via reaction with 1-(3-*N*-Boc-propyl)-piperazine. (**D**) tBu-protected “Clozapine-c” was generated via copper-catalyzed carbenoid insertion of tBu-diazoacetate into aniline N-H bond of C1 followed by (**E**) imidoyl chloride substitution with N-methylpiperazine.

Clozapine represents an example of our approach evaluating a mixture of rational and empirical modifications sites (Figure 3). Multiple SAR analyses for Clozapine and its main targets suggest that position “a” might be suitable for fluorophore attachment [36]. This speculation is further supported by the structurally related atypical antipsychotic drug Quetiapine having a PEG unit at position “a”. At the same time, it is possible that a fluorophore appendage at this site might negatively impact the interactions of Clozapine with some of its targets. With this in mind, we devised a strategy to generate three Clozapine derivatives, each one with a different modifiable site (Figure 3). Clozapine-b, containing a protected amino-methyl group for further modification through amide bond formation, was generated via late-stage modification of commercially available Clozapine. The two-step synthesis started with Ritter late-stage aromatic C−H functionalization [37] followed by the attachment of a protected amino-methyl handle [38]. Both Clozapine-a and Clozapine-c were synthesized from advanced intermediate C1, which was generated via a reported 4-step synthetic scheme [33]. Reaction of C1 with appropriately substituted piperazine delivered Clozapine-a. Similarly, Clozapine-c was generated by a two-step sequence from C1.

Amitriptyline, a promiscuous tricyclic antidepressant [39], is another example of our empirical exploration approach (structure and modifiable sites are portrayed in Figure 6A). Nortriptyline, its commercially available demethylated analogue is amendable to facile modification at its secondary amine. However, a recently published crystal structure of dopamine transporter with bound Nortriptyline [40] suggests that the aromatic rings of Amitriptyline/Nortriptyline might be more appropriate modification sites. From a synthetic point of view, generating multiple modifiable analogues of Amitriptyline is somewhat challenging due to its pseudo-symmetry, which may result in an inseparable mixture of isomers. In addition, the presence of a reactive exocyclic double bond could render many late-stage chemistries inapplicable. We sought to employ a recently reported elegant approach to Amitriptyline’s skeleton [41], which could provide a straightforward path to aryl-substituted Amitriptylines. Indeed, this approach delivered four amine-reactive carboxy-modified Amitriptylines in a fast and expedient manner (Figure S7).

The modification of another scaffold AZD1283, a potent antagonist of the P2Y12 receptor [42] is a good example of the synthetic advantages of late-stage modifications. A quick inspection of AZD1283’s structure identified an ethyl ester as a synthetically accessible site for modification and fluorophore attachment. However, both the reported SAR [42] and analysis of P2Y12’s crystal structure in complex with AZD1283 ([43], PDB 4NTJ) suggested that the phenyl ring on the opposite side of the molecule is a better candidate for modification (structure and modifiable site are portrayed in Figure 6C). From a synthetic point of view, late-stage modification of commercially available AZD1283 was especially attractive considering the lengthy total synthesis of an AZD1283 analogue carrying a modifiable group on its phenyl ring. Using late-stage alkenylation chemistry developed by Yu and coworkers, [22] commercial AZD1283 was modified at the ortho position of the phenyl ring (due to the directing effect of AZD1283’s sulfonamide), albeit at only 4% yield (Figure S8). Despite the low yield, the unoptimized reaction delivered milligram quantity of modified AZD1283, which was sufficient for subsequent development of fluorescent tracers. This example illustrates the strategic advantage of applying late-stage chemistries to tracer discovery as it often allows generation of modifiable analogues in a faster and cheaper manner than de novo synthesis.

Using a blend of different synthetic strategies similar to those outlined above, we generated modifiable analogues for the 17 model scaffolds and conjugated them to a fluorophore via amide-type couplings. We chose the red emitting NanoBRET 590 dye (E_ex_ 576 nm; E_em_ 589 nm) for a fluorophore due to its photochemical properties, including small size, chemical stability, and sufficient spectral overlap with NanoLuc emission, which is critical for efficient energy transfer [16] (Figure S9). The length and nature of the linker connecting NanoBRET 590 to the modifiable scaffold can also influence the efficiency of energy transfer as well as the physicochemical properties of the tracer (e.g., solubility). We tuned the tracer’s properties with a set of three different linkers (Supplementary Materials Figure S9). The shortest linker tested was a direct attachment of NanoBRET 590 to the modifiable scaffolds. The two other linkers are a PEG-4 linker that provides extended length between a molecular scaffold and NanoBRET 590 with an added benefit of increased aqueous solubility and a linker comprising one PEG unit and a benzylamide unit, coupling the solubilizing properties of PEG with the rigidity and hydrophobicity of a benzene ring. We opted to first generate tracer candidates incorporating the PEG-4 linker to provide a spacer between a scaffold and NanoBRET 590. When necessary, we further optimized the properties and binding affinity of these candidates by exchanging PEG-4 with other linkers. We found empirically that incorporation of a hydrophobic moiety near NanoBRET 590 was often beneficial to tracer performance. In the end, we generated several tracer candidates for each one of the 17 scaffolds and report here the synthesis and evaluation of a selection of the best-performing sets (Supplementary Materials Table S2 and Excel spreadsheet).

### 2.8. Evaluation of Binding Profiles for Fluorescent Tracers in a Cellular NanoBRET Screen

To evaluate the binding profile of each tracer candidate, we generated a library of 184 DNA constructs expressing GPCRs genetically fused to an N-terminal HiBiT. For consistency and efficient translocation to the cell surface, native secretion signals were removed, and an IL6 secretion signal sequence was incorporated upstream of HiBiT. Additionally, to insure HiBiT integrity upon signal peptide removal, a two amino acid valine–serine spacer was inserted between the signal sequence and HiBiT [5].

**Figure 4:**
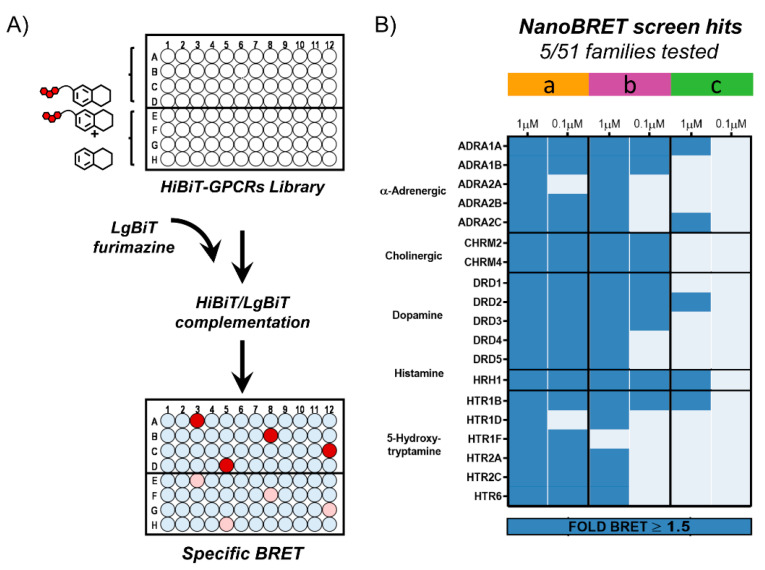
NanoBRET screen of Clozapine tracer candidates. (**A**) Illustration of the NanoBRET screening strategy for revealing the binding profile of fluorescent tracer candidates. Duplicates of cells transfected with 48 different HiBiT-GPCRs constructs per plate were treated with a fluorescent tracer either alone or in the presence of competing parental compound. Following complementation with purified LgBiT and measurement of BRET, specificity of interactions was evaluated through a decrease in BRET due to competitive displacement of the bound tracer by excess parental compound. Background BRET is shown in light blue, specific BRET is shown in red and a decrease in BRET due to competitive displacement is shown in lighter pink. (**B**) Binding profile of Clozapine tracer candidates. Modification sites are shown in Figure 3. NanoBRET screen across 184 HiBiT-GPCRs from 51 families revealed specific interactions (≥1.5-fold response) with GPCRs from five different families. Clozapine-a-2-NB590 had the broadest binding profile.

Each tracer candidate was screened across the entire library of HiBiT-GPCR constructs using NanoBRET as the detection modality. As depicted in Figure 4A, the specificity of interactions was evaluated through decrease in BRET due to competitive displacement of a bound tracer by excess parental compound. Each tracer was analyzed for binding to each HiBiT-GPCR fusion in the absence and in the presence of competing parental compound. Briefly, HEK293 cells were transfected with HiBiT-GPCR constructs diluted into promoterless DNA to reduce expression and maximize translocation to the cell surface. Each tracer candidate was then screened at concentrations of 1 μM and 0.1 μM across cells expressing these fusions. Cells were treated for 1.5 h with tracer either alone or in the presence of 20 μM competing parental compound (i.e., control). BRET was measured upon addition of purified LgBiT and subsequent complementation with HiBiT genetically fused to a GPCR. The specificity of interactions was assessed through a decrease in BRET signal due to competitive displacement of the bound tracer. To further gauge the robustness of these interactions, the fold response over the control (i.e., BRET_tracer_/BRET_control_) was calculated. We chose a 1.5-fold response, which translates to ≥7-fold signal over noise as the minimal threshold required to score an interaction as positive. The compiled screening results for all fluorescent tracer candidates across 184 GPCRs from 51 different families and their structures are included in the Supplementary Materials (Excel spreadsheet). Below we highlight the screening results obtained for our exemplary fluorescent tracers based on Clozapine, Amitriptyline and AZD1283.

Analyses of all three Clozapine tracers revealed specific interactions with 19 GPCRs from the five expected families, albeit with a different binding profile for each one (Figure 4B). These tracers were generated by conjugation of NanoBRET 590 to modifiable Clozapines (Clozapine a–c; Figure 3) via a PEG4 linker (linker 2; Figure S9). Clozapine-c-2-NB590, modified on the diazepine’s nitrogen, had a poor binding profile that was limited to five GPCRs at the higher 1 μM tracer concentration. The binding profile for Clozapine-b-2-NB590, modified on the phenyl ring, was broadened to 18 GPCRs. However, only half of them retained a ≥1.5-fold response at the lower 0.1 μM tracer concentration. Clozapine-a-2-NB590, modified on the piperazine ring, had the broadest binding profile. It exhibited specific binding interactions with all 19 GPCRs and retained with 17 of them a ≥1.5-fold response at the lower 0.1 μM tracer concentration. Altogether, this analysis showed that modification of the piperazine ring, which is supported by SAR [36], is tolerant to attachment of a fluorophore.

**Figure 5:**
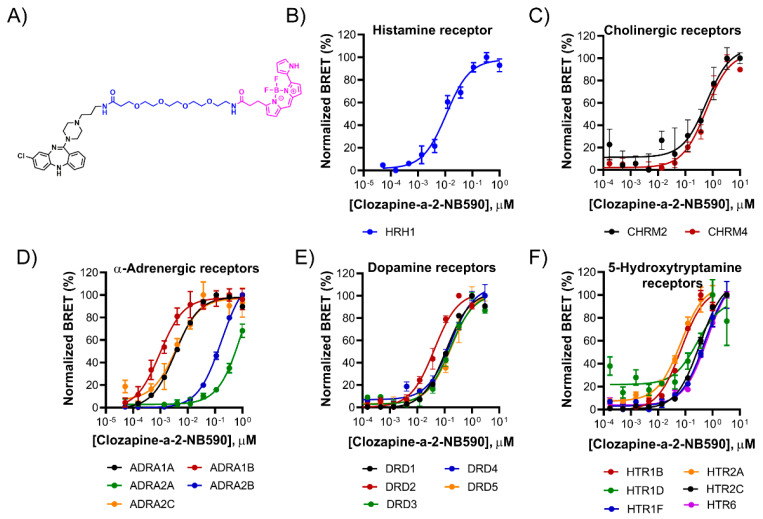
Saturation binding of Clozapine-a-2-NB590 to HiBiT-GPCRs from five families. (**A**) Structure of Clozapine-a-2-NB590. (**B**–**F**) Specific dose-dependent BRET measurements for HiBiT-GPCRs expressed in HEK293 cells. BRET values at each Clozapine-a-2-NB590 tracer concentration were background-corrected by subtracting parallel measurements taken in the presence of competing Clozapine. Data represent the mean ± S.D. of triplicates.

Encouraged by the broad binding profile of Clozapine-a-2-NB590 (Figure 5A), it was further evaluated for concentration dependent binding to the 19 GPCRs identified as interactors (Figure 5B–F). HEK293 cells expressing the 19 HiBiT-GPCRs were treated with increasing concentrations of Clozapine-a-2-NB590 alone or in the presence of competing Clozapine (30 μM). Background corrected BRET measurements showed robust, specific and concentration-dependent binding for 17 of the 19 GPCRs, exhibiting a wide range of EC_50_ values (1–600 nM). Concentration-dependent binding to the remaining GPCRs, ADRA2A and HTR1D, was specific but suffered from high background, likely due to nonspecific BRET originating from random interactions. Interestingly, in the NanoBRET screen, interactions between Clozapine-a-2-NB590 and these 17 GPCRs retained a ≥1.5-fold response at the lower 0.1 μM concentration while interactions with ADRA2A and HTR1D did not. This suggests a correlation between the retention of a ≥1.5-fold response at the lower 0.1 μM tracer concentration and the likelihood for a robust saturation ligand-engagement assay.

**Figure 6:**
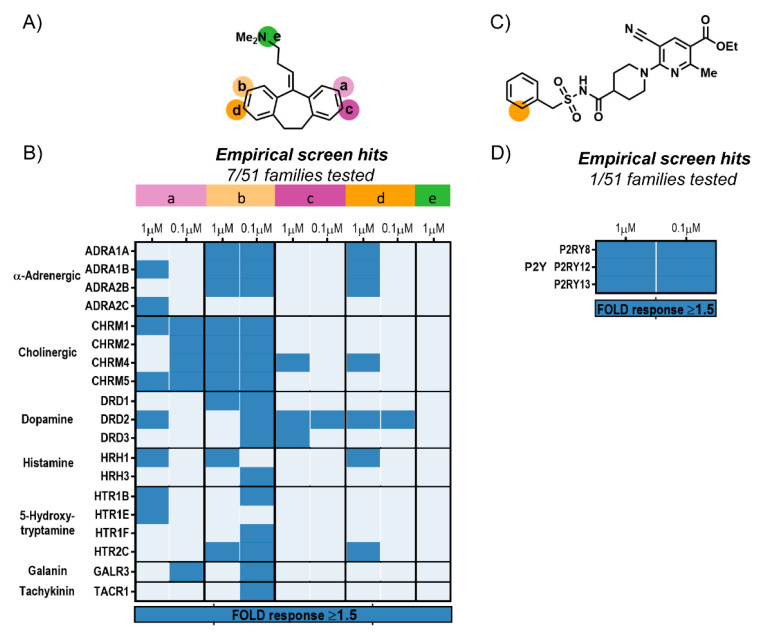
NanoBRET screen of Amitriptyline and AZD1283 tracer candidates. (**A**) Structure of Amitriptyline with modifiable positions marked. (**B**) NanoBRET screen across 184 HiBiT-GPCRs from 51 families revealed specific interactions (≥1.5-fold response) with GPCRs from seven families. Amitriptyline-b-2-NB590 had a substantially broader binding profile exhibiting specific interactions with 17 GPCRs. (**C**) Structure of AZD1283 with modifiable position marked. (**D**) NanoBRET screen revealed specific interactions (≥1.5-fold response) with 3 Purinergic GPCRs.

The benefit of evaluating several modification sites was further demonstrated for Amitriptyline where we observed significantly different binding profiles among the five tracer candidates (Figure 6A,B). These tracer candidates were generated by conjugation of NanoBRET 590 to modifiable Amitriptylines (Figure 6A; Amitriptyline a–e) via a PEG4 linker (linker 2). Amitriptyline-e-2-NB590, modified on the amine nitrogen of commercial Nortriptyline, resulted in a poor tracer with no specific interactions. Although the other four tracer candidates were generated through modification of positions just one atom apart on the two quasi-symmetric phenyl rings, only one of them provided a substantially broad binding profile. Amitriptyline-b-2-NB590 displayed specific binding interactions to 17 GPCRs from seven different families, with 16 of them exhibiting a ≥1.5-fold response at the 0.1 μM tracer concentration. The binding profiles of the other three candidates were more limited with detectable interactions predominately at the 1 μM tracer concentrations.

For AZD1283, the third tracer scaffold, late-stage alkenylation enabled conjugation of NanoBRET 590 to the phenyl ring with minimal synthetic effort (Figure 6C,D). NanoBRET screen revealed selective binding to the Purinergic receptor family (Figure 6D), which was in agreement with the machine learning predictions. Interestingly, in addition to the known interaction with P2RY12, the screen uncovered two previously unreported interactions with P2RY8 and P2RY13. Notably, all three interactions exhibited a ≥1.5-fold response at the two tracer concentrations tested.

Altogether, the NanoBRET screen of tracer candidates for 17 different scaffolds uncovered specific binding interactions for 58 GPCRs from 18 different families (Figure S10). The screen identified 160 individual interactions with 77% of them retaining ≥1.5-fold response at the 0.1 μM tracer concentration. Notably, several of those verified interactions, which were not described before (30%), uncovered unexpected and unreported promiscuity for well-studied GPCR ligands considered to be selective such as Aminopotentidine, NAN-190, XAC and Tolterodine.

### 2.9. Comparing Machine Learning Predictions and Empirical NanoBRET Evaluations

**Figure 7:**
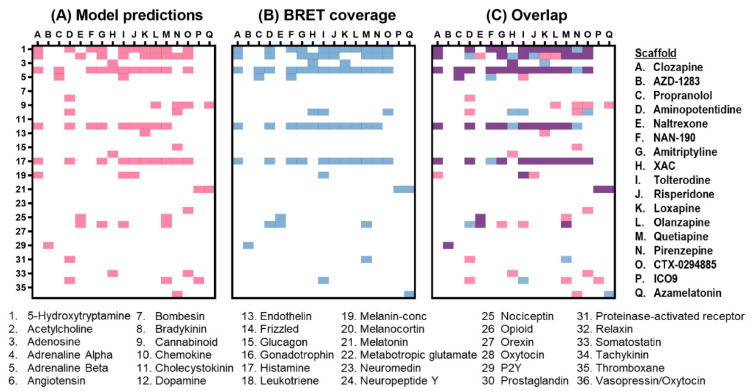
Machine learning predictions for GPCR-ligand interactions versus empirical NanoBRET screens. (**A**) Machine learning predictions for interactions between scaffolds A–Q (specified on the right) and 36 GPCR families. For each scaffold, predictions receiving a classification probability ≥4% were considered a positive interaction and are shown in red, with predicted negative interactions in white. (**B**) Empirical NanoBRET evaluations across the 36 GPCRs families using fluorescent tracers. For each scaffold, confirmed interactions with ≥1.5-fold response are shown in blue and negative or unconfirmed interactions are shown in white. (**C**) Concordance between machine learning predictions and NanoBRET screens. Confirmed predictions are shown in purple.

Finally, we opted to compare the machine learning predictions for the 17 scaffolds to the empirical NanoBRET evaluations across 36 GPCR families investigated by both strategies (Figure 7). The highest correlation between model-predicted and NanoBRET-confirmed interactions was found using minimal thresholds of ≥4% classification probability and ≥1.5-fold response, respectively (Figure S11). These thresholds provided the best balance between model accuracy and a low percentage of false predictions. Accordingly, machine learning predictions for all unmodified scaffolds were compiled (Figure S6) and those above the minimum 4% probability threshold were classified as positive interactions (Figure 7A). At the same time, empirical NanoBRET results for each scaffold were consolidated to include results for all relevant fluorescent tracer candidates screened at either 1 μM or 0.1 μM tracer concentrations. Screening results were further grouped by families and considered positive if at least one member had a ≥1.5-fold NanoBRET response (Figure 7B). One caveat comparing model predictions to empirical NanoBRET evaluations is their use of unmodified versus fluorescently modified scaffolds, respectively. Therefore, model predictions not verified by BRET could in part be the result of fluorophore interference with binding interactions. Where feasible, we sought to address this possibility and further verify interactions for unmodified scaffolds via competitive displacement of other fluorescent tracers already shown to bind the relevant targets. For example, using Clozapine-a-2-NB590 we were able to verify additional interactions for Aminopotentidine with alpha-Adrenergic, Dopamine and 5-Hydroxytryptamine receptors as well as additional interactions for XAC with alpha-Adrenergic receptors. These additional verifications were incorporated to the comparative analysis and are also included in the compiled screening results (Supplementary Materials Excel spreadsheet).

Overlap analysis between machine learning predictions and empirical NanoBRET evaluations (Figure 7C) revealed that 568 out of the total 612 predictions were confirmed by BRET (i.e., 92.8% accuracy). However, given the high number of negative interactions that were predicted and confirmed, this accuracy value is likely biased toward a high value. Indeed, 512 of the 530 (97.2%) negative interactions and 56 of the 82 (68.3 %) positive interactions predicted by the model were confirmed by NanoBRET. The corresponding specificity (94.9%) and sensitivity (76.7%) metrics demonstrate the overall effectiveness of this workflow for tracer discovery.

### 2.10. Summary

**Figure 8:**
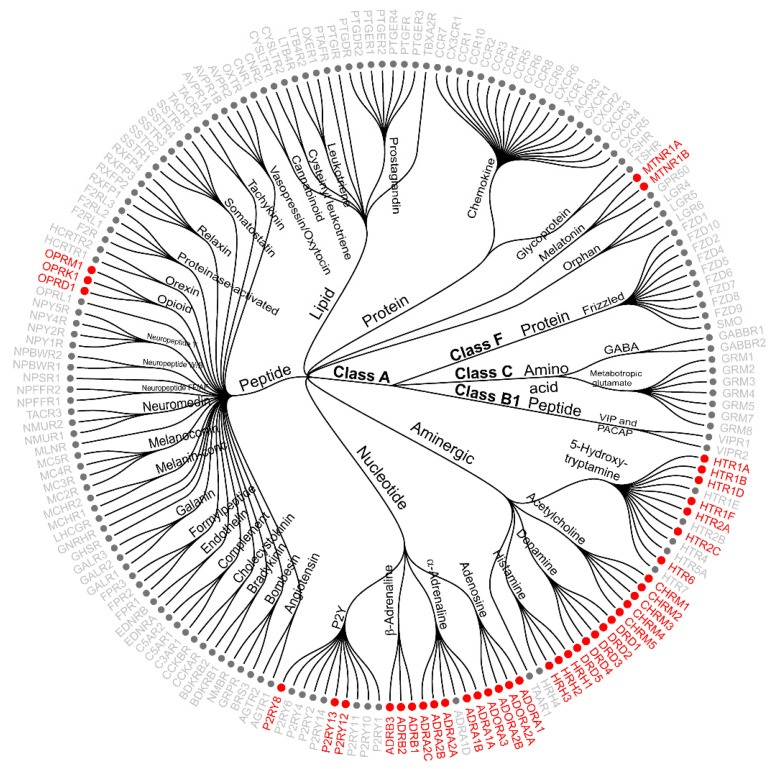
NanoBRET GPCR ligand-engagement assays facilitated by lead fluorescent tracers. A circular dendrogram showing GPCRs classified according to their family and class types [1]. NanoBRET ligand-engagement assays for the GPCRs highlighted in red were facilitated by lead fluorescent tracers developed in this study. Details on these assays including, the fluorescent tracers associated with each assay as well as tracers’ binding affinity and structures are included in Figures S12 and S13.

The high specificity of NanoBRET is particularly attractive when combined with the versatility of promiscuous fluorescent tracers, alleviating the burden of developing a specific tracer for each target. Here, we developed an integrated tracer discovery workflow coupling machine learning-guided in silico screening for promiscuous tracer scaffolds with a blend of synthetic strategies to rapidly generate multiple tracer candidates. Using this workflow, we developed over a dozen fluorescent tracers that can effectively engage several GPCRs from one or more families. A limited set of 10 leading tracers enabled robust saturation ligand-engagement assays for 40 GPCRs, including some overlapping coverage among them (Figure 8). Our screen uncovered interactions with 11 other GPCRs that retained a ≥1.5-fold response at lower 0.1 μM tracer concentrations. Further optimization of the associated tracers’ linker chemistry or conjugation site could expand this coverage, particularly for GPCRs that naturally bind peptides and polypeptides, but was beyond the scope of this study. We believe that our strategy has the potential to accelerate fluorescent tracer discovery and reduce the synthetic burden associated with their development. Furthermore, this approach could be applied to other target classes beyond GPCRs.

## 3. Materials and Methods

Supplementary Materials includes additional details regarding reagents, DNA constructs, cell culture, transfection, and NanoBRET saturation ligand-engagement assay.

### 3.1. Data Collection and Pre-Processing for Machine Learning

Chemical interaction data was downloaded from the GLASS database (https://zhanglab.ccmb.med.umich.edu/GLASS/) in 3 October 2018. All data processing was performed in R version 3.6.3. The raw interaction data was filtered to select compounds that target human GPCRs, have an assigned CHEMBL ID number, and their chemical structure represented in SMILES format [44]. The resulting dataset had a total of 304,564 target-ligand interactions across 230 GPCRs. To facilitate analysis of the data structure and reduce the number of classes required for machine learning each interaction was further assigned into an established GPCR family based on its target receptor [1]. The dataset contained 56,902 unique compounds, with the most frequent molecule occurring 120 times. To reduce biases in target-ligand interaction data and maximize the chemical diversity available for machine learning, data was filtered for unique compound-GPCR family combinations, resulting in a “Filtered” dataset of 136,848 unique molecular interactions with 227 GPCRs. In the resulting dataset, several GPCR families had only a small number of described interactions, therefore, only families having interactions with at least 700 unique compounds were selected for further analysis. This minimum threshold was chosen to balance molecular diversity against total number of training examples for each family.

A final “Selected” dataset was created by random selection of 530 target-ligand interactions for each GPCR family from the Filtered dataset, preserving equal class balance across family categories. The Selected dataset had 23,850 unique target-ligand interactions distributed equally among 45 GPCR families. This dataset was randomly split 70/30 into training and test sets where each received 16,695 and 7155 interactions, respectively. After conversion of compounds from SMILES format to molecular fingerprints, the training set was used as input to train the machine learning model while the test set was held out for subsequent validation of the final model.

### 3.2. Data Representation using Molecular Fingerprints

A combination of three molecular fingerprinting strategies was used to capture different representations of each compound in our dataset and maximize the information available to the machine learning algorithm during training. Compounds in SMILES format were converted to a single numerical vector of 758 fingerprints comprising 166-bit MACCS keys [26], 79-bit complete E-State atom types [27], and 512-bit Open Babel FP4 fingerprints [45] using the RCPI package version 1.20.1 in R [46].

Since the time required to train machine learning models can be lengthy and is partially dependent on the number of features representing the data, the number of fingerprints was reduced by removing those that were either unused or had low variance in our training dataset. This allowed for faster model training and improved the model’s ability to fit the data by protecting against highly imbalanced features that might exert undue influence during training. Using the caret package version 6.0-86 in R [47] near-zero variance features were eliminated, resulting in a reduction from 758 to 187 fingerprints representing compounds in the training and test datasets. Molecular information represented as fingerprints was then used as the input for training the machine learning model.

### 3.3. UMAP Clustering of Molecules in the Training Dataset

Uniform Manifold Approximation and Projection (UMAP) is a general-purpose manifold algorithm for dimensional reduction [29]. UMAP occurs in two phases. First, a weighted k-nearest neighbor graph is computed on features of the dataset and second, a low-dimensional layout of the graph is calculated. The resulting embedded datapoints preserve the high-dimensional structure of the data but allow it to be visualized in lower dimensions and make comparisons with other variables of interest.

The MACCS molecular fingerprint representation of the training dataset was used as input and the graph was computed using the UMAP package version 0.2.5.0 in R. Since the resulting spatial distribution following UMAP dimensionality reduction is dependent on the user-defined number of nearest neighbors, a range of settings from 10–400 neighbors were explored. A value of 50 nearest neighbors provided the optimal spread of the data in low dimensions suitable for visualization while retaining the clustering of compounds according to their target GPCR families. All UMAP visualizations were created using the ggplot2 package version 3.3.0 in R [48].

### 3.4. Training the Machine Learning Model

Machine learning model training was carried out using the caret package version 6.0-86 in R. Employing the training set in molecular fingerprint format as the input, a random forest classification model was trained for accuracy using 5-fold cross-validation repeated 10 times. Software defaults were used for automatic data centering, scaling, and hyperparameter tuning during the training phase (see Supplementary Materials). The training process created a model fitting target classes (GPCR families) as a function of the molecular fingerprints of their interacting compounds, using their annotation from the GLASS database as the ground truth.

Performance metrics such as accuracy, sensitivity, and specificity were calculated automatically from model predictions of target GPCR family for compounds in the test set using their specific interactions in the GLASS database as ground truth. Using measurements for true positive (TP), true negative (TN), false positive (FP), and false negative (FN), the formulas used were Accuracy = (TP + TN)/(TP + FN + TN + FP), Sensitivity = TP/(TP + FN), and Specificity = TN/(TN + FP). Statistics at the GPCR-family level were used to create a confusion matrix evaluating the accuracy of model prediction on target-ligand interactions for individual families. All statistical metrics were calculated automatically following model training using caret in R.

### 3.5. Machine Learning Model Implementation and Comparison with NanoBRET Data

To predict the GPCR target family for compounds used as scaffolds for fluorescent tracer development, the chemical structure of each unmodified compound was converted to SMILES representation using ChemDraw Professional version 16.0. Molecular fingerprints were extracted as outlined earlier in Methods using the RCPI package in R. The machine learning model was imported into an R environment and used to predict the GPCR family class probabilities for each scaffold.

Class probabilities, converted to values ranging from 0 to 100% for readability, were used as a guide to search for potential interactions among compounds and GPCR families. A threshold of 4% and above for positive interactions provided the best correlation between the machine learning predictions and empirical NanoBRET data.

### 3.6. Robotic NanoBRET Screening

HiBiT-GPCR DNA constructs were diluted in 10 mM Tris pH 8 to a final concentration of 0.01 μg/μL, arrayed in 96-well plates and stored at −20 °C. For transfection, HEK293 cells were suspended in Opti-MEM (without phenol red) supplemented with 2% fetal bovine serum (Seradigm) and 100 units/mL penicillin–streptomycin (Gibco) at a final concentration of 220,000 cells/mL and dispensed into white 96-well plates at 90 μL/well. Subsequent transfections and screens were carried using an Agilent Bravo Automated Liquid Handling Platform. First, the 96-well HiBiT DNA arrays were diluted 25-fold in Opti-MEM (without serum or phenol red) to a final concentration of 0.0004 μg/μL and 20 μL of the diluted DNAs were transferred into new 96-well plates. Next, promoterless carrier DNA (1 μg/μL) was diluted 25-fold (0.04 μg/μL) and then dispensed into the diluted arrays at 20 μL/well. Following brief mixing, Viafect transfection reagent (Promega) diluted 16-fold into Opti-MEM was dispensed into the plates at 40 μL/well. After mixing by pipetting, plates were incubated for 10 min on the pipetting station deck. The DNA-transfection reagent complexes were then added to plates containing cells. To facilitate the screen, 10 μL of DNA-transfection reagent complexes were added in duplicates to the top 4 and bottom 4 rows, resulting in two identical 48-well arrays per plate. Plates with transfected cells were incubated for 18–20 h at 37 °C/5% CO_2_. A screen of each tracer across the entire library using two different concentrations requires duplicate plates of transfected cells. Depending on the number of tracers being screened, transfections can be scaled up using the same ratios. The next day, for each tracer, four 20× screening solutions in tracer dilution buffer (Promega) were prepared: two duplicates at final concentration of 20 μM and two duplicates at a final concentration of 2 μM. A competing unmodified compound was added to one of each duplicate at a final concentration of 400 μM. The four resulting solutions were diluted 4-fold in Opti-MEM without serum or phenol red to generate 5× solutions (5 µM and 0.5 µM tracer +/− 100 μM competing compound). To screen the library, a BioTek MultiFlo FX liquid dispenser was used to dispense 22.5 µL of tracer solution to the top 4 rows and 22.5 µL tracer + competing compound solution to the 4 bottom rows. Plates were briefly mixed on an orbital shaker and incubated for 1.5 h at room temperature. The MultiFlo FX liquid dispenser was used to dispense 2× detection reagent (100-fold dilution of LgBiT (Promega) and 50-fold dilution of furimazine live cell substrate (Promega) in Opti-MEM) at 125 μL/well. Plates were briefly mixed on an orbital shaker and incubated for 15 min to allow complementation. Filtered luminescence was measured using a GloMax Discover microplate reader (Promega) equipped with a 450-nm (8-nm band pass) filter (donor) and a 600-nm long pass filter (acceptor). BRET was calculated by dividing the acceptor emission by the donor emission. Fold response was calculated for each construct at each tested tracer concentration by dividing BRET from wells treated with tracer alone (BRET_tracer_) by BRET from duplicate wells treated with tracer + competing compound (BRET_control_). The minimal threshold for specific interactions was 1.5-fold response, which translates to ≥7-fold signal over noise. Signal over noise was calculated for each interaction in a limited set of triplicate screens using the formulation:

Signal/noise = (BRET_trace_ − BRET_control_)/STDEV(BRET_control_) (1)

## Data Availability

The data presented in this study is openly available and is included as part of the Supplementary Materials.

## Supplementary Materials

The following are available online. Supplementary Methods, Figures and Tables, Synthetic procedures and Compound characterization (PDF); NanoBRET screen results (Excel); Glass dataset (csv); Machine learning script (R).

## Sample Availability

Samples of the compounds are not available from the authors.
