# Supplementary material for: An Integrated Approach toward NanoBRET Tracers for Analysis of GPCR Ligand Engagement
Source: Molecules. 2021 May 12;26(10):2857. doi: 10.3390/molecules26102857 (PMC8151276; doi:10.3390/molecules26102857)
Supplement: Supplementary file 1 [file molecules-26-02857-s001.zip › molecules-1205027-supplementary/molecules-1205027-supplementary.pdf]

SUPPLEMENTARY INFORMATION

An Integrated Approach Toward NanoBRET Tracers for Analysis of GPCR Ligand Engagement

Michael P. Killoran[1], Sergiy Levin[2], Michelle E. Boursier[1], Kristopher Zimmerman[1], Robin Hurst[1], Mary P. Hall[1], Thomas Machleidt[1], Thomas A. Kirkland[2] and Rachel Friedman Ohana[1*]

1. Promega Corporation, 2800 Woods Hollow, Fitchburg, WI, 53711, USA; mike.killoran@promega.com; michelle.boursier@gmail.com; Kris.Zimmerman@promega.com; robin.hurst@promega.com; Mary.Hall@promega.com; Thomas.Machleidt@promega.com; rachel.ohana@promega.com

2. Promega Biosciences LLC, 277 Granada Drive, San Luis Obispo, CA, 93401, USA; serge.levin@gmail.com; thomas.kirkland@promega.com

* Correspondence: rachel.ohana@promega.com; Tel.: (608) 274-1181.

Supplementary Information is divided into two parts: Part 1 includes Supplementary Methods and Supplementary Figures and Tables, which are listed according to the main text and the main figures to which they are related. Part 2 includes Synthetic Procedures and Compound Characterization.

SUPPLEMENTARY INFORMATION PART 1 (METHODS, FIGURES AND TABLES)

Supplementary Methods

- Reagents
- DNA constructs
- Cell culture and transfection
- NanoBRET saturation ligand-engagement assay

Supplementary Figures and Tables:

- Table S1. GPCR families in the GLASS dataset as numbered in Figure 1.
- Figure S1. Performance of machine learning models using reduced training set sizes.
- Figure S2. Classification accuracy for machine learning models utilizing different molecular fingerprint representations of the training set.
- Figure S3. Performance metrics for machine learning model on a per-family basis.
- Figure S4. Confusion matrix on a per-family basis of machine learning model classification predictions.
- Figure S5. Machine learning model predictions of interactions for three unmodified scaffolds.
- Figure S6. Machine learning model predictions for all unmodified scaffolds.
- Figure S7. Synthesis of modifiable Amitriptyline analogues.
- Figure S8. Synthesis of modifiable AZD1283 analogue.
- Figure S9. Structures of NanoBRET 590 and linkers connecting it to selected scaffolds.
- Table S2. Fluorescent tracers generated and characterized in this study.
- Figure S10. Binding profile of the leading tracer for each selected scaffold.
- Figure S11. Comparison of thresholds for machine learning model predictions and NanoBRET screens.
- Figure S12. Summary of GPCRs NanoBRET engagement assays and their lead fluorescent tracers.
- Figure S13 Structures of leading fluorescent tracers.

SUPPLEMENTARY METHODS

Reagents

Unless otherwise specified, chemicals were from Sigma. Unmodified compounds were from Sigma, Tocris and Alfa Aesar.

DNA constructs

cDNA for human GPCRs were obtained from Kazusa DNA Research Institute (Chiba, Japan) and subcloned without any native secretion signals into a modified pF5 CMV-neo Flexi vector (Promega) using the Flexi cloning system (Promega). The pF5 vector was modified to enable the generation of GPCR constructs tagged at their N-termini with a HiBiT tag. In addition, constructs included an N-terminal IL6 secretion tag (MNSFSTSAFGPVAFSLGLLLVLPAAFPAP), followed by a VS linker, a HiBiT tag, and a 2 X GSSG linker[1].

Table 1: List of the human GPCRs coding regions used in this study:

Family	Gene	Description
Adenosine	ADORA1	adenosine A1 receptor
	ADORA2A	adenosine A2a receptor
	ADORA2B	adenosine A2b receptor
	ADORA3	adenosine A3 receptor
Adrenoceptors, alpha	ADRA1A	adrenoceptor alpha 1A
	ADRA1B	adrenoceptor alpha 1B
	ADRA1D	adrenoceptor alpha 1D
	ADRA2A	adrenoceptor alpha 2A
	ADRA2B	adrenoceptor alpha 2B
	ADRA2C	adrenoceptor alpha 2C
Adrenoceptors, beta	ADRB1	adrenoceptor beta 1
	ADRB2	adrenoceptor beta 2, surface
	ADRB3	adrenoceptor beta 3

**Family**	**Gene**	**Description**
Angiotensin	AGTR1	angiotensin II receptor, type 1
	AGTR2	angiotensin II receptor, type 2
Vasopressin/Oxytocin	AVPR1A	arginine vasopressin receptor 1A
	AVPR1B	arginine vasopressin receptor 1B
	AVPR2	arginine vasopressin receptor 2
Chemokine	CCR1	chemokine (C-C motif) receptor 1
	CCR2	chemokine (C-C motif) receptor 2
	CCR3	chemokine (C-C motif) receptor 3
	CCR4	chemokine (C-C motif) receptor 4
	CCR5	chemokine (C-C motif) receptor 5
	CCR6	chemokine (C-C motif) receptor 6
	CCR7	chemokine (C-C motif) receptor 7
	CCR8	chemokine (C-C motif) receptor 8
	CCR9	chemokine (C-C motif) receptor 9
	CCR10	chemokine (C-C motif) receptor 10
	CX3CR1	chemokine (C-X3-C motif) receptor 1
	CXCR1	chemokine (C-X-C motif) receptor 1
	CXCR2	chemokine (C-X-C motif) receptor 2
	CXCR3	chemokine (C-X-C motif) receptor 3
	CXCR4	chemokine (C-X-C motif) receptor 4
	CXCR5	chemokine (C-X-C motif) receptor 5
	CXCR6	Chemokine (C-X-C motif)
	ACKR3	chemokine (C-X-C motif) receptor 7/Atypical chemokine receptor 3
	XCR1	chemokine (C motif) receptor 1
Cholecystokinin	CCKAR	cholecystokinin A receptor
	CCKBR	cholecystokinin B receptor
	C3AR1	complement component 3a receptor 1
	C5AR1	complement component 5a receptor 1
	C5AR2	complement component 5a receptor 2
Acetylcholine	CHRM1	cholinergic receptor, muscarinic 1
	CHRM2	cholinergic receptor, muscarinic 2
	CHRM3	cholinergic receptor, muscarinic 3
	CHRM4	cholinergic receptor, muscarinic 4
	CHRM5	cholinergic receptor, muscarinic 5
Dopamine	DRD1	dopamine receptor D1
	DRD2	dopamine receptor D2
	DRD3	dopamine receptor D3
	DRD4	dopamine receptor D4
	DRD5	dopamine receptor D5

Family	Gene	Description
Endothelin	EDNRA	endothelin receptor type A
	EDNRB	endothelin receptor type B
Frizzled	FZD1	frizzled family receptor 1
	FZD2	frizzled family receptor 2
	FZD4	frizzled family receptor 4
	FZD5	frizzled family receptor 5
	FZD6	frizzled family receptor 6
	FZD7	frizzled family receptor 7
	FZD8	frizzled family receptor 8
	FZD9	frizzled family receptor 9
	FZD10	frizzled family receptor 10
	SMO	smoothened, frizzled family receptor
GABA	GABBR1	gamma-aminobutyric acid (GABA) B receptor,1
	GABBR2	gamma-aminobutyric acid (GABA) B receptor,2
Histamine	HRH1	histamine receptor H1
	HRH2	histamine receptor H2
	HRH3	histamine receptor H3
	HRH4	histamine receptor H4
5-Hydroxytryptamine	HTR1A	5-hydroxytryptamine (serotonin) receptor 1A
	HTR1B	5-hydroxytryptamine (serotonin) receptor 1B
	HTR1D	5-hydroxytryptamine (serotonin) receptor 1D
	HTR1E	5-hydroxytryptamine (serotonin) receptor 1E
	HTR1F	5-hydroxytryptamine (serotonin) receptor 1F
	HTR2A	5-hydroxytryptamine (serotonin) receptor 2A
	HTR2B	5-hydroxytryptamine (serotonin) receptor 2B
	HTR2C	5-hydroxytryptamine (serotonin) receptor 2C
	HTR5A	5-hydroxytryptamine (serotonin) receptor 5A
	HTR4	5-hydroxytryptamine (serotonin) receptor 4
	HTR6	5-hydroxytryptamine (serotonin) receptor 6
	HTR7	5-hydroxytryptamine (serotonin) receptor 7
Orphans	LGR4	leucine-rich repeat containing GPCR4
	LGR5	leucine-rich repeat containing GPCR5
	LGR6	leucine-rich repeat containing GPCR6
Leukotriene	LTB4R	leukotriene B4 receptor
	LTB4R2	leukotriene B4 receptor 2
	OXER1	oxoeicosanoid (OXE) receptor 1
Opioid	OPRD1	opioid receptor, delta 1
	OPRK1	opioid receptor, kappa 1
	OPRM1	opioid receptor, mu 1

Family	Gene	Description
Nociceptin	OPRL1	opiate receptor-like 1
Platelet-activating	PTAFR	platelet-activating factor receptor
P2Y	P2RY1	purinergic receptor P2Y, G-protein coupled, 1
	P2RY2	purinergic receptor P2Y, G-protein coupled, 2
	P2RY4	pyrimidinergic receptor P2Y, G-protein coupled, 4
	P2RY6	pyrimidinergic receptor P2Y, G-protein coupled, 6
	P2RY8	purinergic receptor P2Y, G-protein coupled, 8
	P2RY10	purinergic receptor P2Y, G-protein coupled, 10
	P2RY11	purinergic receptor P2Y, G-protein coupled, 11
	P2RY12	purinergic receptor P2Y, G-protein coupled, 12
	P2RY13	purinergic receptor P2Y, G-protein coupled, 13
	P2RY14	purinergic receptor P2Y, G-protein coupled, 14
VIP and PACAP	VIPR1	vasoactive intestinal peptide receptor 1
	VIPR2	vasoactive intestinal peptide receptor 2
Bombesin	BRS3	bombesin-like receptor 3
	GRPR	gastrin-releasing peptide receptor
Bradykinin	BDKRB1	bradykinin receptor B1
	BDKRB2	bradykinin receptor B2
Formylpeptide	FPR1	formyl peptide receptor 1
	FPR2	formyl peptide receptor 2
	FPR3	formyl peptide receptor 3
Galanin	GALR1	galanin receptor 1
	GALR2	galanin receptor 2
	GALR3	galanin receptor 3
Ghrelin	GHSR	growth hormone secretagogue receptor
Glycoprotein	FSHR	follicle stimulating hormone receptor
	TSHR	thyroid stimulating hormone receptor
Gonadotrophin	GNRHR	gonadotropin-releasing hormone receptor
Orexin	HCRTR1	hypocretin (orexin) receptor 1
	HCRTR2	hypocretin (orexin) receptor 2
Melanin-conc	MCHR1	melanin-concentrating hormone receptor 1
	MCHR2	melanin-concentrating hormone receptor 2
Motilin	MLNR	motilin receptor
Neuromedin	NMUR1	neuromedin U receptor 1
	NMUR2	neuromedin U receptor 2
Neuropeptide Y	NPY1R	neuropeptide Y receptor Y1
	NPY2R	neuropeptide Y receptor Y2
	NPY4R	neuropeptide Y receptor Y4
	NPY5R	neuropeptide Y receptor Y5

Family	Gene	Description
Neuropeptide S	NPSR1	neuropeptide S receptor 1
Proteinase-activated receptor	F2R	coagulation factor II (thrombin) receptor
	F2RL1	coagulation factor II (thrombin) receptor-like 1
	F2RL2	coagulation factor II (thrombin) receptor-like 2
	F2RL3	coagulation factor II (thrombin) receptor-like 3
Relaxin	RXFP1	Relaxin /insulin-like family peptide receptor 1
	RXFP2	Relaxin /insulin-like family peptide receptor 2
	RXFP3	Relaxin /insulin-like family peptide receptor 3
	RXFP4	Relaxin /insulin-like family peptide receptor 4
Somatostatin	SSTR1	somatostatin receptor 1
	SSTR2	somatostatin receptor 2
	SSTR3	somatostatin receptor 3
	SSTR4	somatostatin receptor 4
	SSTR5	somatostatin receptor 5
Tachykinin	TACR1	tachykinin receptor 1
	TACR2	tachykinin receptor 2
	TACR3	tachykinin receptor 3
Oxytocin	OXTR	oxytocin receptor
melanocortin	MC2R	melanocortin 2 receptor
	MC3R	melanocortin 3 receptor
	MC4R	melanocortin 4 receptor
	MC5R	melanocortin 5 receptor
Neuropeptide W/B	NPBWR1	neuropeptides B/W receptor 1
	NPBWR2	neuropeptides B/W receptor 2
Neuropeptide FF/AF	NPFFR1	neuropeptide FF receptor 1
	NPFFR2	neuropeptide FF receptor 2
Thromboxane	TBXA2R	thromboxane A2 receptor
Prostaglandin	PTGDR	prostaglandin D2 receptor (DP)
	PTGDR2	prostaglandin D2 receptor 2
	PTGER1	prostaglandin E receptor 1 (subtype EP1),
	PTGER2	prostaglandin E receptor 2 (subtype EP2),
	PTGER3	prostaglandin E receptor 3 (subtype EP3)
	PTGER4	prostaglandin E receptor 4 (subtype EP4)
	PTGFR	prostaglandin F receptor (FP)
	PTGIR	prostaglandin I2 (prostacyclin) receptor (IP)
Melatonin	MTNR1A	melatonin receptor 1A
	MTNR1B	melatonin receptor 1B
	GPR50	G protein-coupled receptor 50
Cannabinoid	CNR1	cannabinoid receptor 1 (brain)
	CNR2	cannabinoid receptor 2 (macrophage)

Family	Gene	Description
Metabotropic glutamate	GRM1	glutamate receptor, metabotropic 1
	GRM2	glutamate receptor, metabotropic 2
	GRM3	glutamate receptor, metabotropic 3
	GRM4	glutamate receptor, metabotropic 4
	GRM5	glutamate receptor, metabotropic 5
	GRM7	glutamate receptor, metabotropic 7
	GRM8	glutamate receptor, metabotropic 8
Glucagon	ADCYAP1R1	adenylate cyclase activating polypeptide 1 (pituitary) receptor type I
	GCGR	glucagon receptor
Lutropin-choriogonadotropin	LHCGR	luteinizing hormone/choriogonadotropin receptor
Cysteinyl leukotriene	CYSLTR1	cysteinyl leukotriene receptor 1
	CYSLTR2	cysteinyl leukotriene receptor 2

Cell culture and transfection

HEK293 cells were obtained from American Type Culture Collection (ATCC) and cultured at 37 ºC/5% CO_2 in Dulbecco's Modified Eagle Medium (DMEM) (Gibco) supplemented with 10% fetal bovine serum (Seradigm) and 100 units/mL of penicillin-streptomycin (Gibco). Transfections were carried out using Viafect (Promega) at a 1:3 DNA/transfection reagent ratio. Generally, DNA encoding HiBiT-GPCR fusion was diluted 1:100 in promoterless carrier DNA. Cells suspended in Opti-MEM (without phenol red) supplemented with 2% FBS and 100 units/mL penicillin–streptomycin (Gibco) at a final concentration of 220,000 cells/mL, were combined with DNA–transfection reagent complexes, seeded in white 96-well plates at 18,000 cells / well, and grown for 18-20 h at 37 °C/5% CO_2.

NanoBRET saturation ligand-engagement assay

Saturation tracer binding assays were carried out according to the previously published protocol [2]. Briefly, cells were treated with a serial dilution of a tracer alone or

in the presence of 30 μM competing unmodified compound for 90 min at room temperature. Following treatment with HiBiT detection reagent (comprising LgBiT and furimazine live cells substrate (Promega)) plates were mixed for 15 min to allow HiBiT/LgBiT complementation. Filtered luminescence was then measured using GloMax Discover microplate reader (Promega) equipped with a 450-nm (8-nm band pass) filter (donor) and a 600-nm long pass filter (acceptor). BRET was calculated by dividing the acceptor emission by the donor emission. The values were background corrected by subtracting the BRET values from parallel samples treated with excess competing compound. To determine the apparent affinity for the tracer (EC_{50}), data was plotted in GraphPad Prism software using the log(agonist) vs. response–variable slope fitting.

SUPPLEMENTARY FIGURES AND TABLES

Table S1. GPCR families in the GLASS dataset as numbered in Figure 1.

Number	GPCR family	Number	GPCR family
1	5-Hydroxytryptamine	40	Calcitonin
2	Adenosine	41	Hydroxycarboxylic acid
3	Dopamine	42	Bombesin
4	Opioid	43	Oxytocin
5	Cannabinoid	44	Leukotriene
6	Chemokine	45	Urotensin
7	Acetylcholine	46	Frizzled
8	Histamine	47	Platelet-activating
9	Melanocortin	48	Formylpeptide
10	Prostaglandin	49	VIP and PACAP
11	Metabotropic glutamate	50	Bile acid
12	AdrenalineBeta	51	Neurotensin
13	AdrenalineAlpha	52	Postacyclin
14	Glucagon	53	GPR18/55/119
15	Lysophospholipid	54	Calcium-sensing
16	Orexin	55	Neuropeptide FF/AF
17	Melanin-conc	56	Neuropeptide S
18	Somatostatin	57	Glycoprotein
19	Parathyroid	58	Cysteinyl leukotriene
20	Tachykinin	59	Kisspeptin
21	Cholecystokinin	60	Motilin
22	P2Y	61	Apelin
23	Endothelin	62	Neuropeptide W/B
24	Ghrelin	63	Galanin
25	Vasopressin	64	Prokineticin
26	Melatonin	65	Trace amine
27	Proteinase-activated receptor	66	Peptide P518
28	Free fatty acid	67	Lutropin-choriogonadotropin
29	Neuropeptide Y	68	Prolactin
30	Gonadotrophin	69	Estrogen
31	Bradykinin	70	Succinate
32	Orphan	71	Thyrotropin
33	Corticotropin	72	GABA
34	Nociceptin	73	Olfactory
35	Thromboxane	74	Taste 2
36	Lipid Amine	75	Taste 3
37	Relaxin	76	Taste 4
38	Angiotensin	77	Psychosine
39	Neuromedin		

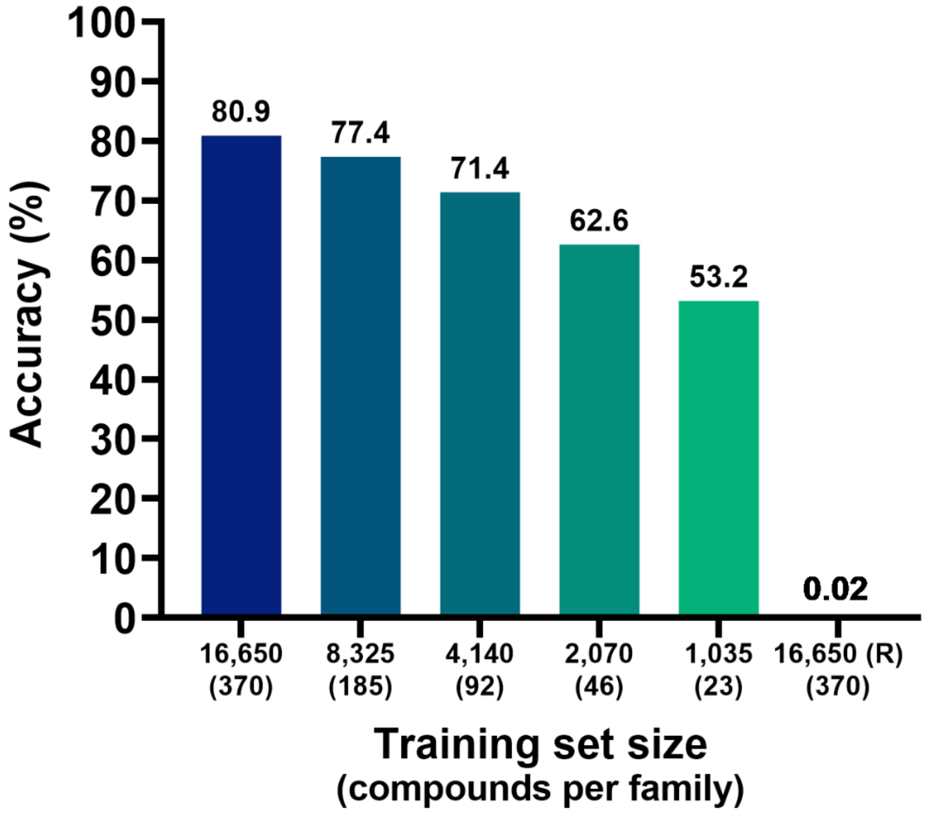

Figure S1. Performance of machine learning models using reduced training set sizes. Models trained with different numbers of compounds per family shows a decrease in classification accuracy on compounds in the test set as the size of the training set is reduced. Randomization (R) of the training set labels resulted in a low accuracy expected for random classification, indicating classification accuracy is dependent on the correct annotation of molecular fingerprints with their target GPCR families.

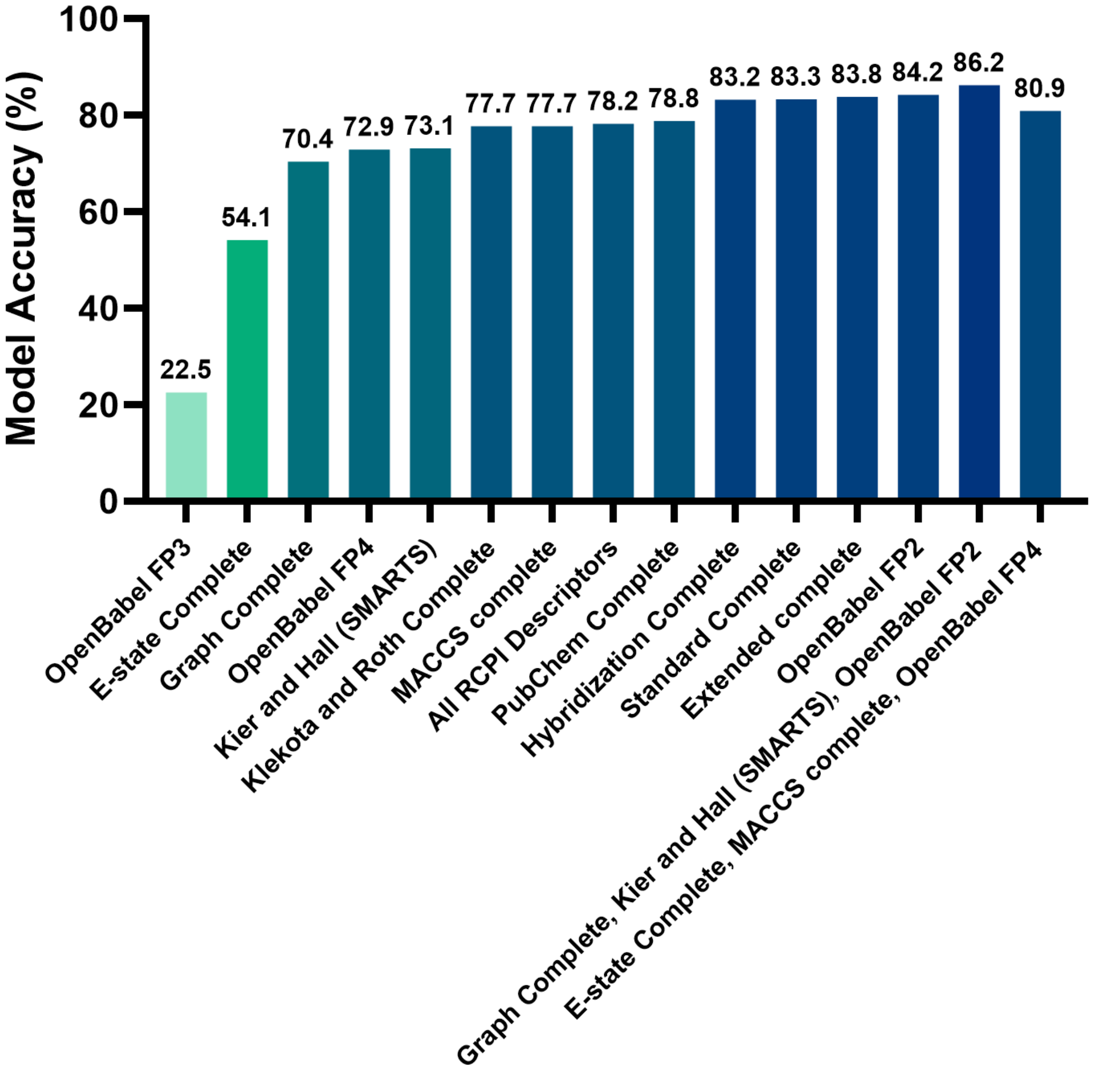

Figure S2. Classification accuracy for machine learning models utilizing different molecular fingerprint representations of the training set. Classification accuracy on compounds in the test set is shown for models trained using the indicated molecular fingerprint representations of the training set. Certain combinations of fingerprint schemes could improve model accuracy up to 86.2%. The model providing the best correlation with empirical NanoBRET screening data across all the tested GPCR families had a lower test set accuracy of 80.9% and utilized a combination of E-state Complete, MACCS Complete, and OpenBabel FP4 fingerprints.

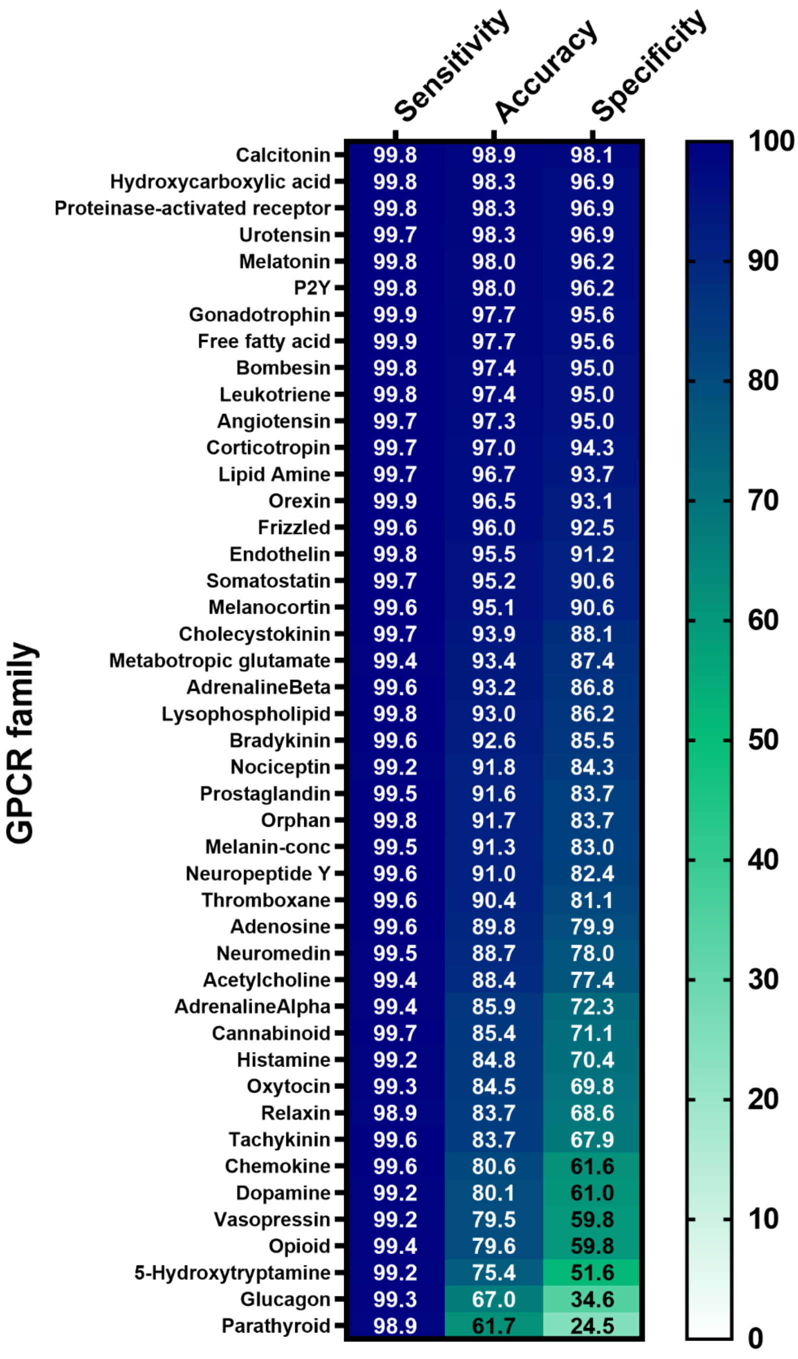

Figure S3. Performance metrics for machine learning model on a per-family basis. The accuracy, sensitivity, and specificity metrics of model predictions for compounds in the test set are shown as a percentage for each GPCR family and are colored as a heatmap.

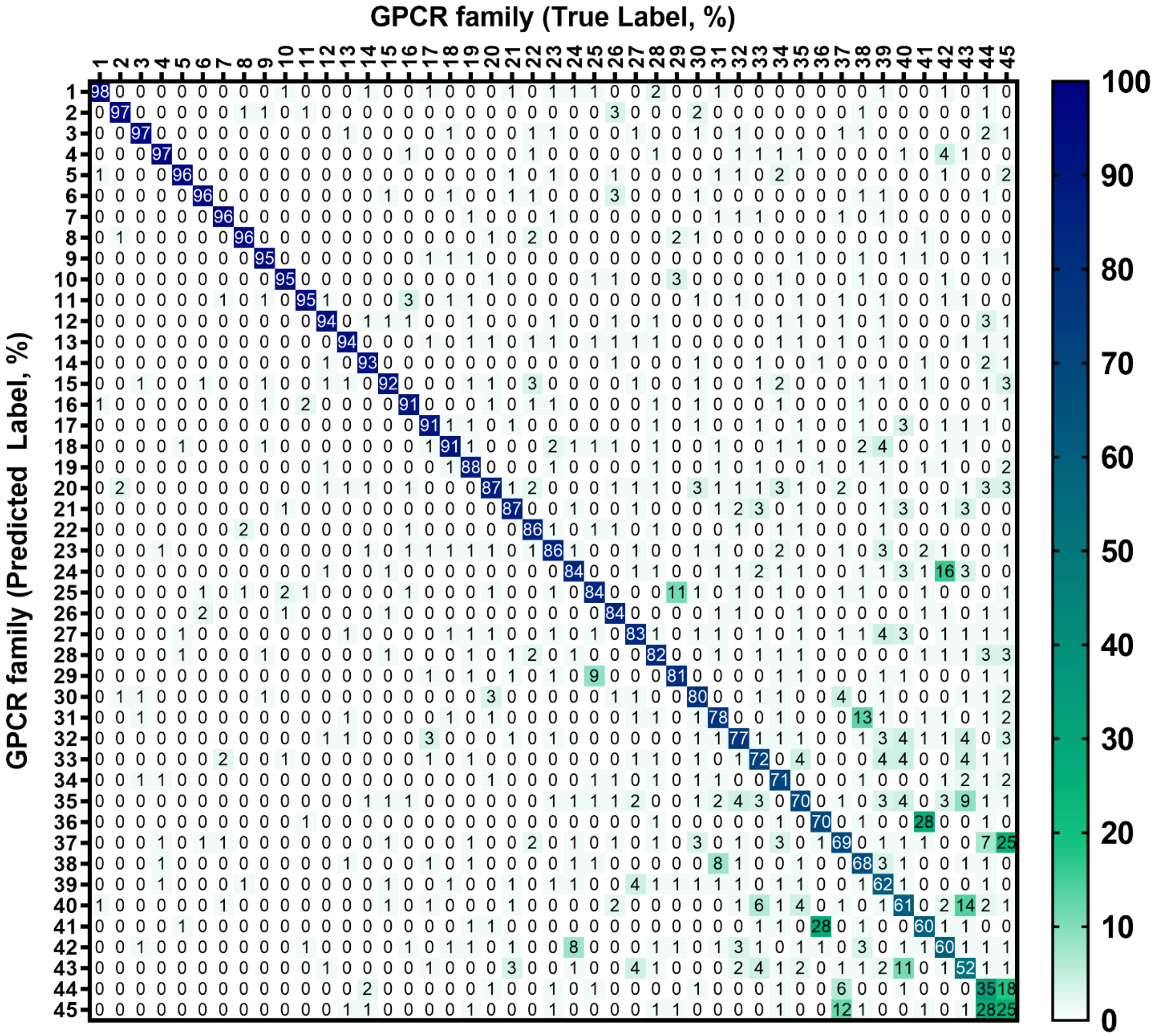

Figure S4. Confusion matrix on a per-family basis of machine learning model classification predictions. The percentage of compounds in the test set classified by the model into their predicted GPCR target family versus their true (annotated) GPCR target family. The value of each position is colored according to the legend on the right.

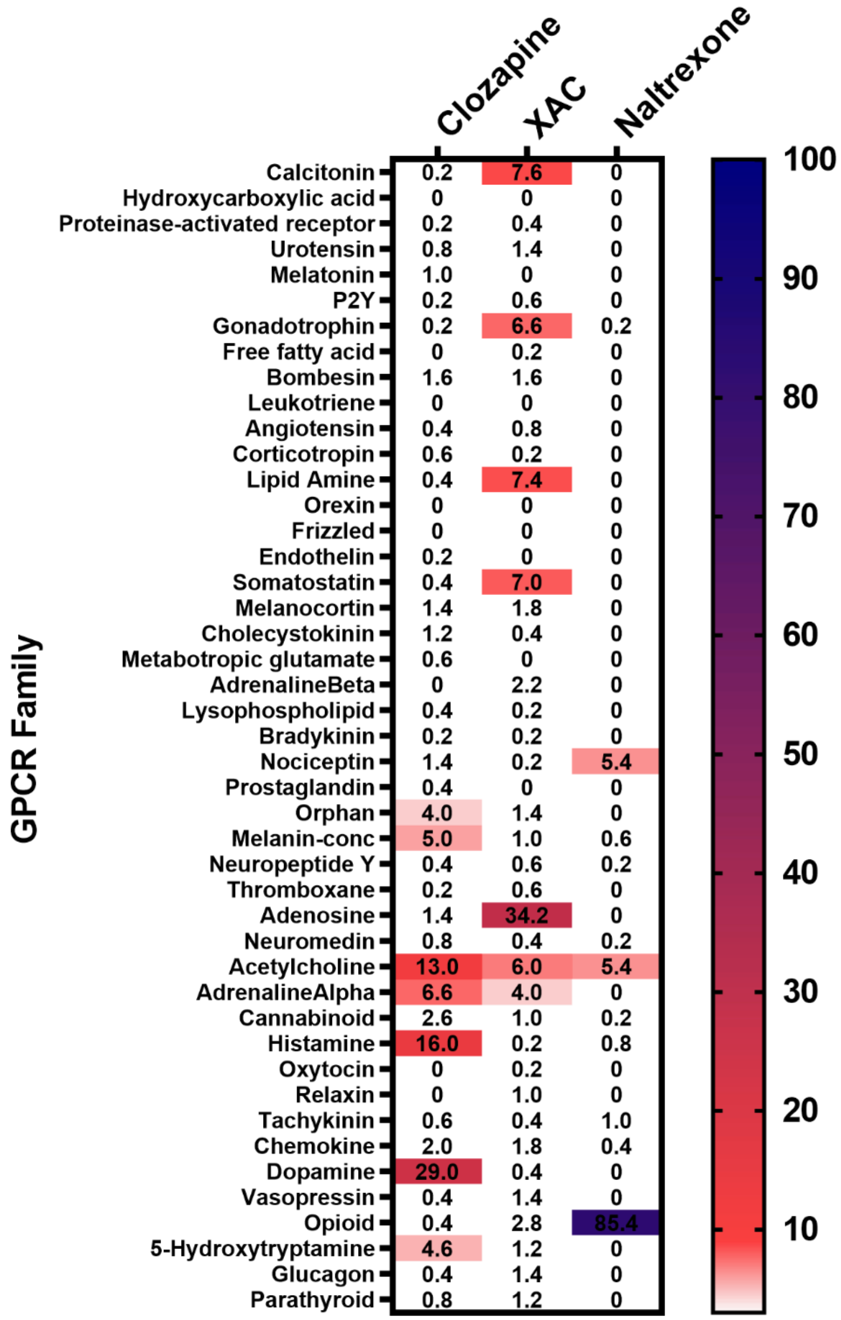

GPCR Family	Clozapine	XAC	Naltrexone
Calcitonin	0.2	7.6	0
Hydroxycarboxylic acid	0	0	0
Proteinase-activated receptor	0.2	0.4	0
Urotensin	0.8	1.4	0
Melatonin	1.0	0	0
P2Y	0.2	0.6	0
Gonadotrophin	0.2	6.6	0.2
Free fatty acid	0	0.2	0
Bombesin	1.6	1.6	0
Leukotriene	0	0	0
Angiotensin	0.4	0.8	0
Corticotropin	0.6	0.2	0
Lipid Amine	0.4	7.4	0
Orexin	0	0	0
Frizzled	0	0	0
Endothelin	0.2	0	0
Somatostatin	0.4	7.0	0
Melanocortin	1.4	1.8	0
Cholecystokinin	1.2	0.4	0
Metabotropic glutamate	0.6	0	0
AdrenalineBeta	0	2.2	0
Lysophospholipid	0.4	0.2	0
Bradykinin	0.2	0.2	0
Nociceptin	1.4	0.2	5.4
Prostaglandin	0.4	0	0
Orphan	4.0	1.4	0
Melanin-conc	5.0	1.0	0.6
Neuropeptide Y	0.4	0.6	0.2
Thromboxane	0.2	0.6	0
Adenosine	1.4	34.2	0
Neuromedin	0.8	0.4	0.2
Acetylcholine	13.0	6.0	5.4
AdrenalineAlpha	6.6	4.0	0
Cannabinoid	2.6	1.0	0.2
Histamine	16.0	0.2	0.8
Oxytocin	0	0.2	0
Relaxin	0	1.0	0
Tachykinin	0.6	0.4	1.0
Chemokine	2.0	1.8	0.4
Dopamine	29.0	0.4	0
Vasopressin	0.4	1.4	0
Opioid	0.4	2.8	85.4
5-Hydroxytryptamine	4.6	1.2	0
Glucagon	0.4	1.4	0
Parathyroid	0.8	1.2	0

Figure S5. Machine learning model predictions of interactions for three unmodified scaffolds. Example of model predictions for three unmodified molecular scaffolds used as the basis for fluorescent NanoBRET tracers. Predictions are colored as a heatmap specifying the classification probability for each GPCR family as percentage.

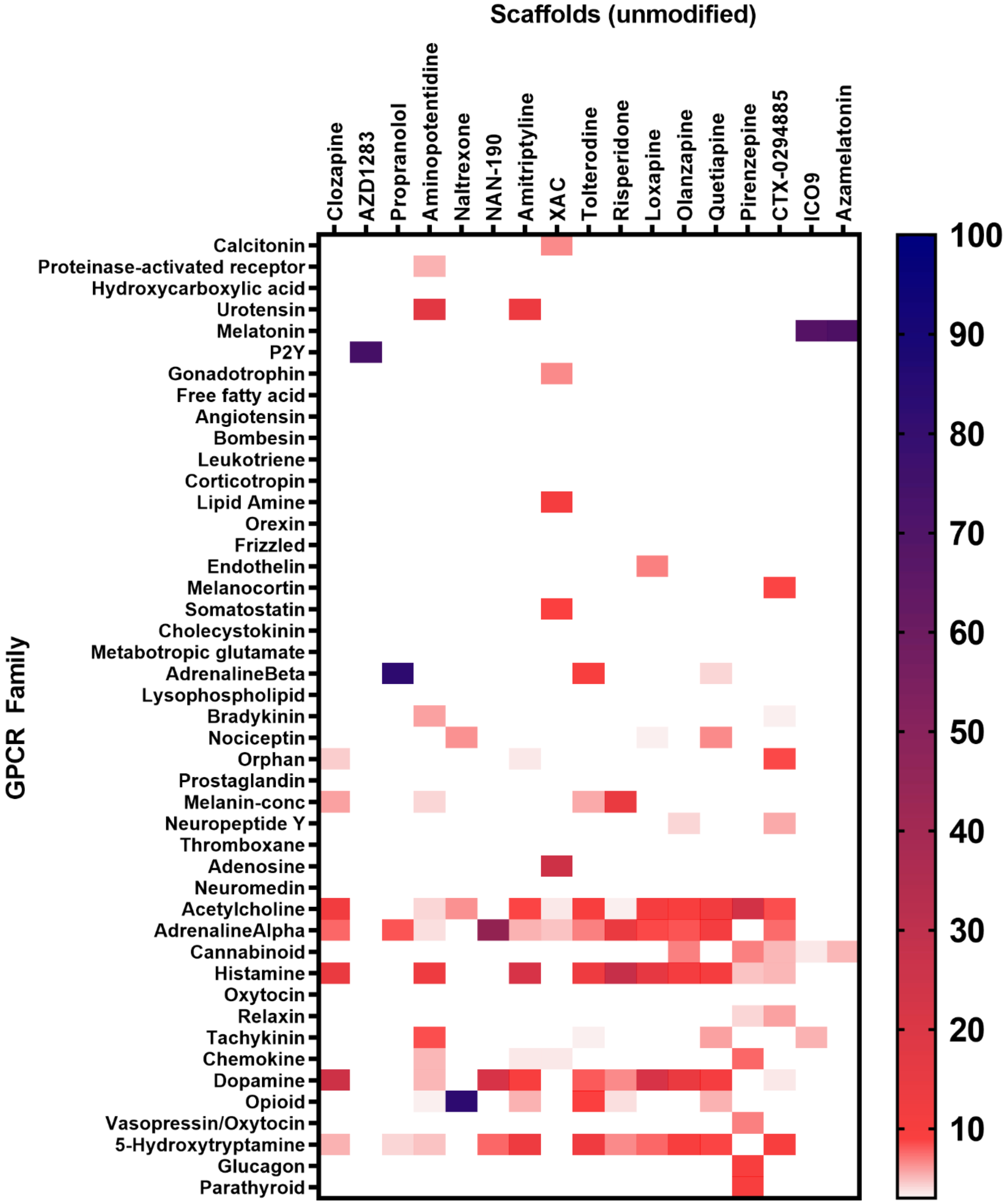

Figure S6. Machine learning model predictions for all unmodified scaffolds. The predicted probability of interactions for each unmodified scaffold across GPCR target families. The percent probability is represented as a heatmap for all tested combinations.

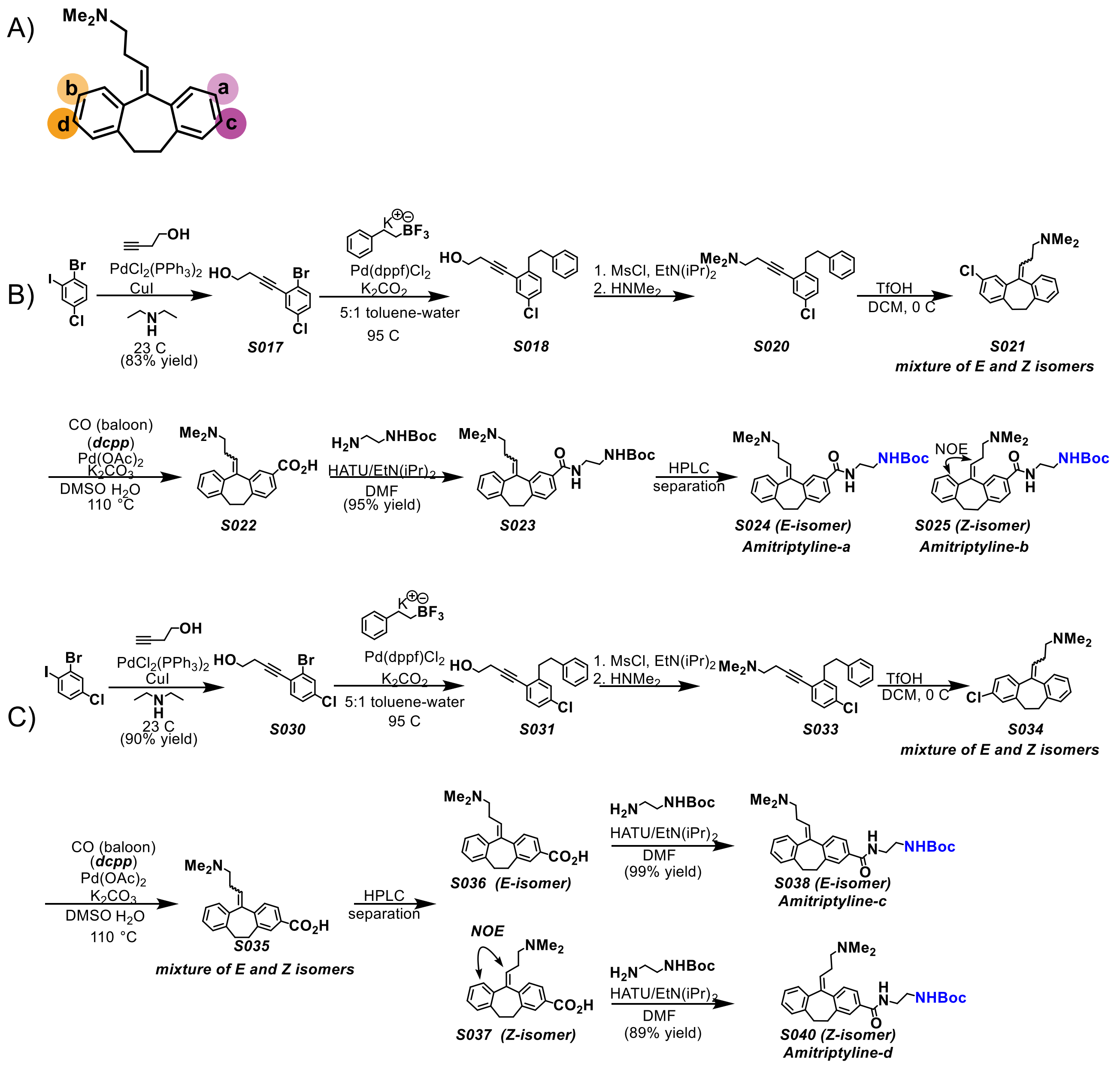

Figure S7. Synthesis of modifiable Amitriptyline analogues. (A) Amitriptyline with modifiable positions marked. Applying a published approach[3] for (B) Synthesis of Amitriptyline-a and Amitriptyline-b and (C) Synthesis of Amitriptyline-c and Amitriptyline-d.

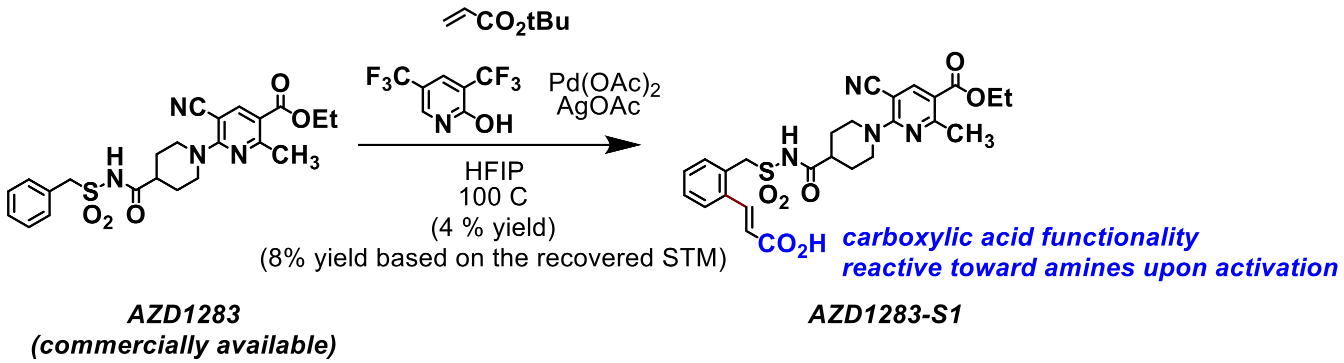

Figure S8. Synthesis of modifiable AZD1283 analogue. Late-stage Yu alkenylation conditions[4] were applied to commercially available AZD1283. Tert-butyl ester came off under reaction conditions. Sulfonamide functionality directed alkenylation to the ortho-position of phenyl ring. Significant amounts of bis-ortho-alkenylation byproduct were isolated.

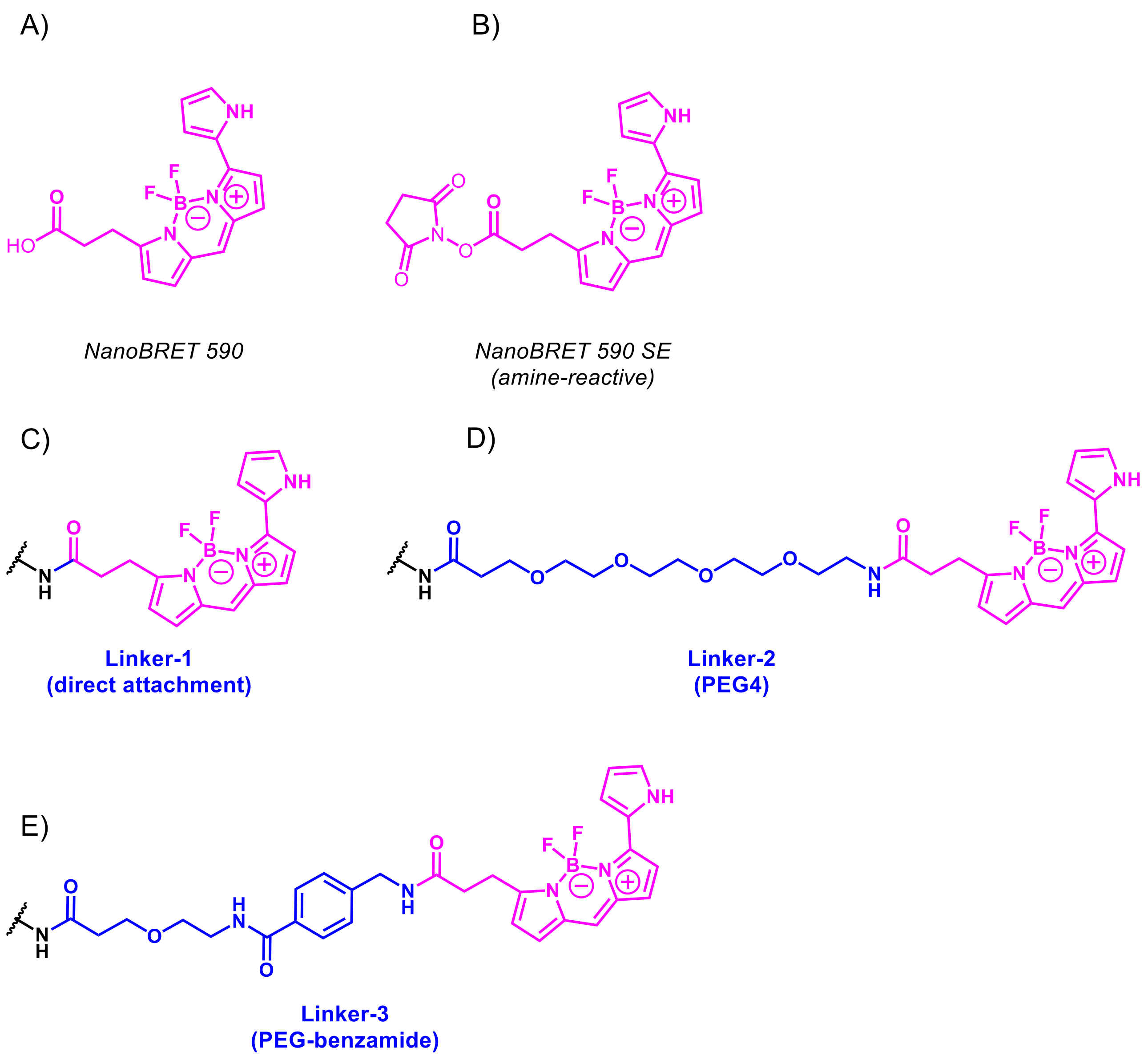

Figure S9. Structures of NanoBRET 590 and linkers connecting it to selected scaffolds. (A) NanoBRET 590, (B) NanoBRET 590 SE, (C) direct attachment, (D) PEG4 linker and (E) PEG-benzamide linker.

Table S2. Fluorescent tracers generated and characterized in this study.

Scaffold	Modification site	Linker	Tracer
Clozapine	a	Linker-2(PEG4)	Clozapine-a-2-NB590
	b	Linker-2(PEG4)	Clozapine-b-2-NB590
	c	Linker-2(PEG4)	Clozapine-c-2-NB590
AZD1283		Linker-1(direct attachment)	AZD1283-1-NB590
Propranolol		Linker-3(PEG-benzamide)	Propranolol-3-NB590
Aminopotentidine		Linker-2(PEG4)	Aminopotentidine-2-NB590
Naltrexone		Linker-3(PEG-benzamide)	Naltrexone-3-NB590
NAN-190		Linker-2(PEG4)	NAN-190-2-NB590
		Linker-3(PEG-benzamide)	NAN-190-3-NB590
Amitriptyline	a	Linker-2(PEG4)	Amitriptyline-a-2-NB590
	b	Linker-2(PEG4)	Amitriptyline-b-2-NB590
	c	Linker-2(PEG4)	Amitriptyline-c-2-NB590
	d	Linker-2(PEG4)	Amitriptyline-d-2-NB590
	e	Linker-2(PEG4)	Amitriptyline-e-2-NB590
XAC		Linker-3(PEG-benzamide)	XAC-3-NB590
Tolterodine		benzyl	Tolterodine-BZ-NB590
		Linker-2(PEG4)	Tolterodine-2-NB590
Risperidone		Linker-2(PEG4)	Risperidone-2-NB590
Loxapine	a	Linker-2(PEG4)	Loxapine-a-2-NB590
Olanzapine	a	Linker-2(PEG4)	Olanzapine-a-2-NB590
Quetiapine	a	Linker-2(PEG4)	Quetiapine-a-2-NB590
Pirenzepine		Linker-2(PEG4)	Pirenzepine-2-NB590
CTX-0294885		Linker-2(PEG4)	CTX-0294885-2-NB590
ICO9 (Azamelatonin)		Linker-2(PEG4)	ICO9-2-NB590
Azamelatonin		Linker-1(direct attachment)	Azamelatonin-1-NB590

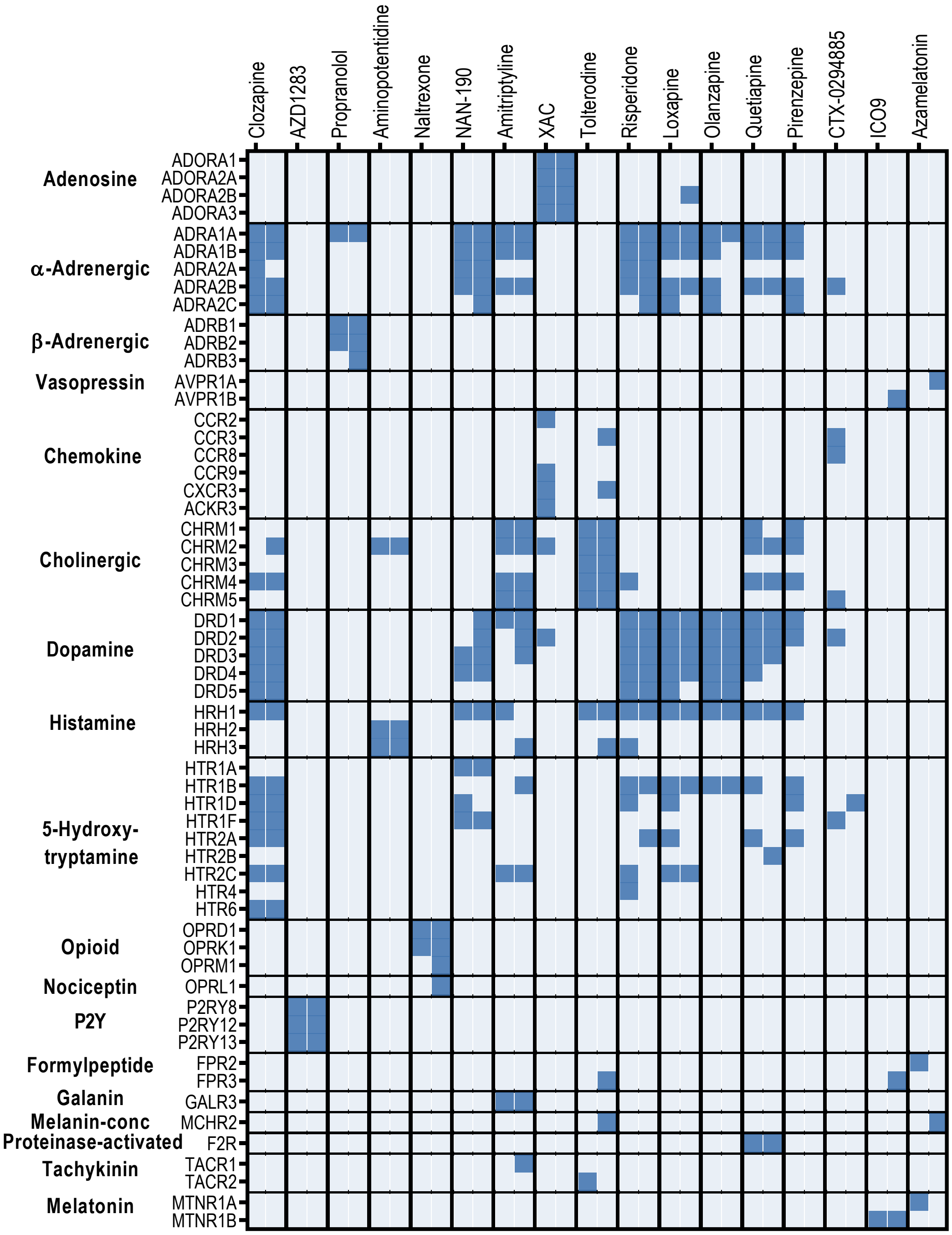

Figure S10. Binding profile of the leading tracer for each selected scaffold. Specific interactions (≥1.5-fold response) for leading tracer candidates that were revealed in a NanoBRET screen across 184 HiBiT-GPCRs from 51 families (Compiled screening results are included in the Supplementary Excel spreadsheet).

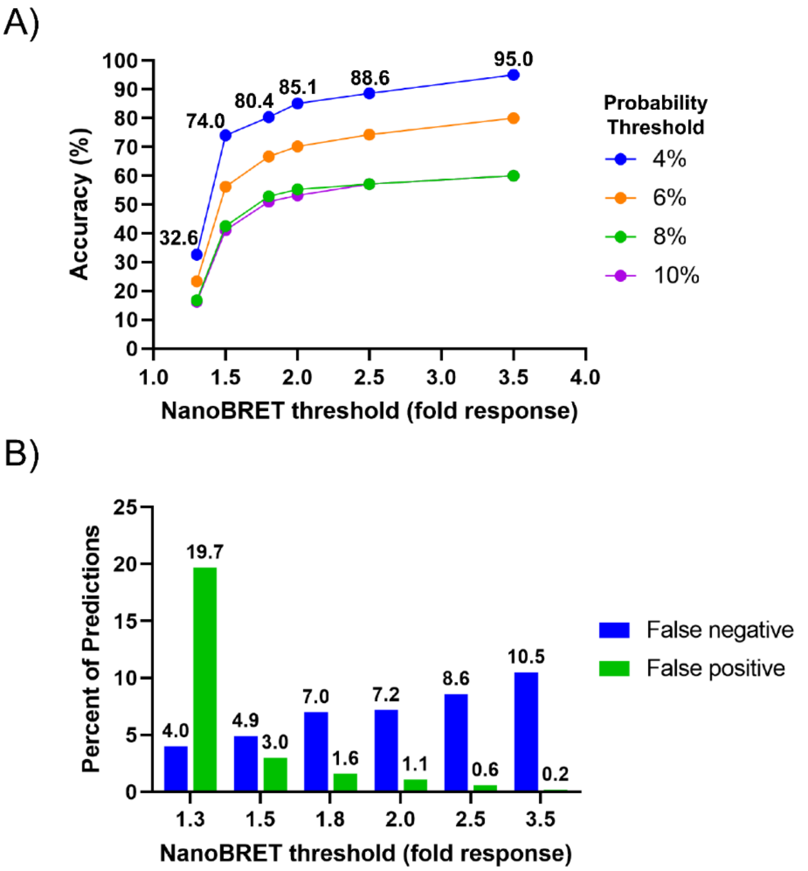

Figure S11. Comparison of thresholds for machine learning model predictions and NanoBRET screens. (A) Accuracy of machine learning model predictions determined at different classification probability thresholds for positive interactions. At higher probability thresholds, stringency on model prediction is increased and less predictions are classified as positive. Likewise, as the NanoBRET fold response threshold is increased, less interactions are considered specific. The highest correlation between these two trends was at lower class probability thresholds. (B) False negative (%) and false positive (%) predictions for a machine learning model using a 4% threshold compared to interactions confirmed by NanoBRET at increasing fold response thresholds. A NanoBRET threshold of ≥1.5-fold response provided the best balance between model accuracy and a low percentage of false predictions.

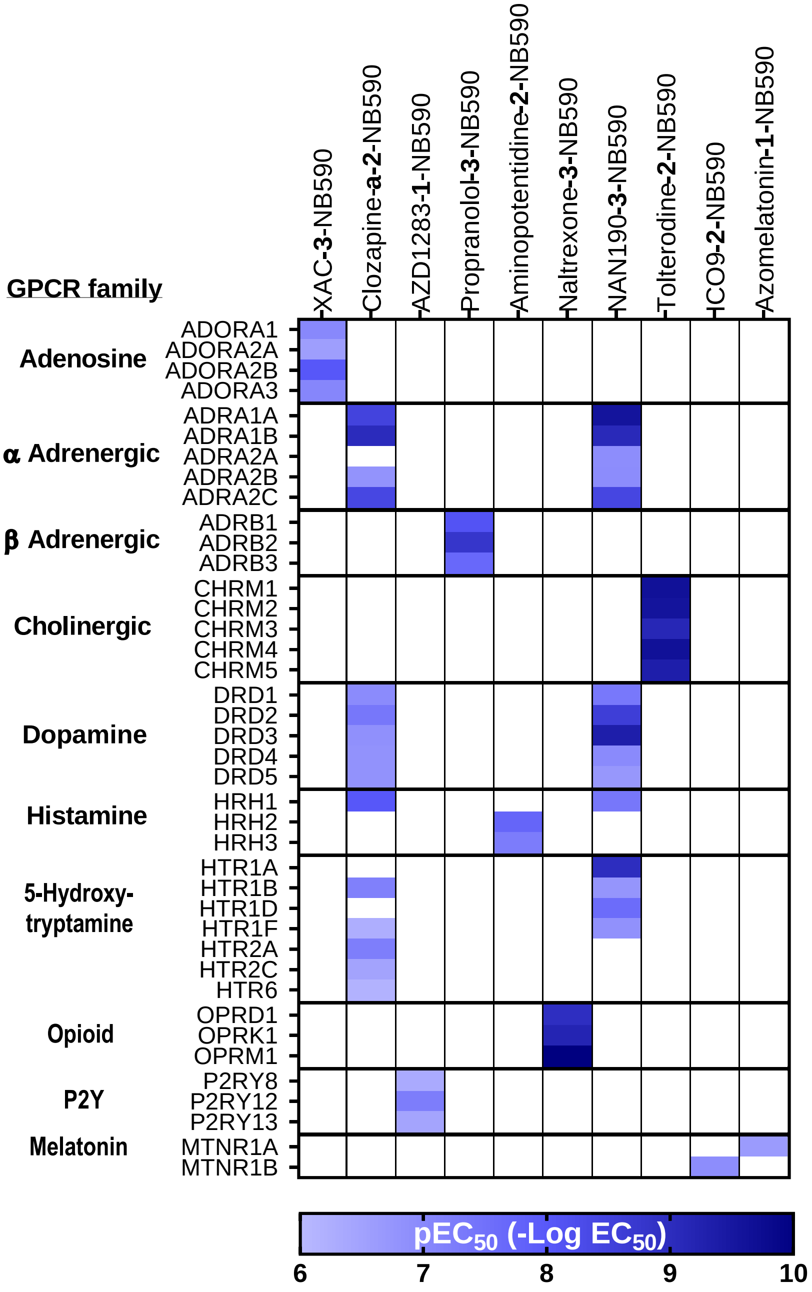

Figure S12. Summary of GPCRs NanoBRET engagement assays and their lead fluorescent tracers. Summary of NanoBRET GPCRs engagement assays alongside the leading tracers for each assay. The pEC_{50} (-Log EC_{50}) values are presented as a heatmap. Structures of leading tracers are shown in Supplementary Figure S13.

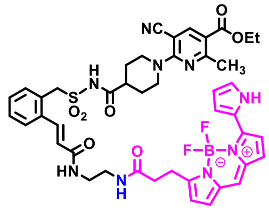

AZD1283-1-NB590

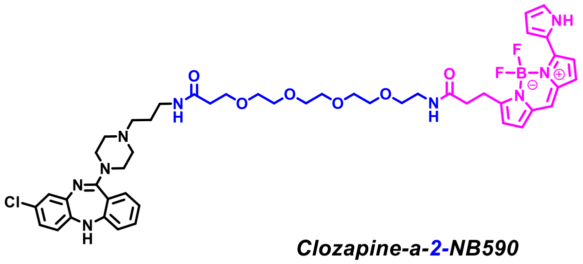

Clozapine-a-2-NB590

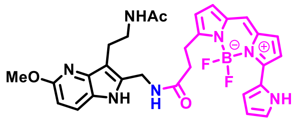

Azomelatonin-1-NB590

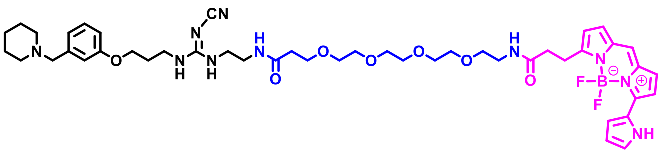

Aminopotentidin-2-NB590

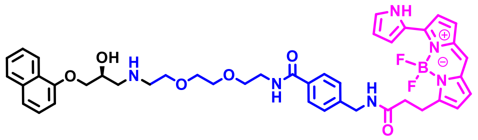

Propranolol-3-NB590

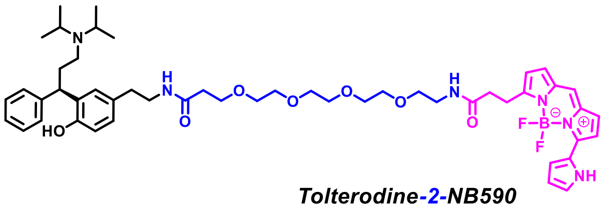

Tolterodine-2-NB590

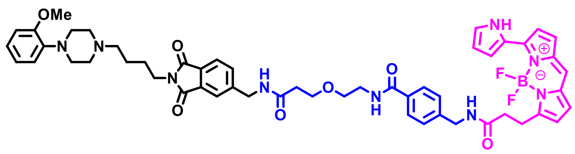

NAN-190-3-NB590

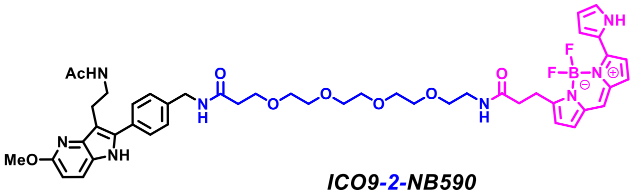

ICO9-2-NB590

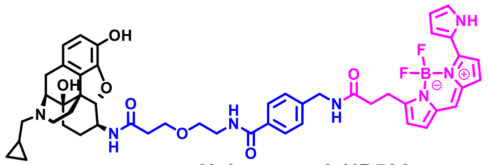

Naltrexone-3-NB590

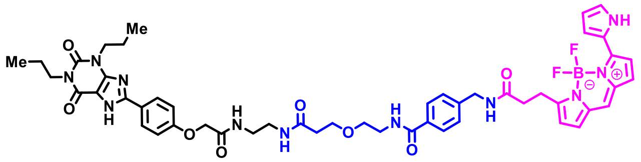

XAC-3-NB590

Figure S13. Structures of leading fluorescent tracers. Structures of leading tracers associated with the GPCR engagement assays described in Supplementary Figure S12. Each structure highlights the conjugation site and preferred linker connecting it to NanoBRET 590.

REFERENCES FOR SUPPLEMENTARY INFORMATION PART 1

1. Boursier, M.E., et al., *The luminescent HiBiT peptide enables selective quantitation of G protein-coupled receptor ligand engagement and internalization in living cells.* J Biol Chem, 2020. **295**(15): p. 5124-5135.
2. Boursier, M.E., et al., *Equilibrium and Kinetic Measurements of Ligand Binding to HiBiT-tagged GPCRs on the Surface of Living Cells.* Bio Protoc, 2020. **10**(24): p. e3861.
3. Otani, T., et al., *Construction of dibenzo-fused seven- to nine-membered carbocycles via Bronsted acid-promoted intramolecular Friedel-Crafts-type alkenylation.* Chem Commun (Camb), 2015. **51**(37): p. 7895-8.
4. Wang, P., et al., *Ligand-accelerated non-directed C-H functionalization of arenes.* Nature, 2017. **551**(7681): p. 489-493.

SUPPLEMENTARY INFORMATION PART 2

(Synthetic Procedures and Compound Characterization)

General…………………………………………………………………………………… 27

Synthesis of Clozapine tracers………………………………..………………………… 28

Clozapine-a tracer………….……………….……………………………………….… 28

Clozapine-b tracer………….……………….………………………………………. 30

Clozapine-c tracer………….……………….………………………………………… 33

Synthesis of Amitriptyline tracers……………...……………………………………… 37

Amitriptyline-a and Amitriptyline-b tracers…………………………………… 37

Amitriptyline-c and Amitriptyline-d tracers…………………………………… 43

Amitriptyline-e tracer……………………………………………………………. 50

Synthesis of AZD1283 tracer ………………………………………………………… 51

Synthesis of Azamelatonin fluorescent tracers……………………………………… 53

Azamelatonin-1 tracer (N-attachment).………………………………………… 53

Azamelatonin-2 ICOA9 tracer...……………………………………………………. 54

Synthesis of Propranolol tracer………………………………………………………. 55

Synthesis of Tolterodine tracers……………………………………………………… 56

Synthesis of NAN190 tracers…………………………………………………………. 58

Synthesis of Naltrexone tracer...……………………………………………………… 60

Synthesis of XAC tracer……………………………………………………………..... 61

Synthesis of Loxapine tracer…………………………………………………………. 63

Synthesis of Olanzapine tracer………………………………………………………. 65

Synthesis of Quetiapine tracer……………………………………………………… 67

Synthesis of Risperidone tracer………………………………………………………. 69

Synthesis of Aminopotentidine tracer……………………………………………… 71

Synthesis of Pirenzepine tracer………………………………………………………. 73

CTX-0294885 Tracer…………………………………………………………………. 75

References…...……………………………………………………………………… 75

General. Reactions were performed using commercially obtained solvents. Unless otherwise stated, all commercially obtained reagents were used as received. Reactions were monitored by thin-layer chromatography (TLC) using EMD/Merck silica gel 60 F254 pre-coated plates (0.25 mm). Flash column chromatography was performed using pre-packaged RediSep®Rf columns on a CombiFlash Rf system (Teledyne ISCO Inc.). Microwave experiments were performed using a CEM Discover SP® microwave reactor. ^{1}H and ^{13}C NMR spectra were recorded on a Buker Avance II 400 (at 400 MHz and 100 MHz respectively) and are reported relative to internal C***H***Cl_3 (^{1}H, δ = 7.26), DMSO-d_5 (^{1}H, δ = 2.50), CD_2***H***OD (^{1}H, δ = 3.31) and $CDCl_3$ (^{13}C, δ = 77.0), DMSO-d_6 (^{13}C, δ = 39.5), CD_3OD (^{13}C, δ = 49.0). Data for ^{1}H NMR spectra are reported as follows: chemical shift (δ ppm) (multiplicity, coupling constant (Hz), integration). Multiplicity and qualifier abbreviations are as follows: s = singlet, d = doublet, t = triplet, q = quartet, m = multiplet, br = broad, app = apparent. High resolution mass spectra were obtained on AB Sciex TripleTOF 5600+. Preparative HPLC was performed with Waters 2535 Quaternary Gradient Module utilizing XBridge PREP C18 Column 5 µm (30 mm X 250 mm). Analytical HPLC was performed with an Agilent 1100 Series HPLC utilizing Phenomenex Synergi™ 2.5 µm MAX-RP 100 Å columns (4.6 mm x 50 mm).

Chemical abbreviations used:

DCM – dichloromethane; **DIPEA** – 'Hünig's base', N-ethyldiisopropylamine; **DMF** - *N*,*N*-Dimethylformamide; **DMSO** – dimethyl sulfoxide; **EtOAc** – ethyl acetate; **HATU** – *N*-[(Dimethylamino)-1H-1,2,3-triazolo-[4,5-b]pyridin-1-ylmethylene]-N-methylmethanaminium hexafluorophosphate *N*-oxide; **MeCN** – acetonitrile; **TFA** – trifluoroacetic acid; **THF** – tetrahydrofuran; **TIPS** – triisopropylsilane; **TSTU** – O-(N-Succinimidyl)-*N*,*N*,*N′*,*N′*-tetramethyluronium tetrafluoroborate; **SPhos** – 2-dicyclohexylphosphino-2′,6′-dimethoxybiphenyl, **Xantphos** – 4,5-Bis(diphenylphosphino)-9,9-dimethylxanthene; **NBS** – *N*-bromosuccinimide.

Synthesis of Clozapine tracers

Synthesis of Clozapine-a fluorescent tracer

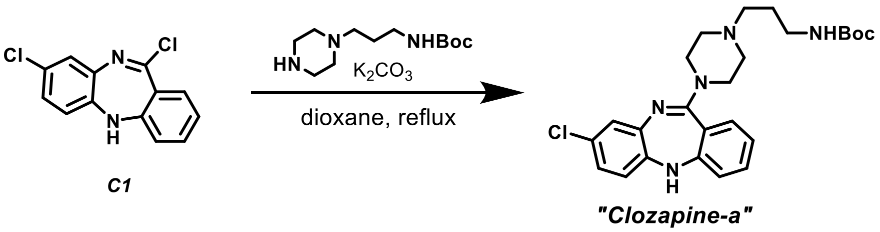

A 10 mL microwave vial, equipped with stir bar, was charged with **C1[5]** (56 mg, 0.21 mmol),tert-butyl (3-(piperazin-1-yl)propyl)carbamate (104 mg, 126 μmol), K_2CO_3 (74 mg, 0.53 mmol), and dioxane (4 mL). The vial was placed into a microwave reactor and heated to 120°C for 1 hour. HPLC analysis confirmed consumption of the starting material, and then the solution was filtered and concentrated under reduced pressure. The crude residue was purified by flash chromatography (gradient elution, 0→20% MeOH/DCM) yielding 72 mg (73% yield) of **Clozapine-a** as a yellow solid. ^{1}H NMR (400 MHz, DMSO-*d*$_6$) δ 7.33 (td, J = 7.8, 1.5 Hz, 1H), 7.26 – 7.10 (m, 2H), 7.09 – 6.92 (m, 2H), 6.87 – 6.71 (m, 3H), 2.94 (app. q, J = 6.6 Hz, 2H), 2.42 (s, 3H partial overlap with DMSO-*d*$_5$), 2.30 (t, J = 7.3 Hz, 2H partial overlap with DMSO-*d*$_5$-^{12}C), 1.66 – 1.46 (m, 2H), 1.37 (s, 9H); HRMS (ESI) calc'd for $C_{25}H_{33}ClN_5O_2$ $[M+H]^+$ 470.2323 found 470.2300.

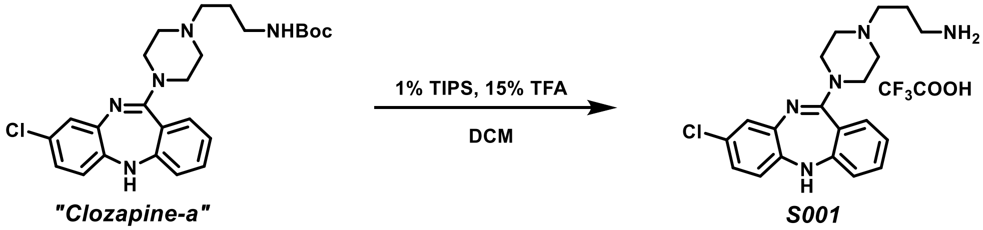

A 50 mL flask, equipped with stir bar, was charged with **Clozapine-a** (72 mg, 0.15 mmol) and a cleavage cocktail (10 mL, 85:15:1 DCM/TFA/TIPS). The resulting light-yellow solution was stirred at 22°C for 2 hours, at which point, HPLC indicated complete consumption of the staring material, and the solvent removed under reduced pressure. The residue was dissolved in 10 mL MeOH, and solvent removed under reduced pressure to provide 72 mg (97% yield) of **S001** as yellow oil. ^{1}H NMR (400 MHz, DMSO-*d*$_6$) δ 7.81 (s, 2H), 7.50 – 7.36 (m, 2H), 7.32 (dd, J = 7.8, 1.6 Hz, 1H), 7.14 – 6.99 (m, 2H), 6.99 – 6.76 (m, 3H), 3.96 (br. s, 1H), 3.54 (br. s. 1H), 3.31 (br. s, 5H), 2.53-2.51 (m, 2H, overlap with DMSO-*d*$_5$) 2.88 (m, 2H), 2.00 – 1.87 (m, 2H); HRMS (ESI) calc'd for $C_{20}H_{25}ClN_5$ $[M+H]^+$ 370.1798 found 370.1790.

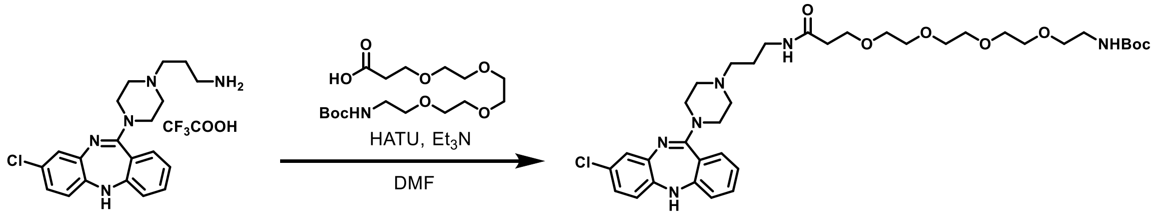

A 25 mL flask, equipped with stir bar, was charged with **S001** (12 mg, 25 µmol), 2,2-dimethyl-4 oxo-3,8,11,14,17-pentaoxa-5-azaicosan-20-oic acid (14 mg, 37 µmol), HATU (12 mg, 31 µmol), NEt_3 (24 µL, 0.17 mmol), and DMF (6 mL). The resulting light-yellow solution was stirred at 22°C for 1 hour, at which point, HPLC indicated complete consumption of the staring material, and the solvent removed under reduced pressure. The crude residue was purified by preparative HPLC (C18, 5 → 95% $MeCN/H_2O$, 0.05% TFA) yielding 18 mg (quantitative yield) of amide **S002** as a yellow oil. ^1H NMR (400 MHz, MeOD) δ 7.58 – 7.51 (m, 1H), 7.47 (dd, J = 7.8, 1.6 Hz, 1H), 7-227.20 (m 1H), 7.20 – 7.08 (m, 3H), 6.97 (d, J = 8.5 Hz, 1H), 3.94 (br. s, 4H), 3.76 (t, J = 5.8 Hz, 2H), 3.61 (m, 12H), 3.49 (m, 6H), 3.38 (t, J = 6.3 Hz, 2H), 3.28 – 3.03 (m, 4H), 2.50 (t, J = 5.8 Hz, 2H), 2.11 – 1.86 (m, 2H), 1.43 (s, 9H). MS (ESI) calc'd for $C_{36}H_{54}ClN_6O_7$ $[M+H]^+$ 717.37 found 717.58.

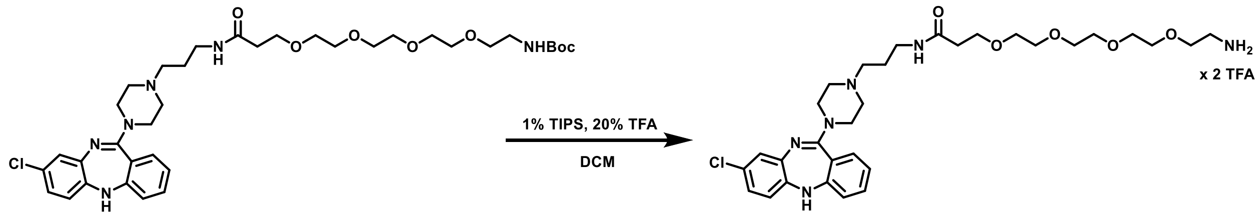

A 25 mL flask, equipped with stir bar, was charged with **S002** (18 mg, 25 µmol) and a cleavage cocktail (10 mL, 80:20:1 DCM/TFA/TIPS). The resulting light-yellow solution was stirred at 22°C for 1 hour, at which point, HPLC indicated complete consumption of the staring material, and the solvent removed under reduced pressure. The residue was dissolved in 10 mL MeOH, and solvent removed under reduced pressure to provide 18 mg (quantitative yield) of primary amine **S003** as a yellow oil. This material was further used without additional purification. HRMS (ESI) calc'd for $C_{31}H_{46}ClN_5O_5$ $[M+H]^+$ 617.32 found 617.38.

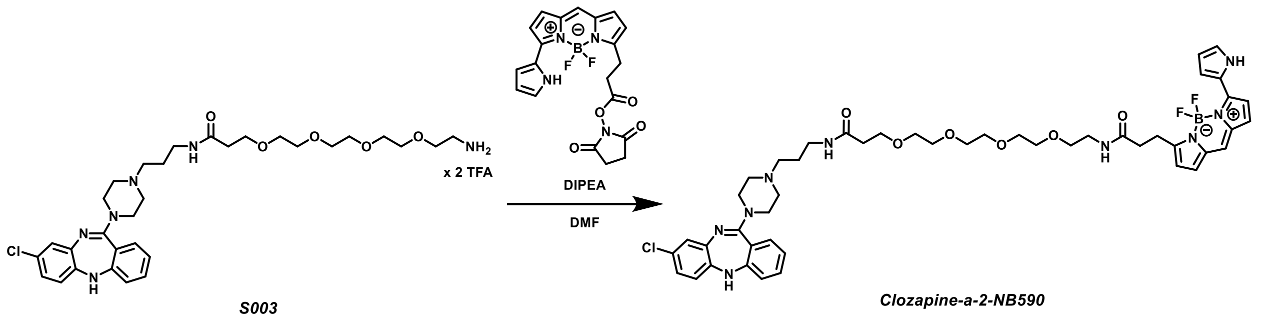

A 25 mL flask, equipped with stir bar, was charged with **S003** (7.0 mg, 11 μmol), **BODIPY590 SE** (4.9 mg, 11 μmol), DIPEA (14 μL, 79 μmol), and DMF (6 mL). The resulting deep purple solution was stirred at 22°C for 1 hour, at which point, HPLC indicated complete consumption of the staring material, and the reaction mixture purified by preparative HPLC (C18, 5 → 95% MeCN/H_2O, 0.05% TFA) yielding 1.8 mg (17% yield) of **Clozapine-a-2-NB590** as a deep purple film. ^{1}H NMR (400 MHz, MeOD) δ 7.43 (ddd, J = 8.0, 7.4, 1.6 Hz, 1H), 7.33 (dd, J = 7.8, 1.5 Hz, 1H), 7.24 (s, 1H), 7.20 (m, 3H), 7.09 (dd, J = 7.5, 1.2 Hz, 1H), 7.07 – 7.03 (m, 2H), 7.02 (d, J = 4.6 Hz, 1H), 6.99 (dd, J = 8.5, 2.4 Hz, 1H), 6.93 (d, J = 4.0 Hz, 1H), 6.87 (d, J = 8.4 Hz, 1H), 6.35 (m, 2H), 4.20 – 3.72 (br.s, 2H), 3.71 (t, J = 5.8 Hz, 2H), 3.64 – 3.55 (m, 14H), 3.53 (m, 3H), 3.37 (t, J = 5.5 Hz, 4H), 3.28 (m, 4H), 3.14 (d, J = 14.3 Hz, 3H), 2.65 (dd, J = 8.3, 7.1 Hz, 2H), 2.45 (t, J = 5.8 Hz, 2H), 1.92 (p, J = 6.8 Hz, 2H). HRMS (ESI) calc'd for $C_{47}H_{57}BClF_2N_9O_6Na$ $[M+Na]^+$ 950.4079 found 9050.4050.

Synthesis of Clozapine-b fluorescent tracer

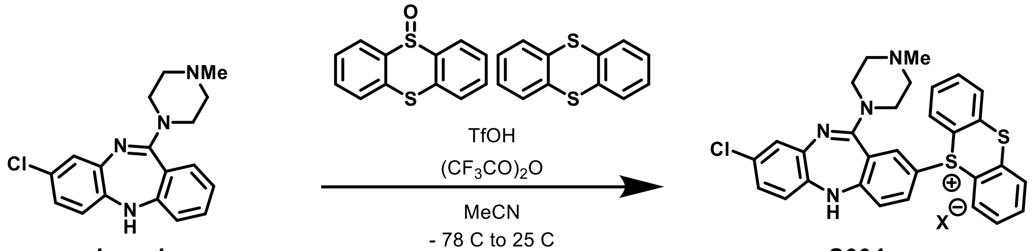

A 20 ml glass-vial was charged with clozapine (0.20 mg, 0.61 mmol), and MeCN (5 ml). After cooling to –78 °C, trifluoroacetic anhydride (0.19 ml, 1.4 mmol) was added to the frozen the reaction mixture. Thianthrene (4 mg, 19 μmol), thianthrene-S-oxide (138 mg, 0.59 mmol) were added, followed by the addition of triflic acid (230 µl, 2.6 mmol, 4.2 equiv.) in one portion at –78 °C. The vial was sealed with a screw-cap, and the mixture was allowed to stand at –78 °C for 1.5 hours, followed by warming the reaction mixture to 25 °C over a period of 1.5 hours. The reaction mixture was concentrated under reduced pressure and dissolved in DCM (10 ml) followed by addition of saturated aqueous $NaHCO_3$ solution (10 ml). The organic layers were separated, and the aqueous layer was extracted into DCM (2 x 10 mL). The DCM fractions were combined,

dried over $MgSO_4$, filtered, and the solvent was removed under reduced pressure. The crude residue was purified by silica gel chromatography (0→100% MeOH/DCM) to provide 143 mg (43% yield) of **S004** as a white solid. ^{1}H NMR (400 MHz, MeOD) δ 8.43 (dd, J = 7.9, 1.3 Hz, 2H), 8.05 (d, J = 7.8 Hz, 2H), 7.95 (td, J = 7.7, 1.3 Hz, 2H), 7.86 (td, J = 7.7, 1.3 Hz, 2H), 7.15 (dd, J = 8.8, 2.6 Hz, 1H), 7.04 (d, J = 8.8 Hz, 1H), 6.94 (d, J = 2.6 Hz, 1H), 6.92 (d, J = 2.5 Hz, 1H), 6.87 (dd, J = 8.4, 2.4 Hz, 1H), 6.71 (d, J = 8.4 Hz, 1H), 3.13 (s, 4H), 2.32 (m, J = 14.3 Hz, 7H). MS (ESI) calc'd for $C_{30}H_{26}ClN_4S_2$ $[M+H]^+$ 541.1 found 541.4. Modification assignment was confirmed by COSY and NOESY experiments.

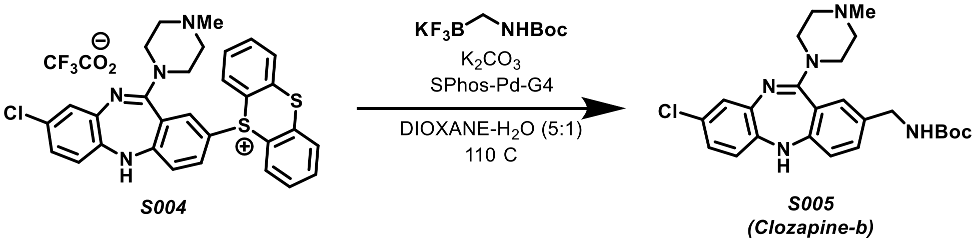

A 40 mL vial, equipped with stir bar, was charged under Ar with **S004** (157 mg, 240 μmol), potassium (Boc-amino-methyl)trifluoroborate (57 mg, 240 μmol) , S-Phos-Pd-G4 (9.5 mg, 12 μmol), K_2CO_3, degassed dioxane (10 mL), and degassed water (2 mL). The vial was heated at 110°C for 18 hours. The cooled solution was filtered, and solvents removed under reduced pressure. The reaction mixture was purified by preparative RP HPLC (5→95% MeCN/H_2O buffered with 0.5% TFA) to provide 10 mg (9 % yield) of **S005** as yellow oil. ^{1}H NMR (400 MHz, MeOD) δ 7.24 (dd, J = 8.2, 2.1 Hz, 1H), 7.19 (d, J = 2.1 Hz, 1H), 6.98 – 6.88 (m, 2H), 6.84 (dd, J = 8.4, 2.4 Hz, 1H), 6.78 (d, J = 8.4 Hz, 1H), 4.13 (s, 2H), 3.43 (s, 4H), 2.59 (s, 4H), 2.37 (s, 3H), 1.45 (s, 9H); HRMS (ESI) calc'd for $C_{24}H_{31}ClN_5O_2$ [M+H]+ 456.2166 found 456.2156.

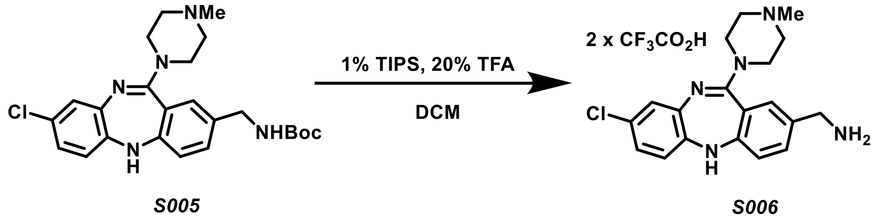

A 25 mL flask, equipped with stir bar, was charged with carbamate **S005** (10 mg, 22 μmol) and a cleavage cocktail (5 mL, 80:20:1 DCM/TFA/TIPS). The resulting light-yellow solution was stirred at 22°C for 2 hours, at which point, HPLC indicated complete consumption of the staring material, and the solvent removed under reduced pressure. The residue was dissolved in 10 mL MeOH, and the solvent removed under reduced pressure to provide primary amine **S006** as yellow oil, which was further used without additional purification. MS (ESI) calc'd for $C_{19}H_{23}ClN_5$ $[M+H]^+$ 356.2 found 356.1.

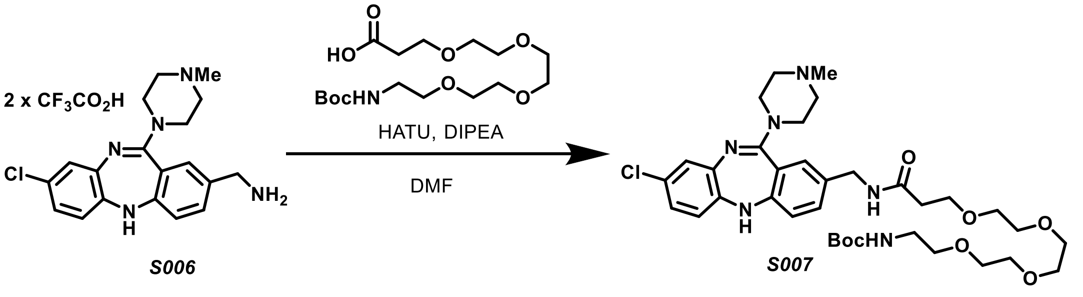

A 25 mL flask, equipped with stir bar, was charged with **S006** (7.0 mg, 15 mmol), 2,2-dimethyl-4-oxo-3,8,11,14,17-pentaoxa-5-azaicosan-20-oic acid (8 mg, 20 µmol), HATU (7 mg, 0.02 mmol), DIPEA (15 µL, 0.10 mmol), and DMF (6 mL). The resulting light-yellow solution was stirred at 22°C for 1.5 hours, at which point, HPLC indicated complete consumption of the staring material, and the solvent removed under reduced pressure. The crude residue was purified by preparative HPLC (C18, 5 → 95% MeCN/H_2O, 0.05% TFA) yielding 11 mg (quantitative) of carbamate **S007** as a yellow oil. ^1H NMR (400 MHz, MeOD) δ 7.45 (dd, J = 8.3, 2.0 Hz, 1H), 7.36 (d, J = 2.0 Hz, 1H), 7.18 (d, J = 2.4 Hz, 1H), 7.14 – 7.03 (m, 2H), 6.94 (d, J = 8.6 Hz, 1H), 4.32 (s, 2H), 3.85 (s, 3H), 3.75 (dt, J = 15.5, 6.0 Hz, 4H), 3.68 – 3.43 (m, 26H), 3.21 (dt, J = 11.2, 5.6 Hz, 3H), 3.01 (s, 3H), 2.55 (t, J = 6.3 Hz, 1H), 2.49 (t, J = 5.9 Hz, 2H), 1.43 (d, J = 6.8 Hz, 14H); MS (ESI) calc'd for $C_{35}H_{52}ClN_6O_7$ [M+H]+ 703.4 found 703.6.

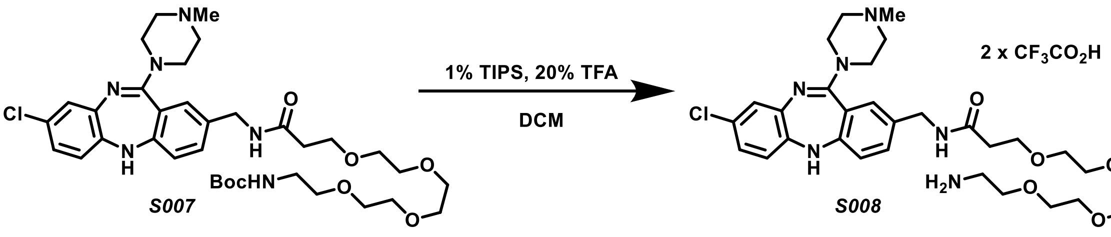

A 25 mL flask, equipped with stir bar, was charged with **S007** (16 mg, 23 µmol) and a cleavage cocktail (10 mL, 80:20:1 DCM/TFA/TIPS). The resulting light-yellow solution was stirred at 22°C for 1 hour, at which point, HPLC indicated complete consumption of the staring material, and the solvent removed under reduced pressure. The residue was dissolved in 10 mL MeOH, and the solvent removed under reduced pressure to provide 16 mg of primary amine **S008** as a yellow oil. This material was further used without additional purification.

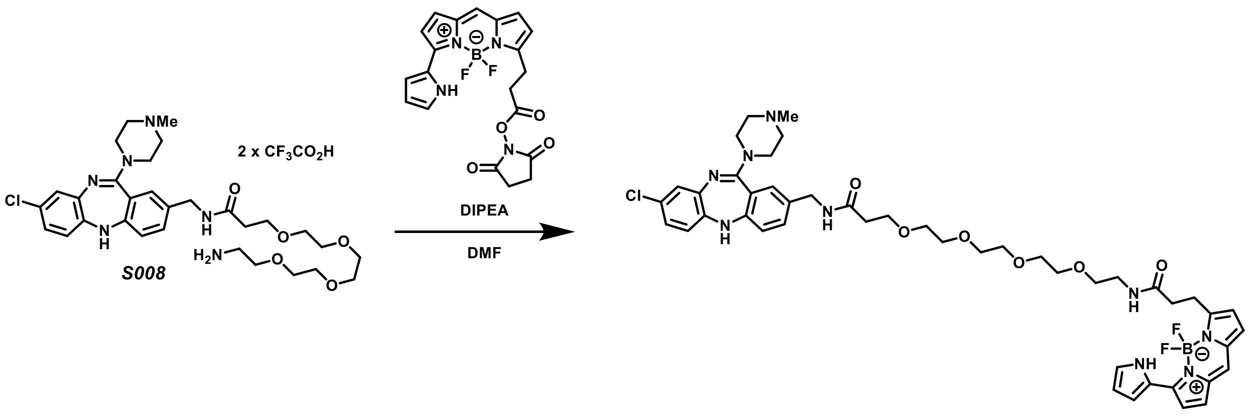

Clozapine-b-2-NB590

A 25 mL flask, equipped with stir bar, was charged with **S008** (7 mg, 12 μmol), **BODIPY590 SE** (5.0 mg, 12 μmol), DIPEA (15 μL, 82 μmol), and DMF (6 mL). The resulting deep purple solution was stirred at 22°C for 18 hours, at which point, HPLC indicated complete consumption of the staring material, and the reaction mixture purified by preparative HPLC (C18, 5 → 95% MeCN/H_2O, 0.05% TFA) yielding 2.3 mg (23% yield) of **Clozapine-b-2-NB590** as a deep purple film. ^{1}H NMR (400 MHz, MeOD) δ 7.38 (dd, J = 8.2, 2.1 Hz, 1H), 7.29 (d, J = 2.0 Hz, 1H), 7.24 (s, 1H), 7.23 – 7.13 (m, 3H), 7.10 (d, J = 2.4 Hz, 1H), 7.06 – 6.97 (m, 3H), 6.92 (d, J = 4.0 Hz, 1H), 6.89 (d, J = 8.4 Hz, 1H), 6.54 – 6.21 (m, 2H), 4.29 (s, 2H), 3.96 – 3.58 (m, 6H), 3.58 – 3.45 (m, 15H), 3.43 (s, 3H), 3.36 (t, J = 5.4 Hz, 3H), 3.27 (d, J = 7.7 Hz, 2H), 2.95 (s, 3H), 2.64 (t, J = 7.7 Hz, 2H), 2.45 (t, J = 5.8 Hz, 2H); HRMS (ESI) calc'd for $C_{46}H_{56}BClF_2N_9O_6$ $[M+H]^+$ 914.4103 found 914.4089.

Synthesis of Clozapine-c fluorescent tracer

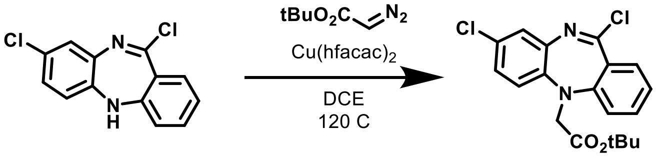

C1 *S009*

A 10 mL microwave vial, equipped with stir bar, was charged with **C1** (18 mg, 68 μmol), $Cu(hfacac)_2$ (3.3 mg, 6.9 μmol), and DCE (2 mL). The vial was sealed, and tert-Butyl diazoacetate (28 μL, 0.21 mmol) slowly added to a stirred solution (gas evolution may occur). The vial was placed into a microwave reactor and heated to 120°C for 1 minute (ramp to 120°C takes 2 minutes). The cooled solution was purified by flash chromatography (gradient elution, 0 → 20% EtOAc/heptane, yielding 10 mg (39% yield) of ester **S009** as a yellow solid. ^{1}H NMR (400 MHz, $CDCl_3$) δ 7.65 (dd, J = 7.8, 1.6 Hz, 1H), 7.42 (ddd, J = 8.2, 7.4, 1.6 Hz, 1H), 7.22 (d, J = 2.5 Hz, 1H), 7.19 – 7.08 (m, 2H), 6.91 (dd, J = 8.2, 1.0 Hz, 1H), 6.82 (d, J = 8.6 Hz, 1H), 4.39 (d, J = 16.4

Hz, 1H), 4.26 (d, J = 16.3 Hz, 1H), 1.30 (s, 9H); HRMS (ESI) calc'd for $C_{19}H_{19}Cl_2N_2O_2$ $[M+H]^+$ 377.0818 found 377.0809.

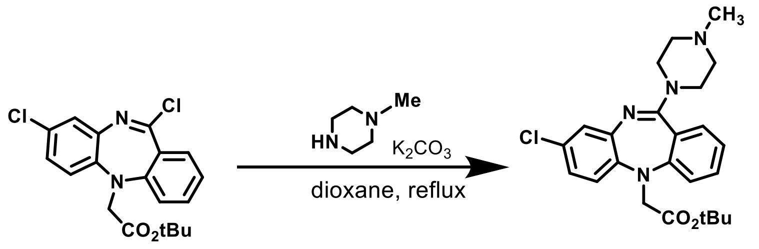

S009

S010
(Colzapine-c)

A 20 mL microwave vial, equipped with stir bar, was charged with **S009** (54 mg, 0.14 mmol), 1-methylpiperazine (80 µL, 0.72 mmol), K_2CO_3 (50 mg, 0.36 mmol), and dioxane (10 mL). The vial was placed into a microwave reactor and heated to 120°C for 2 hours. The cooled solution was filtered, and the solvent removed under reduced pressure. The crude residue was purified by flash chromatography (gradient elution, 0→30% MeOH/DCM, yielding 50 mg (79% yield) of amidine **Clozapine-c** as a grey solid. ^1H NMR (400 MHz, MeOD) δ 7.42 (ddd, J = 8.2, 7.3, 1.6 Hz, 1H), 7.36 – 7.23 (m, 1H), 7.23 – 7.04 (m, 2H), 6.98 (dd, J = 1.9, 1.0 Hz, 1H), 6.94 – 6.80 (m, 2H), 4.53 (d, J = 16.8 Hz, 1H), 4.25 (d, J = 16.7 Hz, 1H), 3.61 – 3.40 (m, 4H), 2.69 – 2.44 (m, 4H), 2.34 (s, 3H), 1.38 (s, 9H); HRMS (ESI) calc'd for $C_{24}H_{30}ClN_4O_2$ $[M+H]^+$ 441.2057 found 441.2042.

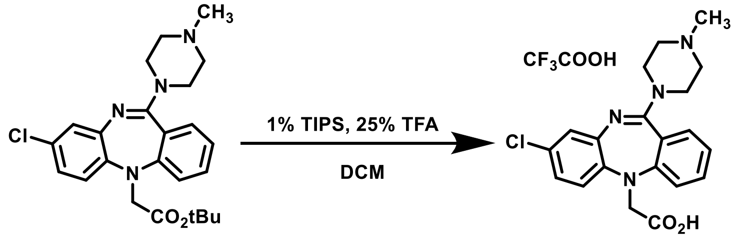

S010
(Colzapine-c)

S011

A 25mL flask, equipped with stir bar, was charged with amidine **Clozapine-c** (5.4 mg, 12 µmol) and a cleavage cocktail (10 mL, 75:25:1 DCM/TFA/TIPS). The resulting light-yellow solution was stirred at 22°C for 3 hours, at which point, HPLC indicated complete consumption of the staring material, and the solvent removed under reduced pressure. The residue was dissolved in 10 mL MeOH, and the solvent removed under reduced pressure to provide carboxylic acid **S011** as yellow oil, which was used in the following steps without further purification. ^1H NMR (400 MHz, DMSO-d_6) 7.62 (ddd, J = 8.7, 7.3, 1.6 Hz, 1H), 7.50 (dd, J = 7.8, 1.6 Hz, 1H), 7.38 (dd, J = 8.3, 1.0 Hz, 1H), 7.29 (td, J = 7.6, 1.1 Hz, 1H), 7.26 – 7.19 (m, 2H), 7.16 (d, J = 8.7 Hz, 1H), 4.79 (d, J = 17.5 Hz, 1H), 4.48 (d, J = 17.6 Hz, 1H), 4.29 – 3.71 (m, 4H), 3.68 – 3.38 (m, 4H), 2.98 (s, 3H).

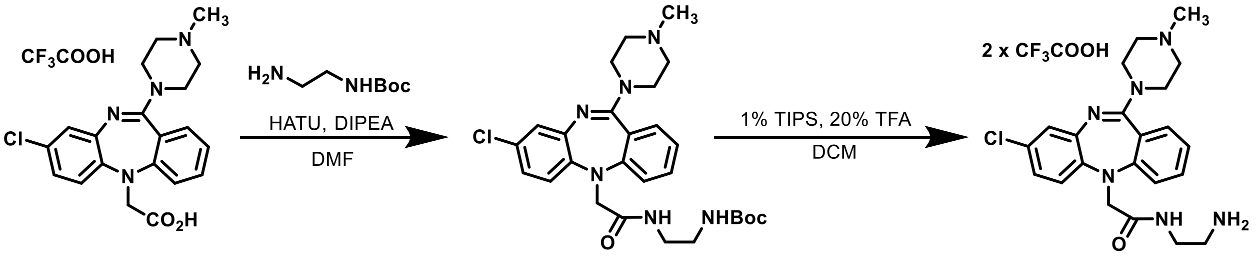

S011 *S012* *S013*

A 25 mL flask, equipped with stir bar, was charged with **S011** (20 mg, 52 μmol), tert-butyl (2-aminoethyl)carbamate (10 mg, 65 μmol), HATU (25 mg, 65 μmol), DIPEA (65 μL, 0.36 mmol), and DMF (6 mL). The resulting light-yellow solution was stirred at 22°C for 3 hours, at which point, HPLC indicated complete consumption of the staring material, and the solvent removed under reduced pressure. The crude residue was purified by flash chromatography (gradient elution, 0→ 20% MeOH/DCM, yielding 3 mg (11% yield) of amide **S012** as yellow oil. ^{1}H NMR (400 MHz, MeOD) δ 7.47 (ddd, J = 8.6, 7.3, 1.6 Hz, 1H), 7.35 (dd, J = 7.7, 1.6 Hz, 1H), 7.28 – 7.12 (m, 2H), 7.03 (d, J = 2.2 Hz, 1H), 7.01 – 6.88 (m, 2H), 4.40 (d, J = 15.8 Hz, 1H), 4.31 (d, J = 15.7 Hz, 1H), 3.82 – 3.38 (m, 4H), 3.19 (m, 2H), 3.08 – 2.95 (m, 2H), 2.68 (s, 2H), 2.59 (m, 2H), 2.40 (s, 3H), 1.39 (s, 9H); HRMS (ESI) calc'd for $C_{27}H_{36}ClN_6O_3$ $[M+H]^+$ 527.2537 found 527.2528.

A 25 mL flask, equipped with stir bar, was charged with amide **S012** (6 mg, 11 μmol) and a cleavage cocktail (10 mL, 80:20:1 DCM/TFA/TIPS). The resulting light-yellow solution was stirred at 22°C for 1 hour, at which point, HPLC indicated complete consumption of the staring material, and the solvent removed under reduced pressure. The residue was dissolved in 10 mL MeOH, and the solvent removed under reduced pressure to provide primary amine **S013** as yellow oil, which was used in the following steps without further purification. ^{1}H NMR (400 MHz, MeOD) δ 7.98 (s, 3H), 7.63 (ddd, J = 8.6, 7.4, 1.6 Hz, 1H), 7.52 (dd, J = 7.8, 1.6 Hz, 1H), 7.39 – 7.29 (m, 2H), 7.27 (d, J = 2.4 Hz, 1H), 7.22 (dd, J = 8.7, 2.4 Hz, 1H), 7.14 (d, J = 8.7 Hz, 1H), 4.60 (d, J = 15.7 Hz, 1H), 4.45 (d, J = 15.8 Hz, 1H), 3.97 (br. s, 4H), 3.70 – 3.47 (m, 4H), 3.44 (td, J = 6.2, 4.6 Hz, 2H), 3.05-3.01 (m, 2H), 3.00 (s, 4H); MS (ESI) calc'd for $C_{22}H_{28}ClN_6O$ [M+H]+ 427.20 found 427.09.

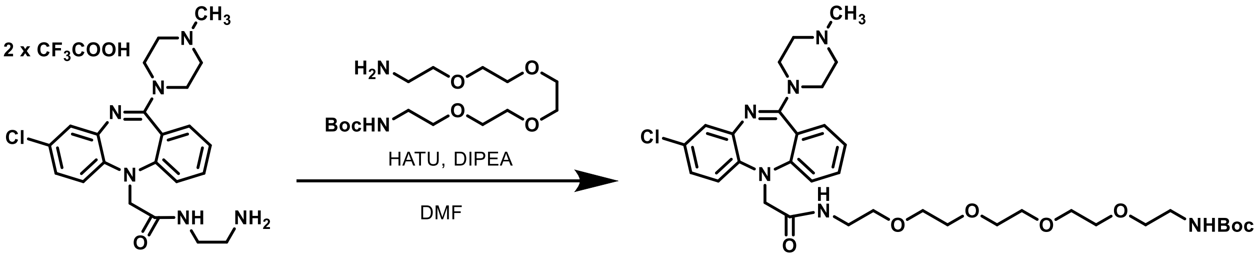

S013 *S014*

A 25 mL flask, equipped with stir bar, was charged with **S013** (43 mg, 0.11 mmol), tert-butyl (14-amino-3,6,9,12-tetraoxatetradecyl)carbamate (56 mg, 0.17 mmol), HATU (53 mg, 0.14

mmol), DIPEA (140 µL, 0.78 mmol), and DMF (8 mL). The resulting light-yellow solution was stirred at 22°C for 21 hours, at which point, HPLC indicated complete consumption of the staring material, and the solvent removed under reduced pressure. The crude residue was purified by preparative HPLC (C18, 5 → 95% MeCN/H_2O, 0.05% TFA), yielding 47 mg (60% yield) of carbamate **S014** as a clear oil. ^{1}H NMR (400 MHz, MeOD) δ 7.54 (ddd, J = 8.2, 7.3, 1.6 Hz, 1H), 7.42 (dd, J = 7.7, 1.6 Hz, 1H), 7.30 – 7.21 (m, 2H), 7.15 – 7.06 (m, 2H), 7.04 (d, J = 8.6 Hz, 1H), 4.54 (d, J = 15.5 Hz, 1H), 4.31 (d, J = 15.6 Hz, 1H), 3.73 – 3.56 (m, 11H), 3.56 – 3.39 (m, 10H), 3.21 (t, J = 5.7 Hz, 2H), 2.99 (s, 3H), 1.43 (s, 9H); MS (ESI) calc'd for $C_{35}H_{52}ClN_6O_7$ $[M+H]^+$ 703.36 found 703.44.

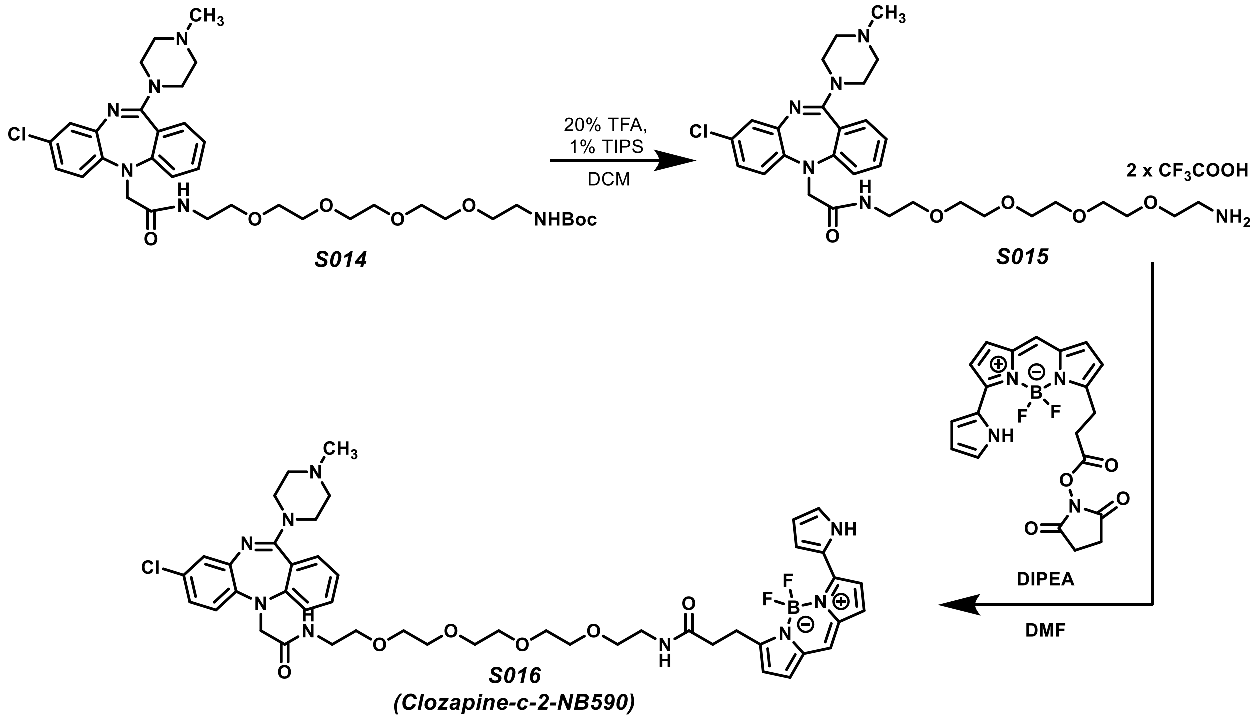

A 25 mL flask, equipped with stir bar, was charged with **S014** (47 mg, 67 µmol) and a cleavage cocktail (10 mL, 80:20:1 DCM/TFA/TIPS). The resulting light-yellow solution was stirred at 22°C for 1 hour, at which point, HPLC indicated complete consumption of the staring material, and the solvent removed under reduced pressure. The residue was dissolved in 10 mL MeOH, and the solvent removed under reduced pressure to provide 56 mg (quantitative yield) of primary amine **S015** as a yellow oil. This material was further used without additional purification. HRMS (ESI) calc'd for $C_{30}H_{44}ClN_{6NH2}O_5$ $[M+H]^+$ 603.31 found 603.24.

A 25 mL flask, equipped with stir bar, was charged with **S015** (10 mg, 17 µmol), **NanoBRET590 SE** (7.1 mg, 17 µmol), DIPEA (20 µL, 0.12 mmol), and DMF (6 mL). The resulting deep purple solution was stirred at 22°C for 18 hours, at which point, HPLC indicated complete consumption of the staring material, and the reaction mixture purified by preparative HPLC (C18, 5 → 95% MeCN/H_2O, 0.05% TFA), yielding 4.8 mg (32% yield) of amide **Clozapine-c-2-NB590** as a deep purple film. ^{1}H NMR (400 MHz, MeOD) δ 7.53 (ddd, J = 8.2,

7.3, 1.6 Hz, 1H), 7.40 (dd, J = 7.7, 1.6 Hz, 1H), 7.29 – 7.17 (m, 6H), 7.15 – 7.06 (m, 2H), 7.06 – 6.97 (m, 2H), 6.93 (d, J = 4.0 Hz, 1H), 6.34 (td, J = 4.3, 3.8, 1.8 Hz, 2H), 4.48 (d, J = 15.6 Hz, 1H), 4.28 (d, J = 15.6 Hz, 1H), 4.14 – 3.65 (m, 4H), 3.64 – 3.56 (m, 8H), 3.56 – 3.33 (m, 14H), 2.95 (s, 3H), 2.72 – 2.58 (m, 2H).; HRMS (ESI) calc'd for $C_{46}H_{56}BClF_2N_9O_6$ $[M+H]^+$ 914.4103 found 914.4111.

Synthesis of Amitriptyline fluorescent tracers

Synthesis of Amitriptyline-a and Amitriptyline-b fluorescent tracers

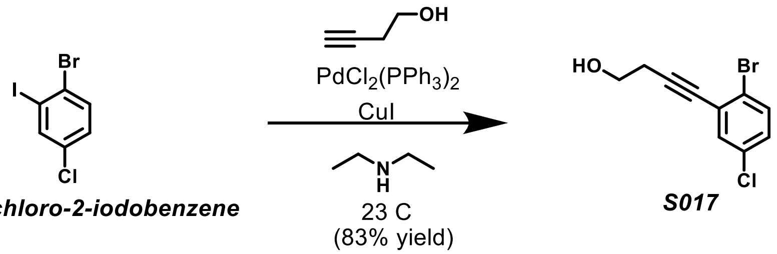

1-bromo-4-chloro-2-iodobenzene ***S017***

A 50 mL round bottom flask, equipped with stir bar and rubber septum under argon atmosphere, was charged with 1-bromo-4-chloro-2-iodobenzene (3.17 g, 10 mmol), CuI (38 mg, 0.2 mmol), and $PdCl_2(PPh_3)_2$ (140 mg, 0.2 mmol). Degassed diethylamine (18 mL) was added via syringe followed by 3-butyn-1-ol. The reaction mixture was stirred at 23ºC for 72 hours at which point HPLC indicated complete consumption of the staring material, and the solvent removed under reduced pressure. The crude residue was purified by flash chromatography (gradient elution, 0 → 50% EtOAc/heptane, yielding 2.15 g (83% yield) of alkyne **S017** as a yellow solid. ^{1}H NMR (400 MHz, CDCl3) δ 7.49 (d, J = 8.6 Hz, 1H), 7.43 (d, J = 2.6 Hz, 1H), 7.13 (dd, J = 8.6, 2.5 Hz, 1H), 3.85 (t, J = 6.1 Hz, 2H), 2.74 (t, J = 6.1 Hz, 2H); ^{13}C NMR (100 MHz, CDCl3) δ 133.3, 133.0, 132.9, 129.3, 126.8, 123.6, 93.1, 80.4, 77.3, 77.0, 76.7, 60.9, 24.0; HRMS (ESI) calc'd for $C_{10}H_9BrClO$ $[M+H]^+$ 258.9525 found 258.9520.

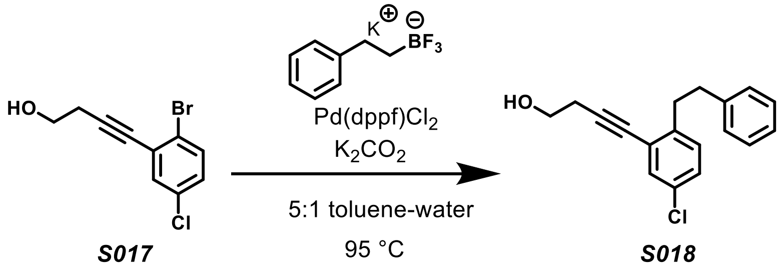

S017 ***S018***

A 50 mL pressure vessel, equipped with stir bar, was charged with **S017** (146 mg, 560 μmol), potassium trifluoro(phenethyl)borate (125 mg, 590 μmol), K_2CO_3 (206 mg,1.69 mmol),

Pd(dppf)Cl_2 (8 mg, 11 μmol). Headspace was flushed with argon and degassed toluene (5 mL) and degassed water (1 mL) were added. The reaction mixture was stirred at 95ºC for 21 hours. The reaction mixture was cooled to ambient temperature, diluted with EtOAc (40 mL), and dried with $MgSO_4$, filtered, and the solvent removed under reduced pressure. The crude residue was purified by preparative HPLC (C18, 5 → 95% MeCN/H_2O, 0.05% TFA) yielding 60 mg (38% yield) of alkyne **S018** as a clear oil. ^{1}H NMR (400 MHz, CDCl3) δ 7.39 (d, J = 2.3 Hz, 1H), 7.33 – 7.26 (m, 2H), 7.24 – 7.19 (m, 1H), 7.16 (dd, J = 8.4, 2.0 Hz, 3H), 7.04 (d, J = 8.2 Hz, 1H), 3.83 (t, J = 6.3 Hz, 2H), 3.13 – 2.95 (m, 2H), 2.90 (dd, J = 9.7, 6.2 Hz, 2H), 2.74 (t, J = 6.3 Hz, 2H); ^{13}C NMR (100 MHz, CDCl3) δ 13C NMR (101 MHz, CDCl3) δ 142.0, 141.4, 132.0, 131.4, 130.1, 128.4, 128.4, 128.1, 126.0, 124.4, 91.2, 79.8, 61.2, 36.8, 36.2, 23.9; HRMS (ESI) calc'd for $C_{18}H_{18}ClO$ $[M+H]^+$ 285.1046 found 285.1044.

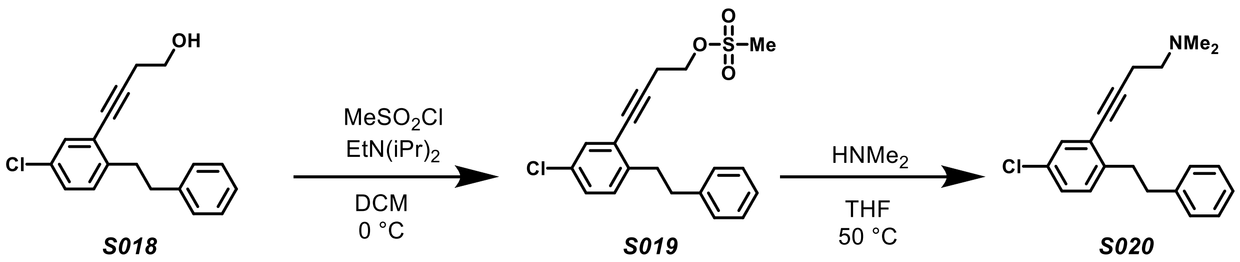

A 25 mL round bottom flask, equipped with stir bar, was charged with **S018** (72 mg, 0.25 mmol), $EtN(iPr)_2$ (90 μL, 0.51 mmol), and DCM (7 mL). The resulting solution was cooled to 0ºC under argon followed by addition of mesyl chloride (29 μL, 0.38 mmol). The reaction mixture was stirred at 0 °C for 1 hour at which point HPLC indicated complete consumption of the staring material, and the solvent removed under reduced pressure. The crude mesylate **S019** was used without additional purification in the next step.

A 25 mL round bottom flask, equipped with stir bar, was charged with **S019** (90 mg, 0.25 mmol) and 2M dietylamine solution in THF (12 mL, 25 mmol). The reaction mixture was stirred at 50 °C for 20 hours at which point HPLC indicated complete consumption of the staring material, and the solvent removed under reduced pressure. The crude residue was purified by flash chromatography (gradient elution, 0 →100% EtOAc/heptane, yielding 26 mg (35% yield) of amine **S020** as a clear oil. ^{1}H NMR (400 MHz, CDCl3) δ 7.37 (d, J = 2.3 Hz, 1H), 7.28 (dd, J = 8.0, 6.6 Hz, 2H), 7.23 – 7.16 (m, 3H), 7.14 (dd, J = 8.2, 2.3 Hz, 1H), 7.02 (d, J = 8.2 Hz, 1H), 3.07 – 2.95 (m, 2H), 2.95 – 2.81 (m, 2H), 2.64 (s, 4H), 2.31 (s, 6H); ^{13}C NMR (100 MHz, CDCl3) δ 13C NMR (101 MHz, CDCl3) δ 142.0, 141.6, 131.9, 131.3, 130.0, 128.4, 128.3, 127.9, 126.0, 124.8, 78.8, 77.3, 77.0, 76.7, 58.3, 45.1, 36.7, 36.2, 18.5; HRMS (ESI) calc'd for $C_{20}H_{23}ClN$ $[M+H]^+$ 312.1519 found 312.1516.

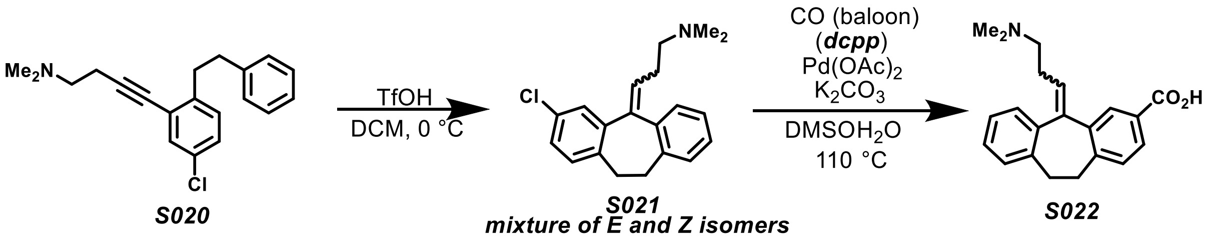

A 10 mL round bottom flask, equipped with stir bar, was charged with a solution of **S020** (25 mg, 80 µmol) in DCM (3 mL). The solution was cooled to 0ºC, and triflic acid (39 µL, 0.44 mmol) was added in one portion. The resulting brown solution is stirred at 0 °C for 10 minutes at which point the reaction was quenched by addition of saturated aqueous solution of K_2CO_3 (3mL). Organic layer was separated, and aqueous solution was extracted (2 X 3 mL DCM). Organics were combined, dried over $MgSO_4$, filtered and concentrated *in vacuo*. The crude residue **S021** was used in the next step without further purification. ^{1}H NMR (400 MHz, $CDCl_3$, reported for mixture of E/Z isomers) δ 7.25 – 6.85 (m, 7H), 5.86 – 5.80 (m, 1H), 3.41 – 3.18 (m, 2H), 3.00 – 2.86 (m, 1H), 2.81 – 2.64 (m, 3H), 2.44 (s, 8H); MS (ESI) calc'd for $C_{10}H_{23}ClN$ $[M+H]^+$ 312.15 found 312.11. Single peak on HPLC at 254 nm.

A 50 mL round bottom flask, equipped with stir bar and septum was charged with **S021** (25 mg, 80 µmol), K_2CO_3 (33 mg, 0.24 mmol), $Pd(OAc)_2$ (1.8 mg, 8.0 µmol) and [dcpp $2BF_4$] (9.8 mg, 16 µmol). Flask was evacuated and backfilled with argon (3x times repeated). Degassed DMSO (2 mL) and H_2O (0.2 mL) were added, and the reaction vessel was evacuated and backfilled with carbon monoxide (3x times repeated). CO was allowed to bubble through the solution for 5 minutes. The resulting yellow suspension was heated to 110°C under CO balloon for 18 hours at which point HPLC analysis indicated complete consumption of the starting material. The reaction mixture was diluted with MeOH (3 mL), passed through syringe filter, and purified by preparative HPLC (C18, 5 → 95% MeCN/H_2O, 0.05% TFA) yielding 25 mg (97% yield) of E/Z mixture of carboxylic acids **S022** as a clear oil. ^{1}H NMR (400 MHz, MeOD, reported for mixture of E/Z isomers) δ 8.06 – 7.67 (m, 2H), 7.49 – 6.92 (m, 5H), 5.89 (m, 1H), 3.49 – 3.34 (m, 2H), 3.25 (m, 2H), 3.09 – 2.90 (m, 1H), 2.81 (d, J = 12.1 Hz, 7H), 2.66 – 2.40 (m, 2H); MS (ESI) calc'd for $C_{21}H_{24}NO_2$ $[M+H]^+$ 322.18 found 322.15.

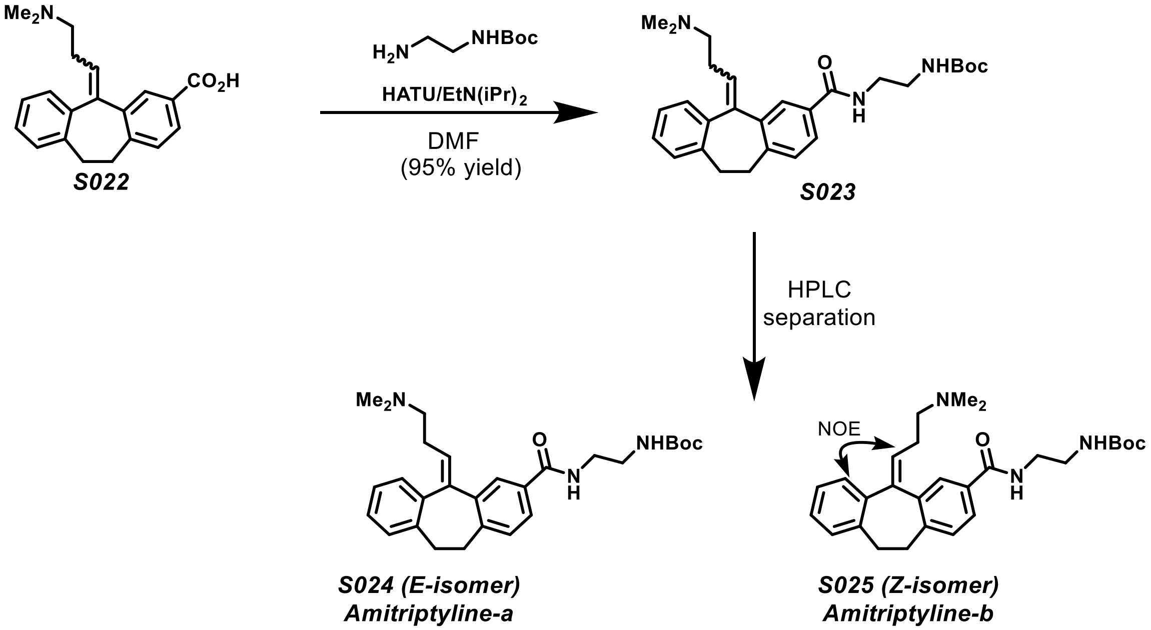

A 25 mL flask, equipped with stir bar, was charged with **S022** (25 mg, 78 μmol), tert-butyl (2-aminoethyl) carbamate (37 mg, 97 μmol), HATU (16 mg, 97 μmol), $EtN(iPr)_2$ (70 μL, 0.39 mmol), and DMF (8 mL). The resulting light-yellow solution was stirred at 22°C for 2.5 hours at which point HPLC indicated complete consumption of the staring material, and the solvent was removed under reduced pressure. The crude residue was purified by preparative HPLC (C18, 5 → 95% MeCN/H2O, 0.05% TFA) yielding 10.6 mg (29% yield) of E-isomer **S024** as a clear oil and 4.7 mg (13% yield) of Z-isomer **S025** al a clear oil (42% combined yield).

S024: ^{1}H NMR (400 MHz, MeOD) δ 7.80 (d, J = 1.9 Hz, 1H), 7.62 (dd, J = 8.0, 2.0 Hz, 1H), 7.34 – 7.20 (m, 3H), 7.20 – 7.12 (m, 2H), 5.92 (t, J = 7.3 Hz, 1H), 3.58 – 3.41 (m, 3H), 3.41 – 3.34 (m, 2H), 3.28 – 3.15 (m, 4H), 3.00 (m, 1H), 2.88 – 2.75 (m, 7H), 2.59 (p, J = 8.3, 7.6 Hz, 2H), 1.41 (s, 9H); MS (ESI) calc'd for $C_{28}H_{38}N_3O_3$ $[M+H]^+$ 464.3 found 464.4.

S025: ^{1}H NMR (400 MHz, MeOD) δ 7.82 – 7.69 (m, 1H), 7.63 (d, J = 1.9 Hz, 1H), 7.40 (d, J = 7.9 Hz, 1H), 7.31 (dd, J = 6.8, 2.4 Hz, 1H), 7.19 (m, 2H), 7.10 (dd, J = 7.2, 1.8 Hz, 1H), 5.89 (t, J = 7.4 Hz, 1H), 3.45 (t, J = 6.0 Hz, 3H), 3.39 (s, 1H), 3.31 – 3.17 (m, 3H), 2.93 (d, J = 13.3 Hz, 2H), 2.88 – 2.71 (m, 6H), 2.71 – 2.31 (m, 2H), 1.41 (s, 9H); MS (ESI) calc'd for $C_{28}H_{38}N_3O_3$ $[M+H]^+$ 464.3 found 464.4.

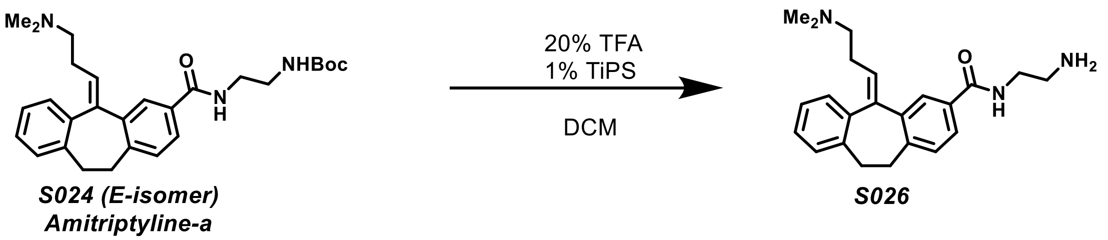

A 25 mL flask, equipped with stir bar, was charged with **S024** (10.6 mg, 22.9 μmol), and the cleavage cocktail (7 mL, 80:20:1 DCM/TFA/TIPS). The resulting light-yellow solution was stirred at 22°C for 35 minutes at which point HPLC indicated complete consumption of the staring material, and the solvent was removed under reduced pressure. The residue was dissolved in 10 mL MeOH, solvent removed under reduced pressure, and the reaction mixture was purified by preparative HPLC (C18, 5 → 95% MeCN/H_2O, 0.05% TFA), yielding 6 mg (72% yield) of amine **S026** as a clear oil. ^{1}H NMR (400 MHz, MeOD) δ 7.85 (d, J = 2.0 Hz, 1H), 7.67 (dd, J = 8.0, 2.0 Hz, 1H), 7.33 – 7.26 (m, 2H), 7.24 (dt, J = 6.6, 3.4 Hz, 1H), 7.22 – 7.14 (m, 2H), 5.92 (t, J = 7.3 Hz, 1H), 3.66 (m, 2H), 3.40 (s, 2H), 3.28 – 3.20 (m, 2H), 3.17 (t, J = 6.0 Hz, 2H), 3.05 – 2.93 (m, 1H), 2.80 (m, 6H), 2.60 (m, 2H); MS (ESI) calc'd for $C_{23}H_{30}N_3O_3^+$ [M+H]$^+$ 364.2 found 364.3.

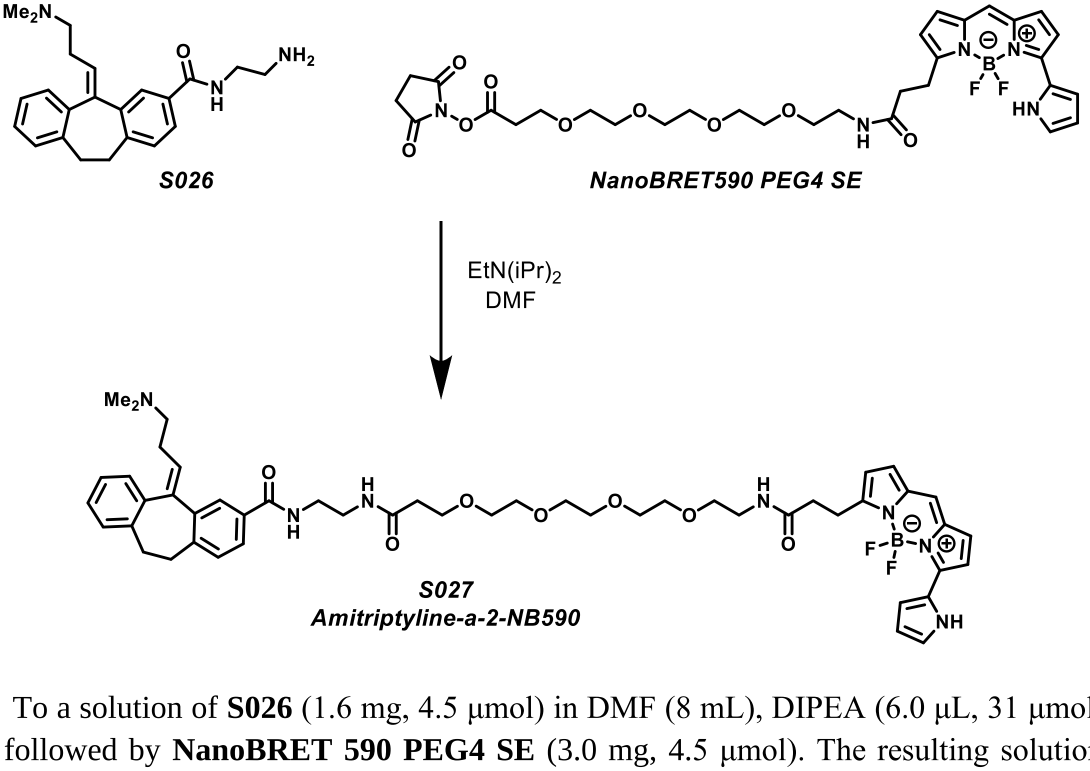

To a solution of **S026** (1.6 mg, 4.5 μmol) in DMF (8 mL), DIPEA (6.0 μL, 31 μmol) was added followed by **NanoBRET 590 PEG4 SE** (3.0 mg, 4.5 μmol). The resulting solution was allowed to react at 22ºC for 24 hours at which point HPLC analysis indicated full consumption of the starting material. Solvent was removed under vacuum, and the crude residue was purified by preparative RP HPLC (5→95% MeCN/H_2O buffered with 0.5% TFA) to provide 1.1 mg (27% yield) of **S027** as a purple film. HPLC: 99% purity at 254 nm; ^{1}H NMR (400 MHz, MeOD) δ 7.78

(d, J = 2.0 Hz, 1H), 7.61 (dd, J = 8.0, 2.0 Hz, 1H), 7.33 – 7.08 (m, 9H), 7.01 (d, J = 4.6 Hz, 1H), 6.91 (d, J = 4.0 Hz, 1H), 6.40 – 6.32 (m, 1H), 6.32 (d, J = 4.0 Hz, 1H), 5.89 (t, J = 7.3 Hz, 1H), 3.66 (t, J = 6.0 Hz, 2H), 3.55 – 3.52 (m, 4H), 3.52 – 3.40 (m, 12H), 3.37 – 3.34 (m, 2H), 3.29 – 3.25 (m, 2H), 3.23 – 3.15 (m, 2H), 2.82 – 2.73 (m, 6H), 2.63 (t, J = 7.7 Hz, 2H), 2.56 (s, 2H), 2.42 (t, J = 6.0 Hz, 2H); HRMS (SI) Calc'd $C_{50}H_{63}BF_2N_7O_7^+$ [M+H]+ 922.4850, found 922.4835.

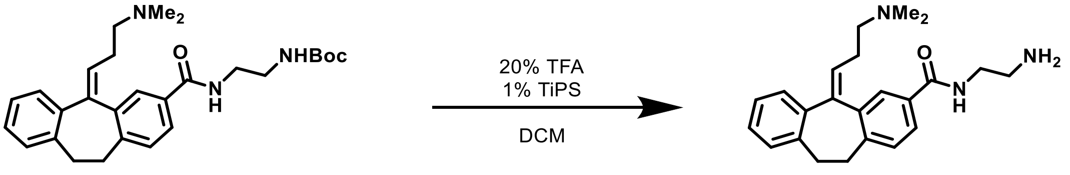

S025 *S028*

A 10 mL flask, equipped with stir bar, was charged with **S025** (4.7 mg, 10.1 µmol), and the cleavage cocktail (4 mL, 80:20:1 DCM/TFA/TIPS). The resulting light-yellow solution was stirred at 22°C for 40 minutes at which point HPLC indicated complete consumption of the staring material and the solvent was removed under reduced pressure. The residue was dissolved in 10 mL MeOH, and solvent removed under reduced pressure. The crude **S028** was used without additional purification in the next step. ^{1}H NMR (400 MHz, MeOD) δ 7.78 (dd, J = 7.9, 1.9 Hz, 1H), 7.68 (d, J = 1.9 Hz, 1H), 7.41 (d, J = 7.9 Hz, 1H), 7.33 – 7.22 (m, 1H), 7.21 – 7.12 (m, 2H), 7.08 (dd, J = 7.2, 1.9 Hz, 1H), 5.88 (t, J = 7.3 Hz, 1H), 3.66 (t, J = 5.9 Hz, 2H), 3.49 – 3.34 (m, 2H), 3.28 – 3.20 (m, 2H), 3.16 (t, J = 6.0 Hz, 2H), 2.98 – 2.87 (m, 2H), 2.81 (m, 6H), 2.70 – 2.49 (m, 2H); MS (ESI) calc'd for $C_{23}H_{30}N_3O_3^+$ $[M+H]^+$ 364.2 found 364.5.

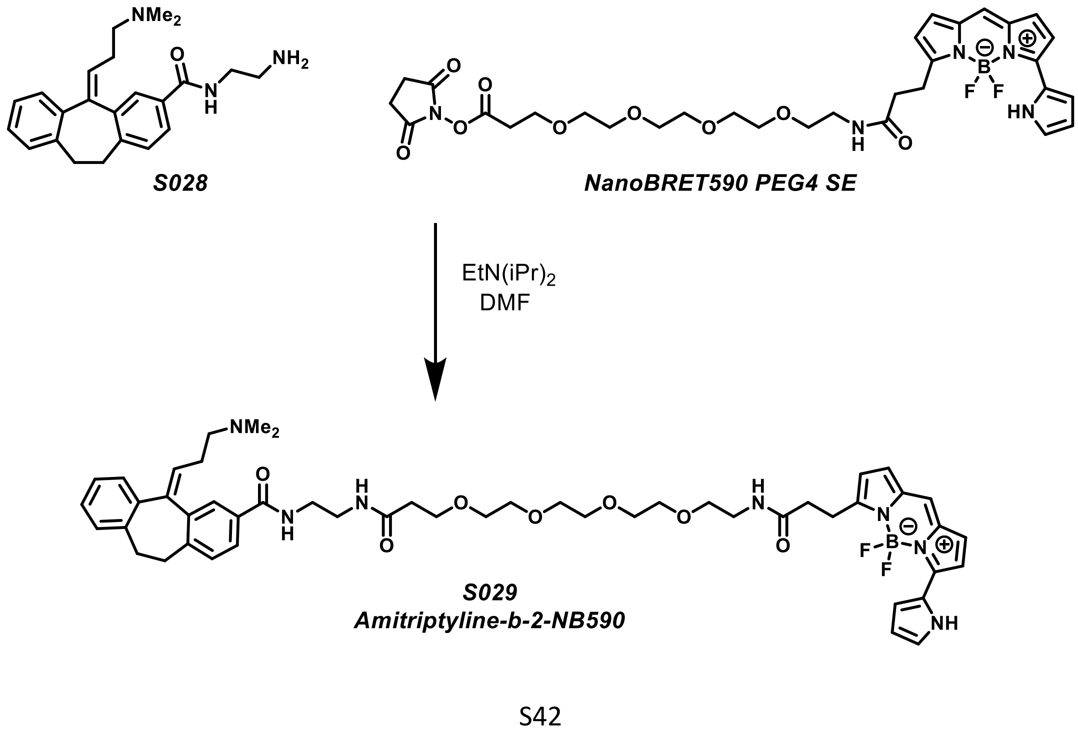

S028 ***NanoBRET590 PEG4 SE***

S029
Amitriptyline-b-2-NB590

To a solution of **S028** (1.0 mg, 3.0 μmol) in DMF (6 mL), DIPEA (4.0 μL, 22 μmol) was added followed by **NanoBRET 590-PEG4 SE** (2.0 mg, 3.0 μmol). The resulting solution was allowed to react at 22ºC for 24 hours at which point HPLC analysis indicated full consumption of the starting material. Solvent was removed under vacuum, and the crude residue was purified by preparative RP HPLC (5→95% MeCN/H_2O buffered with 0.5% TFA) to provide 1.5 mg (55% yield) of **S029** as a purple film. HPLC: 99% purity at 254 nm; ^{1}H NMR (400 MHz, MeOD) δ 7.70 (dd, J = 7.8, 1.9 Hz, 1H), 7.58 (d, J = 1.9 Hz, 1H), 7.36 (d, J = 7.9 Hz, 1H), 7.28 (dd, J = 7.1, 2.0 Hz, 1H), 7.24 – 7.10 (m, 6H), 7.07 (dd, J = 7.1, 2.0 Hz, 1H), 7.01 (d, J = 4.6 Hz, 1H), 6.91 (d, J = 3.9 Hz, 1H), 6.35 (dt, J = 4.1, 2.4 Hz, 1H), 6.32 (d, J = 3.9 Hz, 1H), 5.83 (t, J = 7.3 Hz, 1H), 3.63 (t, J = 5.9 Hz, 2H), 3.53 (s, 4H), 3.51 – 3.46 (m, 4H), 3.45 – 3.36 (m, 10H), 3.36 – 3.33 (m, 4H), 3.28 – 3.16 (m, 4H), 2.97 – 2.84 (m, 2H), 2.81 (s, 6H), 2.63 (t, J = 7.7 Hz, 2H), 2.60 – 2.43 (m, 2H), 2.39 (t, J = 6.0 Hz, 2H); HRMS (SI) Calc'd $C_{50}H_{63}BF_2N_7O_7^+$ [M+H]+ 922.4850, found 922.4871.

Synthesis of Amitriptyline-c and Amitriptyline-d fluorescent tracers

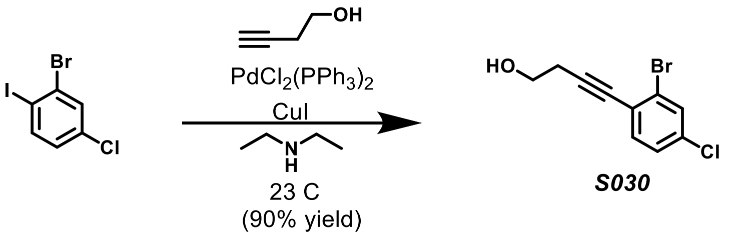

A 50 mL round bottom flask, equipped with stir bar and rubber septum under argon atmosphere, was charged with 2-bromo-4-chloro-1-iodobenzene (2.14 g, 6.74 mmol), CuI (25.7 mg, 0.135 mmol), and $PdCl_2(PPh_3)_2$ (95 mg, 0.13 mmol). Degassed diethylamine (12 mL) was added via syringe followed by 3-butyn-1-ol. The reaction mixture was stirred at 23ºC for 48 hours at which point HPLC indicated complete consumption of the staring material, and the solvent removed under reduced pressure. The crude residue was purified by flash chromatography (gradient elution, 0 → 40% EtOAc/heptane, yielding 1.57 g (90% yield) of alkyne **S030** as a yellow solid. ^{1}H NMR (400 MHz, CDCl3) δ 7.59 (d, J = 2.1 Hz, 1H), 7.37 (d, J = 8.3 Hz, 1H), 7.23 (dd, J = 8.4, 2.1 Hz, 1H), 3.85 (t, J = 6.1 Hz, 2H), 2.74 (t, J = 6.1 Hz, 2H); ^{13}C NMR (100 MHz, $CDCl_3$) δ 134.3, 133.7, 132.1, 127.5, 126.0, 124.0, 92.7, 80.5, 60.9, 24.0; HRMS (ESI) calc'd for $C_{10}H_9BrClO$ $[M+H]^+$ 258.9525 found 258.9521.

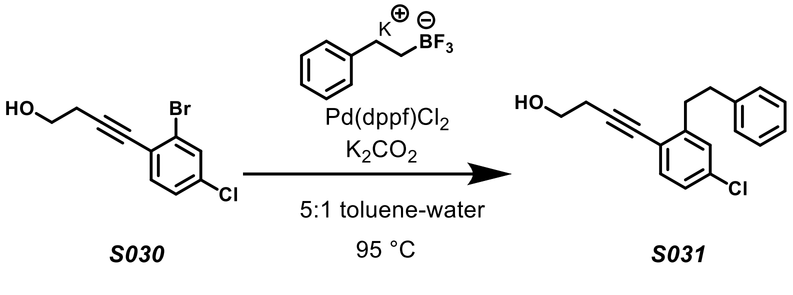

A 500 mL round bottom flask, equipped with stir bar and reflux condenser was charged with **S030** (1.57 g, 6.05 mmol), potassium trifluoro(phenethyl)borate (1.35 g, 6.35 mmol), K_2CO_3 (2.22 g, 18.2 mmol), and Pd(dppf)Cl_2 (220 mg, 0.30 mmol). Flask was evacuated and backfilled with argon (3 x times repeat). Degassed toluene (50 mL) and H_2O (10 mL) were added, and the reaction mixture was stirred at 95ºC for 24 hours. The reaction mixture was cooled to ambient temperature, and solvents were removed under reduces pressure. The crude residue was partitioned between DCM (100 mL) and water (100 mL), aqueous layer was extracted to DCM (2 X 100 mL), organics were combined, dried over $MgSO_4$, filtered, and the solvent removed under reduced pressure. The crude residue was initially purified by flash chromatography (gradient elution, 0 → 100% EtOAc/heptane and then further purified by preparative HPLC (C18, 5 → 95% MeCN/H_2O, 0.05% TFA) yielding 392 mg (23% yield) of alkyne **S031** as a white solid. ^{1}H NMR (400 MHz, CDCl3) δ 7.38 – 7.27 (m, 3H), 7.24 – 7.16 (m, 3H), 7.16 – 7.08 (m, 2H), 3.82 (t, J = 6.3 Hz, 2H), 3.10 – 2.97 (m, 2H), 2.96 – 2.81 (m, 2H), 2.74 (t, J = 6.3 Hz, 2H); ^{13}C NMR (100 MHz, CDCl3) δ 1 13C NMR (101 MHz, CDCl3) δ 145.4, 141.4, 133.7, 133.5, 128.9, 128.4, 128.4, 126.2, 126.1, 121.3, 90.9, 80.0, 61.2, 36.7, 36.7, 24.0; HRMS (ESI) calc'd for $C_{18}H_{18}ClO$ $[M+H]^+$ 285.1046 found 285.1044.

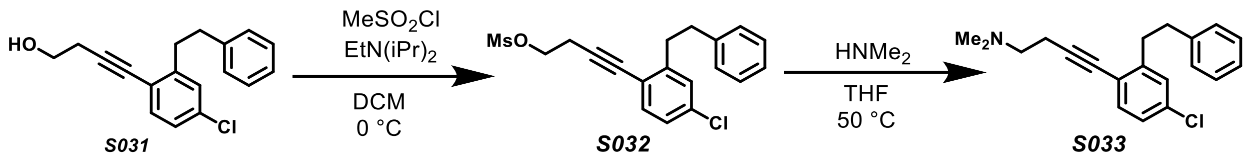

A 50 mL round bottom flask, equipped with stir bar, was charged with **S031** (210 mg, 0.74 mmol), EtN(iPr)$_2$ (263 µL, 1.47 mmol), and DCM (20 mL). The resulting solution was cooled to 0 □C under argon followed by addition of mesyl chloride (86 µL, 1.1 mmol). The reaction mixture was stirred at 0 □C for 4 hours at which point HPLC indicated complete consumption of the staring material. The reaction was quenched by addition of saturated aqueous K_2CO_3 (20 mL), and aqueous phase was further extracted with DCM (3x 20 mL). Organics were combined, dried over MgSO4, filtered, and the solvent removed under reduced pressure. The crude **S032** was used without additional purification in the next step. ^{1}H NMR (400 MHz, CDCl3) δ 7.37 – 7.27 (m, 3H), 7.25 – 7.07 (m, 5H), 4.37 (t, J = 6.8 Hz, 2H), 3.12 – 2.99 (m, 2H), 2.98 (s, 3H), 2.96 – 2.84 (m, 4H).

A 50 mL round bottom flask, equipped with stir bar, was charged with **S032** (270 mg, 0.74 mmol) and 2M dietylamine solution in THF (37 mL, 74 mmol). The reaction mixture was stirred at 50 □C for 17 hours at which point HPLC indicated complete consumption of the staring material, and the solvent removed under reduced pressure. The crude residue was purified by flash chromatography (gradient elution, 0 →20% MeOH/DCM, yielding 125 mg (54% yield) of amine **S033** as a clear oil. ^{1}H NMR (400 MHz, CDCl3) δ 7.39 – 7.27 (m, 3H), 7.24 – 7.18 (m, 3H), 7.16 – 7.03 (m, 2H), 3.08 – 2.95 (m, 2H), 2.95 – 2.83 (m, 2H), 2.63 (s, 4H), 2.31 (s, 6H); ^{13}C NMR (100 MHz, CDCl3) δ 145.4, 141.6, 133.4, 128.8, 128.4, 126.1, 126.0, 121.7, 58.4, 45.1, 36.7, 36.7, 18.5; HRMS (ESI) calc'd for $C_{20}H_{23}ClN$ $[M+H]^+$ 312.1519 found 312.1516.

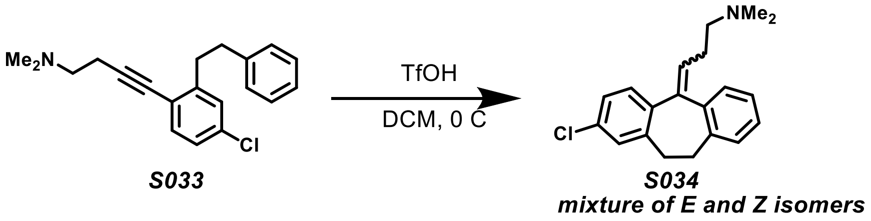

A 50 mL round bottom flask, equipped with stir bar, was charged with a solution of **S033** (120 mg, 0.38 mmol) in DCM (15 mL). The solution was cooled to 0 □C, and triflic acid (170 µL, 1.9 mmol) was added in one portion. The resulting brown solution is stirred at 0ºC for 10 minutes at which point the reaction was quenched by addition of saturated aqueous solution of K_2CO_3 (15 mL). Organic layer was separated, and the aqueous solution was extracted (2x 15 mL DCM). Organics were combined, dried over MgSO4, filtered, and concentrated *in vacuo*. The crude residue was purified by flash chromatography (gradient elution, 0 →30% MeOH/DCM, yielding 101 mg (99% yield) of amine **S034** as a clear oil. ^{1}H NMR (400 MHz, CDCl3, reported for mixture of E/Z isomers) δ 7.27 – 7.23 (m, 1H), 7.23 – 6.98 (m, 6H), 5.86 (m, 1H), 3.65 – 3.12 (m, 2H), 2.94 (s, 1H), 2.75 (s, 1H), 2.50 – 2.34 (m, 2H), 2.29 (m, 2H), 2.19 (s, 6H); ^{13}C NMR (100 MHz, CDCl3, reported for mixture of E/Z isomers) δ 142.6, 142.5, 141.2, 140.8, 139.7, 139.6, 139.0, 138.8, 138.5, 136.7, 132.8, 132.5, 130.0, 129.9, 129.8, 129.6, 129.6, 128.6, 128.1, 128.0, 127.6, 127.2, 126.1, 126.0, 125.9, 125.8, 59.2, 45.2, 45.2, 33.7, 33.4, 31.9, 31.7, 27.8, 27.7. HRMS (ESI) calc'd for $C_{10}H_{23}ClN$ $[M+H]^+$ 312.1519 found 312.1510. Single peak on HPLC at 254 nm.

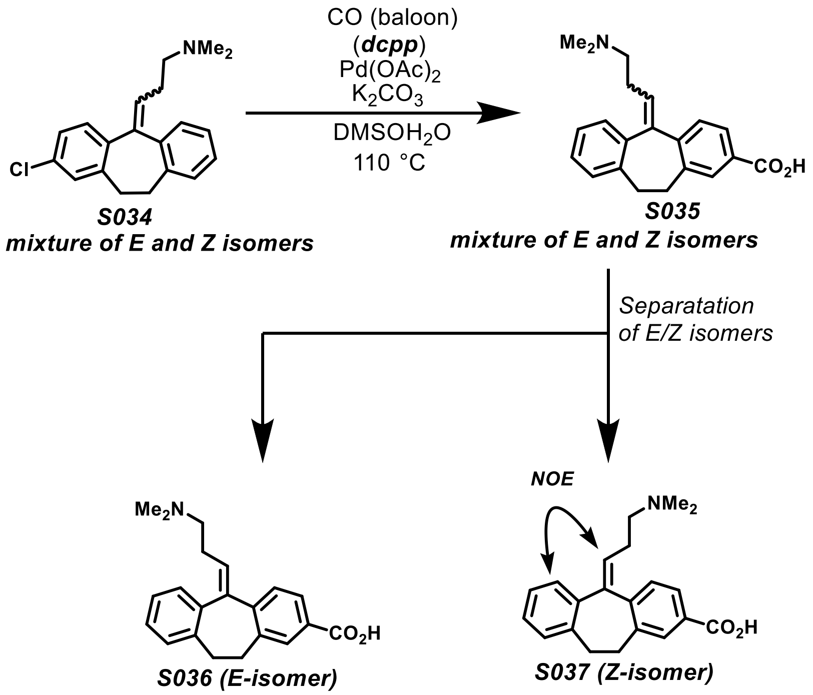

A 50 mL round bottom flask, equipped with stir bar and septum, was charged with **S034** (100 mg, 0.32 mmol), K_2CO_3 (130 mg, 0.96 mmol), $Pd(OAc)_2$ (7.2 mg, 32 µmol), and [dcpp $2BF_4$]

(39 mg, 64 µmol). Flask was evacuated and backfilled with argon (3x times repeated). Degassed DMSO (8 mL) and H_2O (0.8 mL) were added, and reaction vessel evacuated and backfilled with carbon monoxide (3 x times repeat). CO was allowed to bubble through the solution for 5 minutes. The resulting yellow suspension was heated to 110°C under CO balloon for 22 hours at which point HPLC analysis indicated complete consumption of the starting material. The reaction mixture was diluted with MeOH (8 mL), passed through syringe filter, and purified by preparative HPLC (C18, 5 → 95% MeCN/H_2O, 0.05% TFA) yielding 54 mg (51% yield) of **S036** as a clear oil and 51 mg (48% yield) of **S037** as a clear oil. **S036 (**E isomer) has shorter retention time than **S037** (Z isomer).

Characterization data for **S036**: ^{1}H NMR (400 MHz, MeOD) δ 7.82 (dd, J = 8.0, 1.8 Hz, 1H), 7.78 (d, J = 1.8 Hz, 1H), 7.43 (d, J = 8.0 Hz, 1H), 7.36 – 7.24 (m, 3H), 7.23 – 7.16 (m, 1H), 5.93 (t, J = 7.3 Hz, 1H), 3.39 (t, J = 9.0 Hz, 2H), 3.26 (q, J = 7.4 Hz, 2H), 3.02 (d, J = 14.9 Hz, 1H), 2.82 (d, J = 8.1 Hz, 7H), 2.60 (dd, J = 16.9, 8.3 Hz, 2H); ^{13}C NMR (100 MHz, MeOD) δ 169.6, 147.7, 146.1, 140.6, 139.7, 138.7, 132.8, 131.1, 129.7, 129.6, 129.6, 129.1, 128.5, 127.4, 126.5, 58.0, 34.8, 32.7, 26.2; HRMS (ESI) calc'd for $C_{21}H_{24}NO_2$ $[M+H]^+$ 322.1807 found 322.1807.

Characterization data for **S037**: ^{1}H NMR (400 MHz, MeOD) δ 7.97 (d, J = 1.7 Hz, 1H), 7.92 (dd, J = 7.8, 1.8 Hz, 1H), 7.37 – 7.25 (m, 2H), 7.25 – 7.13 (m, 2H), 7.10 (dd, J = 7.2, 1.9 Hz, 1H), 5.89 (t, J = 7.3 Hz, 1H), 3.55 – 3.36 (m, 2H), 3.26 (q, J = 8.6 Hz, 2H), 2.97 (d, J = 25.6 Hz, 2H), 2.82 (d, J = 6.3 Hz, 6H), 2.68 – 2.45 (m, 2H); ^{13}C NMR (100 MHz, MeOD) δ 169.5, 147.6, 145.4, 141.2, 140.8, 138.2, 131.7, 131.3, 130.7, 129.5, 129.1, 129.0, 128.8, 127.4, 125.9, 58.0, 34.5, 32.8, 26.2; HRMS (ESI) calc'd for $C_{21}H_{24}NO_2$ $[M+H]^+$ 322.1807 found 322.1807.

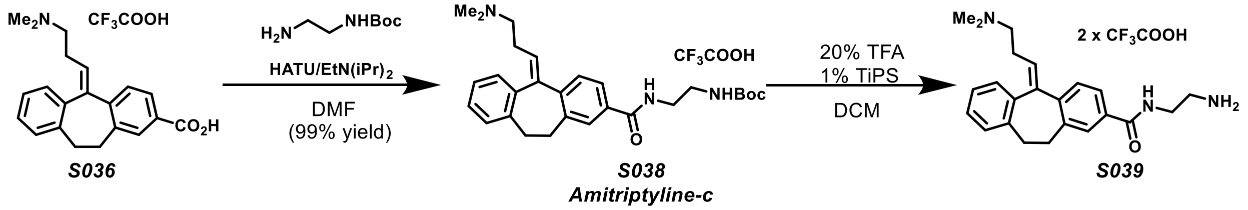

A 25 mL flask, equipped with stir bar, was charged with **S036** (36 mg, 83 µmol), tert-butyl (2-aminoethyl) carbamate (39 mg, 103 µmol), HATU (17 mg, 0.10 mmol), $EtN(iPr)_2$ (74 µL, 0.41 mmol), and DMF (8 mL). The resulting light-yellow solution was stirred at 22°C for 18 hours at which point HPLC indicated complete consumption of the staring material, and the solvent was removed under reduced pressure. The crude residue was purified by preparative HPLC (C18, 5 → 95% MeCN/H_2O, 0.05% TFA), yielding 47 mg (99% yield) of amide **S038** as a clear oil. MS (ESI) calc'd for $C_{28}H_{38}NO_3$ $[M+H]^+$ 464.3 found 464.4. Single peak on HPLC at 254 nm.

A 10 mL flask, equipped with stir bar, was charged with **S038** (20 mg, 35 µmol), and the cleavage cocktail (4 mL, 80:20:1 DCM/TFA/TIPS). The resulting light-yellow solution was stirred at 22°C for 75 minutes at which point HPLC indicated complete consumption of the staring

material, and the solvent was removed under reduced pressure. The residue was dissolved in 10 mL MeOH, and solvent removed under reduced pressure. The crude **S039** was used without additional purification in the next step. MS (ESI) calc'd for $C_{23}H_{30}N_3O_3^+$ $[M+H]^+$ 364.2 found 364.3. Single peak on HPLC at 254 nm.

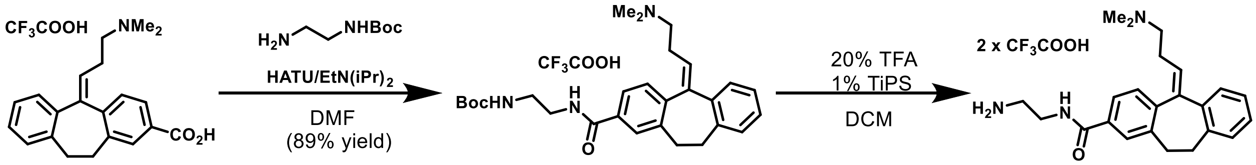

S037 ***S040*** ***Amitriptyline-d*** ***S041***

A 25 mL flask, equipped with stir bar, was charged with **S037** (26 mg, 81 μmol), tert-butyl (2-aminoethyl) carbamate (39 mg, 0.10 mmol), HATU (16 mg, 0.10 mmol), $EtN(iPr)_2$ (72 μL, 0.40 mmol), and DMF (8 mL). The resulting light-yellow solution was stirred at 22°C for 17 hours at which point HPLC indicated complete consumption of the staring material, and the solvent was removed under reduced pressure. The crude residue was purified by preparative HPLC (C18, 5 → 95% MeCN/H2O, 0.05% TFA), yielding 33 mg (89% yield) of amide **S040** as a clear oil. MS (ESI) calc'd for $C_{28}H_{38}NO_3$ $[M+H]^+$ 464.3 found 464.4. Single peak on HPLC at 254 nm.

A 10 mL flask, equipped with stir bar, was charged with **S040** (15 mg, 35 μmol), and the cleavage cocktail (4 mL, 80:20:1 DCM/TFA/TIPS). The resulting light-yellow solution was stirred at 22°C for 85 minutes at which point HPLC indicated complete consumption of the staring material, and the solvent removed under reduced pressure. The residue was dissolved in 10 mL MeOH, and solvent removed under reduced pressure. The crude **S041** was used without additional purification in the next step. MS (ESI) calc'd for $C_{23}H_{30}N_3O_3^+$ $[M+H]^+$ 364.2 found 364.4; Single peak on HPLC at 254 nm.

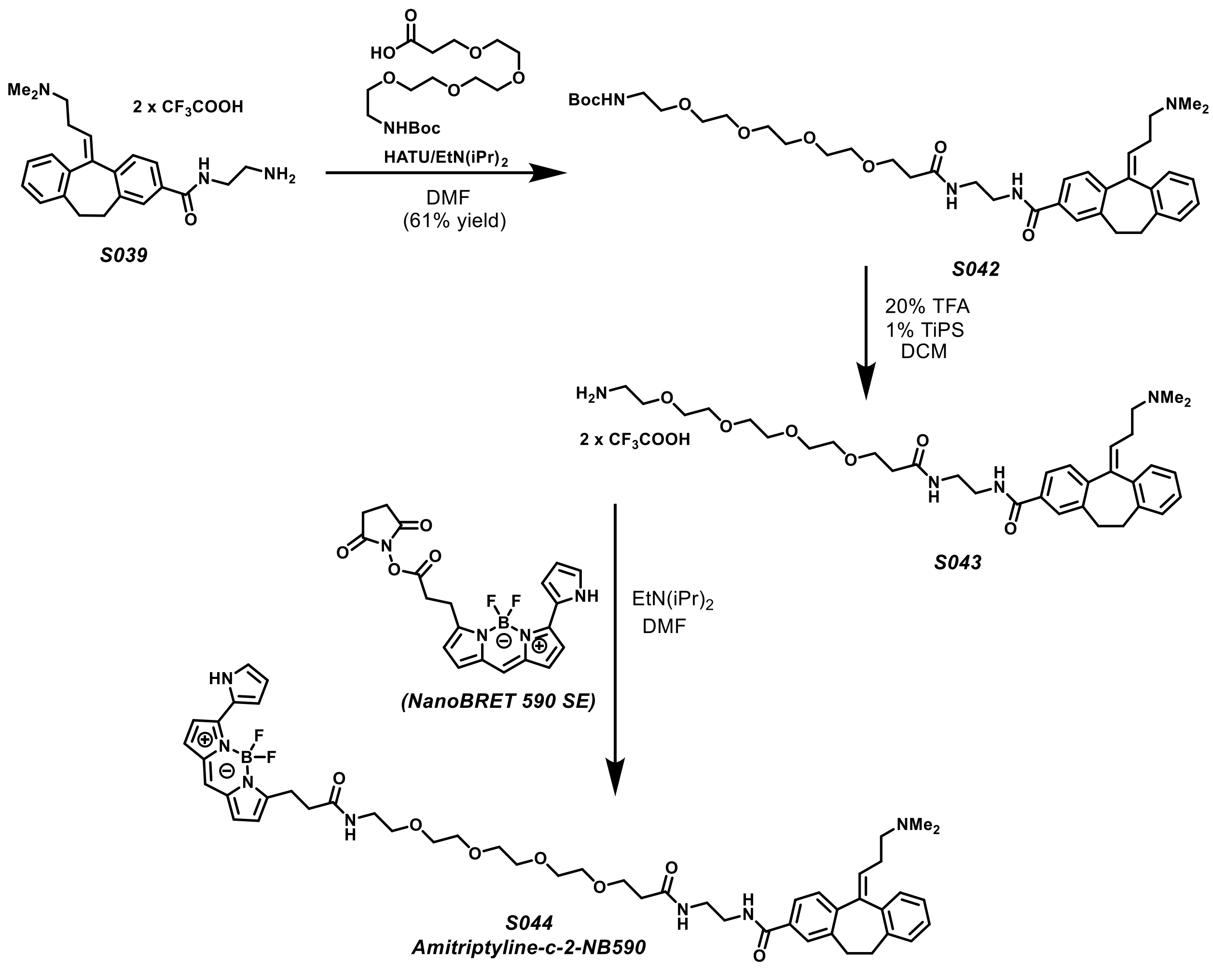

A 25 mL flask, equipped with stir bar, was charged with **S039** (5.2 mg, 8.8 μmol), 2,2-dimethyl-4-oxo-3,8,11,14,17-pentaoxa-5-azaicosan-20-oic acid (4.0 mg, 11 μmol), HATU (4.2 mg, 11 μmol), EtN(iPr)$_2$ (11 μL, 62 μmol), and DMF (6 mL). The resulting light-yellow solution was stirred at 22°C for 17 hours at which point, HPLC indicated complete consumption of the staring material and the solvent was removed under reduced pressure. The crude residue was purified by preparative HPLC (C18, 5 → 95% MeCN/H2O, 0.05% TFA), yielding 3.8 mg (61% yield) of amide **S042** as a clear oil. 1H NMR (400 MHz, MeOD) δ 7.64 (dd, J = 8.0, 1.9 Hz, 1H), 7.59 (d, J = 1.9 Hz, 1H), 7.42 (d, J = 8.0 Hz, 1H), 7.37 – 7.23 (m, 3H), 7.20 (dd, J = 6.8, 1.6 Hz, 1H), 5.92 (t, J = 7.3 Hz, 1H), 3.71 (t, J = 6.0 Hz, 2H), 3.59 – 3.37 (m, 20H), 3.29 – 3.13 (m, 4H), 3.03 (q, J = 15.7, 14.5 Hz, 1H), 2.82 (m, 7H), 2.61 (t, J = 9.2 Hz, 2H), 2.45 (t, J = 6.0 Hz, 2H), 1.44 (s, 9H); HRMS (ESI) calc'd for $C_{39}H_{59}N_4O_8$ $[M+H]^+$ 711.4333 found 711.4329. Single peak on HPLC at 254 nm.

A 10 mL flask, equipped with stir bar, was charged with **S042** (3.8 mg, 4.6 μmol), and the cleavage cocktail (4 mL, 80:20:1 DCM/TFA/TIPS). The resulting light-yellow solution was stirred at 22°C for 60 minutes at which point HPLC indicated complete consumption of the staring

material, and the solvent removed under reduced pressure. The residue was dissolved in 10 mL MeOH, and solvent removed under reduced pressure. The crude **S043** was used without additional purification in the next step. MS (ESI) calc'd for $C_{34}H_{52}N_4O_6^{2+}$ $[M+H]^{2+}/2$ 306.2 found 306.4; Single peak on HPLC at 254 nm.

To a solution of **S043** (4 mg, 5 µmol) in DMF (6 mL) was added DIPEA (6.0 µL, 33 µmol) followed by **NanoBRET590 SE** (2.0 mg, 4.7 µmol). The resulting solution was allowed to react at 22ºC for 23 hours at which point HPLC analysis indicated full consumption of the starting material. Solvent was removed under vacuum, and the crude residue was purified by preparative RP HPLC (5→95% MeCN/H_2O buffered with 0.5% TFA) to provide 3.0 mg (70% yield) of **S044** as a purple film. HPLC: 99% purity at 254 nm; ^1H NMR (400 MHz, MeOD) δ 7.60 (dd, J = 8.1, 1.9 Hz, 1H), 7.55 (d, J = 1.9 Hz, 1H), 7.37 (d, J = 8.0 Hz, 1H), 7.28 (td, J = 4.5, 4.1, 2.7 Hz, 2H), 7.26 – 7.17 (m, 5H), 7.17 – 7.10 (m, 1H), 7.01 (d, J = 4.6 Hz, 1H), 6.91 (d, J = 4.0 Hz, 1H), 6.34 (t, J = 3.1 Hz, 1H), 6.31 (d, J = 4.0 Hz, 1H), 5.86 (t, J = 7.3 Hz, 1H), 3.65 (t, J = 6.0 Hz, 2H), 3.56 – 3.43 (m, 16H), 3.41 (d, J = 5.6 Hz, 2H), 3.35 (m, 4H), 3.27 (d, J = 7.7 Hz, 2H), 3.19 (s, 2H), 2.97 (s, 1H), 2.77 (d, J = 13.9 Hz, 7H), 2.63 (t, J = 7.7 Hz, 2H), 2.55 (t, J = 8.8 Hz, 2H), 2.40 (t, J = 6.0 Hz, 2H); HRMS (SI) Calc'd $C_{50}H_{63}BF_2N_7O_7^+$ [M+H]+ 922.4850, found 922.4847.

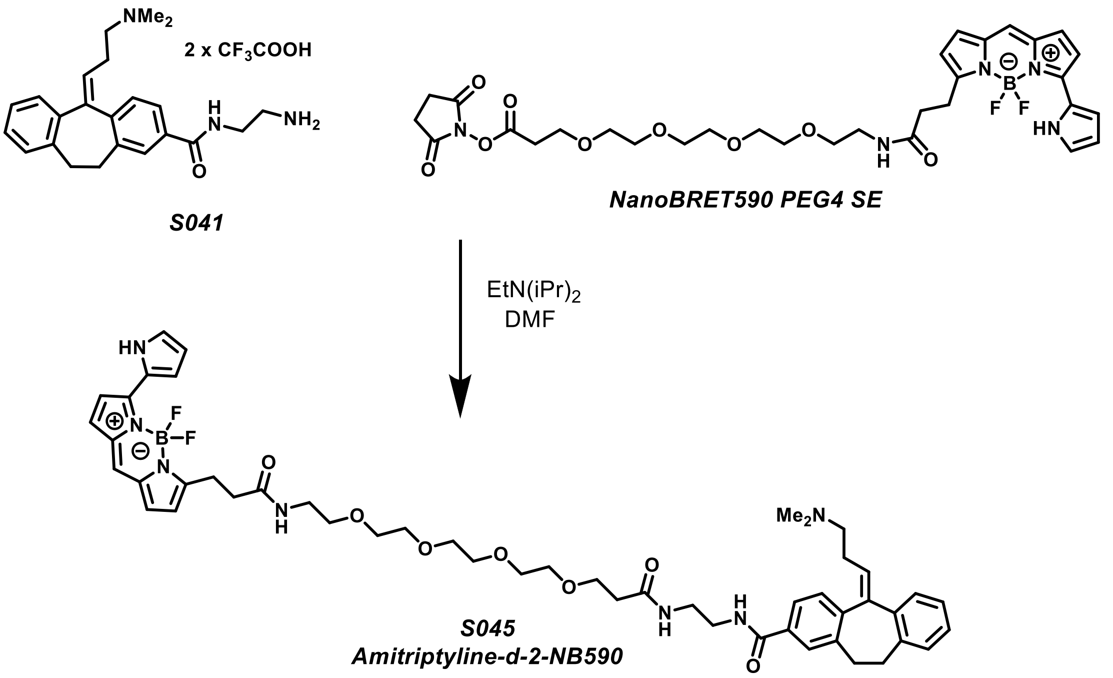

To a solution of **S041** (8.0 mg, 14 µmol) in DMF (6 mL), DIPEA (12 µL, 68 µmol) was added followed by **NanoBRET 590-PEG4 SE** (5.5 mg, 8.1 µmol). The resulting solution was allowed to react at 22ºC for 2 hours at which point HPLC analysis indicated full consumption of the starting material. Solvent was removed under vacuum, and the crude residue was purified by preparative RP HPLC (5→95% MeCN/H_2O buffered with 0.5% TFA) to provide 2.3 mg (19%

yield) of **S045** as a purple film. HPLC: 99% purity at 254 nm; ^{1}H NMR (400 MHz, MeOD) δ 7.74 (d, J = 1.9 Hz, 1H), 7.68 (dd, J = 7.8, 1.9 Hz, 1H), 7.38 – 7.11 (m, 8H), 7.07 (dd, J = 7.2, 1.9 Hz, 1H), 7.01 (d, J = 4.5 Hz, 1H), 6.91 (d, J = 4.0 Hz, 1H), 6.35 (dt, J = 4.0, 2.3 Hz, 1H), 6.32 (d, J = 4.0 Hz, 1H), 5.83 (t, J = 7.3 Hz, 1H), 3.66 (t, J = 6.0 Hz, 2H), 3.53 (s, 4H), 3.50 – 3.45 (m, 10H), 3.45 – 3.38 (m, 2H), 3.35 (d, J = 5.4 Hz, 2H), 3.26 (d, J = 7.7 Hz, 2H), 3.23 – 3.14 (m, 2H), 2.97 – 2.87 (m, 2H), 2.78 (d, J = 14.9 Hz, 6H), 2.63 (t, J = 7.7 Hz, 2H), 2.53 (t, J = 14.3 Hz, 2H), 2.41 (t, J = 6.0 Hz, 2H); HRMS (SI) Calc'd $C_{50}H_{63}BF_2N_7O_7^+$ [M+H]+ 922.4850, found 922.4859.

Synthesis of Amitriptyline-e

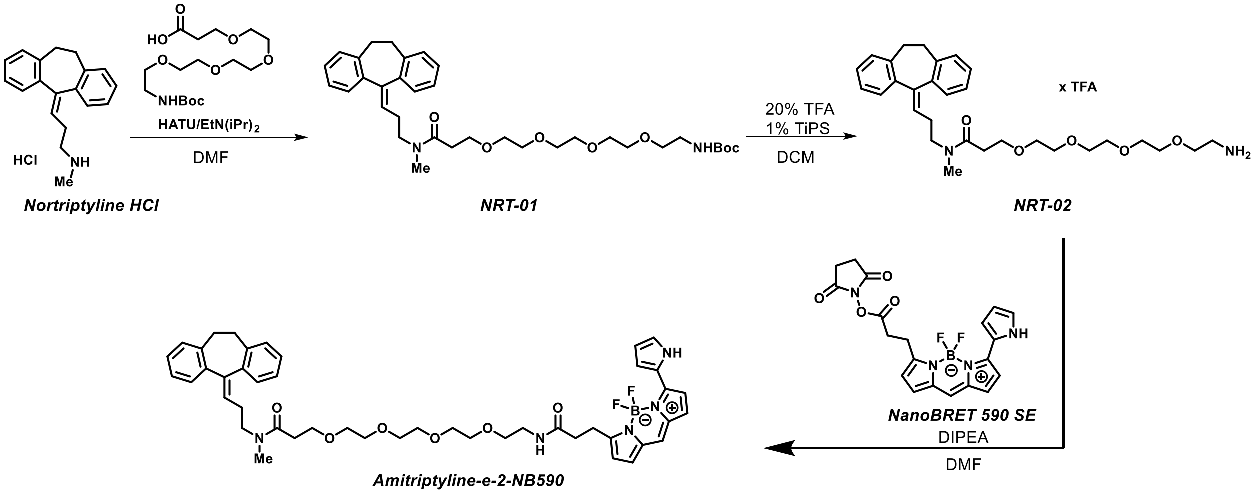

A 25 mL flask, equipped with stir bar, was charged with **Nortiptyline HCl** (24 mg, 80 µmol), 2,2-dimethyl-4-oxo-3,8,11,14,17-pentaoxa-5-azaicosan-20-oic acid (37 mg, 100 µmol), HATU (38 mg, 100 µmol), $EtN(iPr)_2$ (100 µL, 570 µmol), and DMF (8 mL). The resulting light-yellow solution was stirred at 22°C for 6 hours at which point, HPLC indicated complete consumption of the staring material and the solvent was removed under reduced pressure. The crude residue was purified by preparative HPLC (C18, 5 → 95% MeCN/H2O, 0.05% TFA), yielding 31 mg (63% yield) of amide **NRT-01** as a clear residue. ^{1}H NMR (400 MHz, DMSO-d_6) δ 7.38 – 7.06 (m, 7H), 7.07 (ddt, J = 7.8, 6.0, 2.7 Hz, 2H), 6.74 (s, 1H), 5.81 (dt, J = 32.6, 7.5 Hz, 1H), 3.58 (t, J = 6.8 Hz, 1H), 3.53 (t, J = 6.8 Hz, 1H), 3.49 – 3.42 (m, 10H), 3.42 – 3.33 (m, 5H), 3.05 (q, J = 6.0 Hz, 2H), 2.85 (s, 3H), 2.65 (s, 4H), 1.36 (s, 9H); HRMS (ESI) calc'd for $C_{35}H_{51}N_2O_7$ [M+H]$^+$ 611.3691 found 611.3699.

A 25 mL flask, equipped with stir bar, was charged with **NRT-01** (30 mg, 49 µmol), and cleavage cocktail (10 mL, 80:20:1 DCM/TFA/TIPS). The resulting light-yellow solution was stirred at 22°C for 60 minutes at which point HPLC indicated complete consumption of the staring material, and the solvent removed under reduced pressure. The residue was dissolved in 10 mL MeOH, and solvent removed under reduced pressure. The crude **NRT-02** was used without

additional purification in the next step. MS (ESI) calc'd for $C_{30}H_{43}N_2O_5^+$ $[M+H]^+$ 511.3 found 511.3; Single peak on HPLC at 254 nm.

To a solution of **NRT-02** (5.5 mg, 11 µmol) in DMF (5 mL) was added DIPEA (11 µL, 61 µmol) followed by **NanoBRET590 SE** (3.7 mg, 8.7 µmol). The resulting solution was allowed to react at 22ºC for 23 hours at which point HPLC analysis indicated full consumption of the starting material. Solvent was removed under vacuum, and the crude residue was purified by preparative RP HPLC (5→95% MeCN/H_2O buffered with 0.5% TFA) to provide 3.8 mg (53% yield) of **Amitriptyline-e-2-NB590** as a purple film. HPLC: 99% purity at 254 nm; ^{1}H NMR (400 MHz, MeOD) δ 7.30 – 7.13 (m, 8H), 7.13 – 7.04 (m, 3H), 7.01 (dd, J = 6.5, 3.8 Hz, 2H), 6.91 (d, J = 4.0 Hz, 1H), 6.43 – 6.25 (m, 2H), 5.80 (q, J = 7.6 Hz, 1H), 3.67 (t, J = 6.4 Hz, 1H), 3.60 – 3.45 (m, 15H), 3.43 (td, J = 4.3, 1.1 Hz, 2H), 3.40 – 3.33 (m, 3H), 3.28 (d, J = 7.8 Hz, 3H), 2.88 (s, 3H), 2.71 (s, 3H), 2.67 – 2.60 (m, 2H), 2.55 (t, J = 6.4 Hz, 1H), 2.51 – 2.35 (m, 2H), 2.31 (q, J = 7.0 Hz, 1H); HRMS (SI) Calc'd $C_{46}H_{55}BF_2N_5O_6^+$ [M+H]+ 822.4208, found 822.4213.

Synthesis of AZD1283 fluorescent tracer

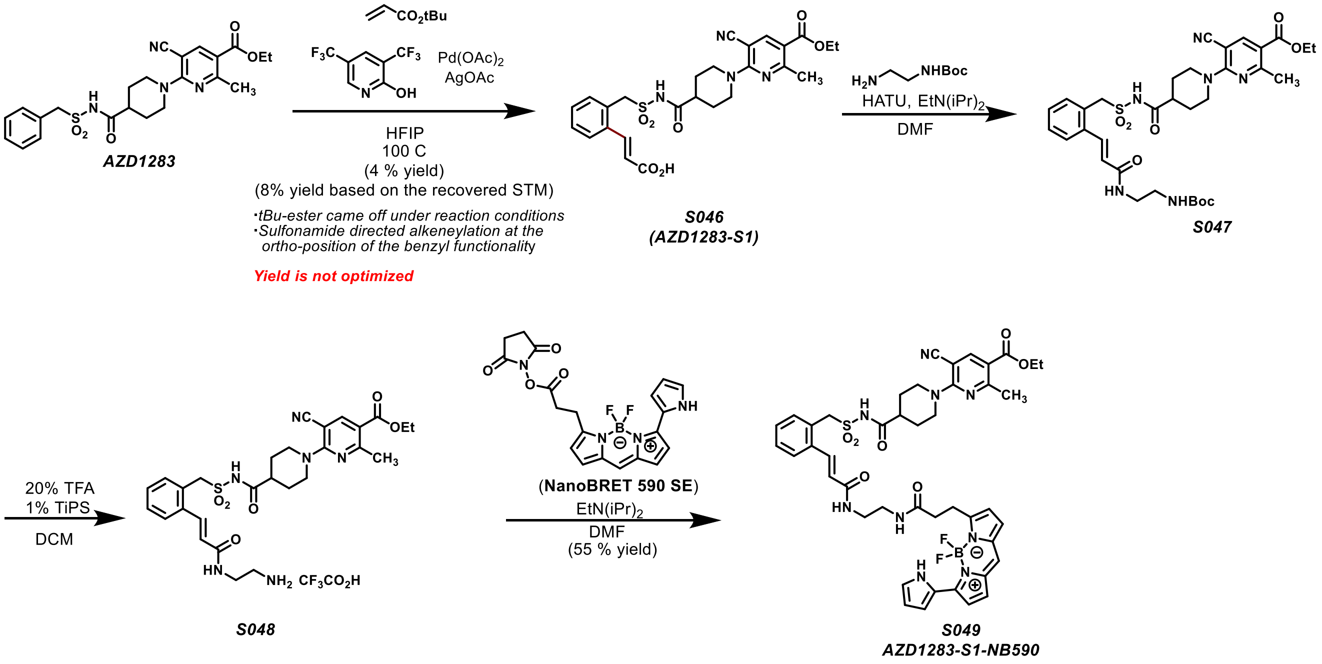

AZD1283 (42 mg, 89 µmol), tert-butyl acrylate (26 µL, 180 µmol), 3,5-bis(trifluoromethyl)pyridin-2-ol (6.2 mg, 27 µmol), AgOAc (45 mg, 270 µmol), $Pd(OAc)_2$ (2 mg, 9 µmol) are places in 1 drum vial equipped with a stir bar, followed by 0.5 mL HFIP. The vial was capped with a screw cap and the stirred reaction mixture was heated to 100 °C for 22 hours. The crude residue was purified by preparative HPLC (C18, 5 → 95% MeCN/H_2O, 0.05% TFA) to provide 2 mg (4% yield) of **S046** as a clear oil. ^{1}H NMR (400 MHz, CD_3CN) δ 9.20 (s, 1H), 8.34

(s, 1H), 8.02 (d, J = 15.8 Hz, 1H), 7.85 – 7.69 (m, 1H), 7.46 (dt, J = 7.5, 3.9 Hz, 2H), 7.42 – 7.29 (m, 1H), 6.45 (d, J = 15.8 Hz, 1H), 4.82 (s, 2H), 4.64 (dt, J = 13.6, 3.8 Hz, 2H), 4.28 (q, J = 7.1 Hz, 2H), 3.25 – 3.06 (m, 2H), 2.67 (s, 3H), 2.59 (s, 2H), 1.76 – 1.66 (m, 3H), 1.33 (t, J = 7.1 Hz, 3H); HRMS (ESI+) calc'd for $C_{26}H_{27}N_4O_7S$ $[M-H]^-$ 539.1600, found 539.1602.

To a solution of **S046** (2 mg, 4 µmol), HATU (2 mg, 5 µmol) and DIPEA (1 µL, 7 µmol) in DMF (5 mL) was added Boc-ethylenediamine (3 mg, 18 µmol). The resulting solution was stirred at 22 °C for 18 hours, at which point LCMS analysis indicated full consumption of starting material. The reaction mixture was purified by preparative RP HPLC (5→95% MeCN/H_2O buffered with 0.5% TFA) to provide 2 mg (80% yield) of **S047** as a clear oil. ^{1}H NMR (400 MHz, CD_3CN) δ 8.34 (s, 1H), 7.85 (d, J = 15.5 Hz, 1H), 7.71 (dd, J = 7.3, 1.9 Hz, 1H), 7.41 (dtd, J = 21.0, 7.2, 2.0 Hz, 3H), 6.85 (s, 1H), 6.49 (d, J = 15.5 Hz, 1H), 5.53 (s, 1H), 4.82 (s, 2H), 4.64 (dt, J = 13.5, 3.8 Hz, 2H), 4.28 (q, J = 7.1 Hz, 2H), 3.32 (q, J = 5.9 Hz, 2H), 3.25 – 3.06 (m, 5H), 2.67 (s, 4H), 2.61 (s, 2H), 1.39 (s, 11H), 1.33 (t, J = 7.1 Hz, 4H); HRMS (ESI+) calc'd for $C_{33}H_{41}N_6O_8S$ $[M-H]^-$ 681.2712, found 681.2712.

To a solution of **S047** (2 mg, 3 µmol) in DCM (5 mL) was added TiPS (50 µL) followed by TFA (1 mL). The resulting solution was stirred at 22 °C for 1 hour, at which point HPLC analysis indicated full consumption of starting material. Volatiles were removed under reduced pressure and crude **S048** was dissolved in 5 mL MeOH and volatiles removed under vacuum. The crude residue was dried under high vacuum and used in the next step without further purification. MS (ESI+) calc'd for $C_{28}H_{35}N_6O_6S$ $[M+H]^+$ 583.2, found 583.5.

To a solution of **S048** (2.0 mg, 2.9 µmol) and DIPEA (3 µL, 16 µmol) in DMF (6 mL) was added **NanoBRET590 SE** (1.2 mg, 2.9 µmol). The resulting solution was allowed to react at 22 °C for 3 hours at which point HPLC analysis indicated full consumption of the starting material. Solvent was removed under vacuum and the crude residue was purified by preparative HPLC (C18, 5 → 95% MeCN/H_2O, 0.05% TFA) to provide 1.4 mg (55% yield) of **S049** as a purple film. ^{1}H NMR (400 MHz, MeOD) δ 10.69 (s, 1H), 8.33 (s, 1H), 7.91 (d, J = 15.7 Hz, 1H), 7.78 – 7.57 (m, 1H), 7.50 – 7.31 (m, 3H), 7.22 – 7.15 (m, 2H), 7.13 (d, J = 4.6 Hz, 1H), 6.97 (d, J = 4.6 Hz, 1H), 6.88 (d, J = 4.0 Hz, 1H), 6.49 (d, J = 15.6 Hz, 1H), 6.33 (d, J = 3.2 Hz, 1H), 6.31 (d, J = 4.0 Hz, 1H), 4.65 (d, J = 13.5 Hz, 2H), 4.30 (q, J = 7.1 Hz, 2H), 3.44 – 3.38 (m, 4H), 3.27 (d, J = 7.6 Hz, 2H), 3.15 (s, 2H), 2.67 (d, J = 8.9 Hz, 5H), 2.59 – 2.48 (m, 1H), 1.88 (d, J = 12.4 Hz, 2H), 1.82 – 1.66 (m, 2H), 1.36 (td, J = 7.1, 4.8 Hz, 3H); HRMS (ESI+) calc'd for $C_{44}H_{47}BF_2N_9O_7S$ $[M+H]^+$ 894.3375, found 894.3381.

Synthesis of Azamelatonin fluorescent tracers

Synthesis of Azamelatonin-1 tracer (N-attachment)

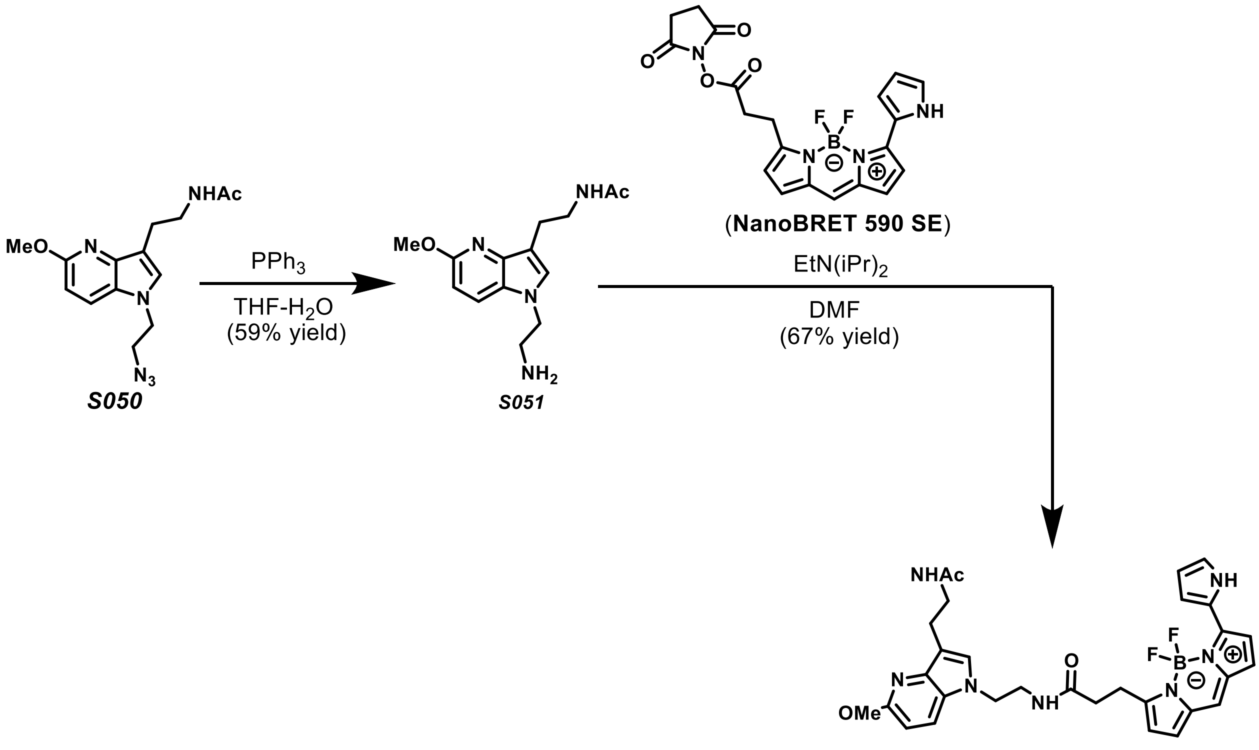

S052
Azamelatonin-1-NB590

A solution of **S050 [6]** (87 mg, 0.29 mmol) in THF (5 mL) was added PPh_3 (110 mg, 0.43 mmol) and water (30 µL). The resulting solution was stirred at 22 °C for 22 hours. Solvent was removed under vacuum and the residue was purified by silica gel chromatography (0→100% MeOH/DCM) to provide 47 mg (59% yield) of **S051** as a light-yellow oil ^{1}H NMR (400 MHz, MeOD) δ 7.59 (d, J = 8.8 Hz, 1H), 7.32 (s, 1H), 7.10 (s, 1H), 6.63 (d, J = 8.8 Hz, 1H), 4.11 (t, J = 5.9 Hz, 2H), 4.03 (s, 3H), 3.56 (q, J = 5.4 Hz, 2H), 3.10 (d, J = 8.1 Hz, 2H), 3.03 – 2.82 (m, 2H), 1.93 (s, 3H); MS (ESI+) calc'd for $C_{14}H_{21}N_4O_2$ $[M+H]^+$ 277.2, found 277.4.

To a solution of **S051** (1.2 mg, 3.1 µmol) and DIPEA (3 µL, 16 µmol) in MeCN (5 mL) was added NanoBRET590 SE (1.3 mg, 3.1 µmol). The resulting solution was allowed to react at 22 °C for 4 hours at which point HPLC analysis indicated full consumption of the starting material. Solvent was removed under vacuum and the crude residue was purified by preparative HPLC (C18, 5 → 95% MeCN/H_2O, 0.05% TFA) to provide 1.2 mg (67% yield) of **052** as a purple film. ^{1}H NMR (400 MHz, MeOD) δ 8.12 (d, J = 9.0 Hz, 1H), 7.47 (s, 1H), 7.29 – 7.16 (m, 4H), 7.04 (d, J = 4.5 Hz, 1H), 6.92 – 6.81 (m, 2H), 6.38 (q, J = 2.7 Hz, 1H), 6.22 (d, J = 4.0 Hz, 1H), 4.31 (t, J = 5.7 Hz, 2H), 4.00 (s, 3H), 3.59 (q, J = 5.8 Hz, 2H), 3.43 (t, J = 7.1 Hz, 2H), 3.10 (d, J = 7.7 Hz,

2H), 2.88 (t, J = 7.1 Hz, 2H), 2.49 (t, J = 7.7 Hz, 2H), 1.90 (s, 3H); HRMS (ESI+) calc'd for $C_{30}H_{33}BF_2N_7O_3$ $[M+H]^+$ 588.2706, found 588.2695.

Synthesis of Azamelatonin-2 ICOA9 tracer

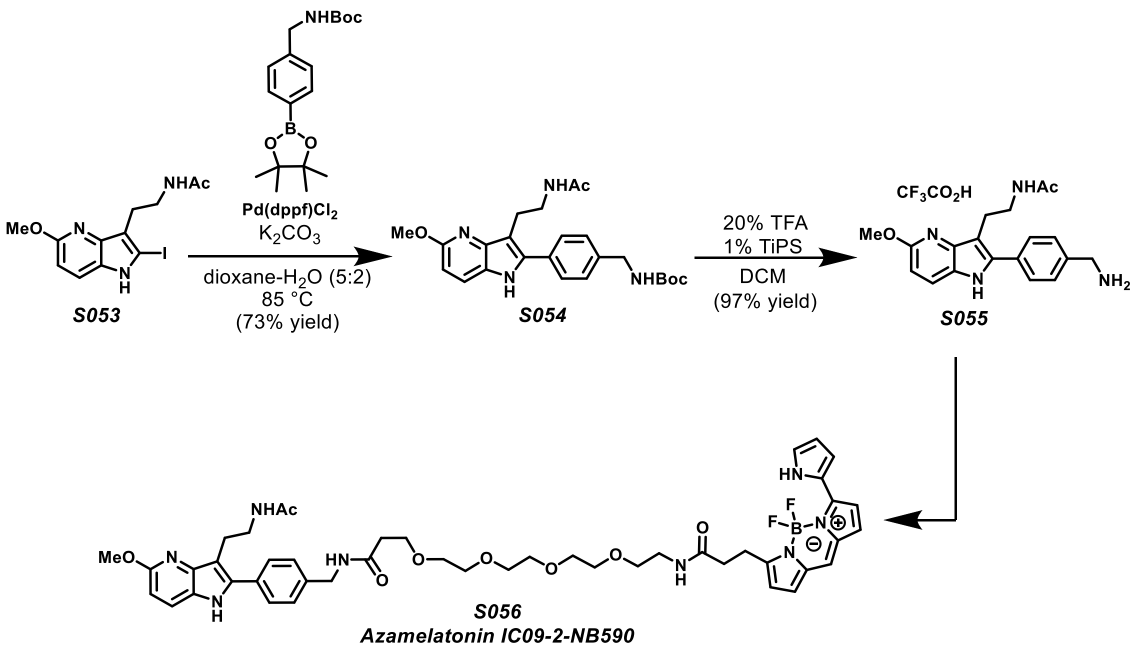

A 40 mL vial equipped with stir bar, was charged with **S053**[6] (170 mg, 470 µmol), K_2CO_3 (390 mg, 2.8 mmol), 4-Boc-aminomethyl-pinacol-phenylboronate (200 mg, 590 µmol) and $Pd(dppf)Cl_2$ (17 mg, 24 µmol). The vial was carefully flushed with Ar followed by the addition of dioxane (10 mL) and degassed water (4 mL) under Ar. The vial was sealed and heated to 85 °C resulting in brown solution. The brown solution was heated at 85 °C for 20 hours. The solution was cooled to room temperature and and EtOAc (50 mL) was added. The mixture was washed with H_2O (2 x 25 mL), dried over $MgSO_4$, filtered and volatiles were removed under vacuum. The crude residue was purified by silica gel chromatography (0→100% EtOAc/Heptane) to provide 151 mg (73% yield) of **S054** as a light brown foamy residue. ^1H NMR (400 MHz, $CDCl_3$) δ 8.18 (s, 1H), 7.61 (d, J = 8.6 Hz, 1H), 7.53 (s, 1H), 7.48 (d, J = 7.9 Hz, 2H), 7.38 (d, J = 7.9 Hz, 2H), 6.65 (d, J = 8.7 Hz, 1H), 4.95 (s, 1H), 4.37 (d, J = 6.1 Hz, 2H), 4.05 (s, 3H), 3.64 (q, J = 5.3 Hz, 2H), 3.09 (t, J = 5.9 Hz, 2H), 1.91 (s, 3H), 1.48 (s, 9H); MS (ESI+) calc'd for $C_{24}H_{31}N_4O_4$ $[M+H]^+$ 439.2, found 439.4.

To a solution of **S054** (100 mg, 39 µmol) in DCM (4 mL) was added TiPS (50 µL) followed by TFA (1 mL). The resulting solution was stirred at 22 °C for 1 hour, at which point HPLC analysis indicated full consumption of starting material. Volatiles were removed under reduced pressure and crude residue was dissolved in 5 mL MeOH and volatiles removed under vacuum. Crude **S055** was dried under high vacuum and used in the next step without further purification. ^1H NMR (400 MHz, MeOD) δ 8.15 (d, J = 8.9 Hz, 1H), 7.81 (d, J = 7.9 Hz, 2H), 7.65 (d, J = 8.1

Hz, 2H), 7.01 (d, J = 8.8 Hz, 1H), 4.22 (s, 2H), 4.16 (s, 3H), 3.43 (d, J = 7.5 Hz, 2H), 1.82 (s, 3H); MS (SI) Calc'd for $C_{19}H_{23}N_4O_2$ $[M+H]^+$ 339.2, found 339.5.

To a solution of **S055** (4.4 mg, 9.8 μmol) and DIPEA (7 μL, 40 μmol) in DMF (6 mL) was added **NanoBRET590-PEG4 SE** (5.5 mg, 8.2 μmol). The resulting solution was allowed to react at 22 °C for 19 hours at which point HPLC analysis indicated full consumption of the starting material. Solvent was removed under vacuum and the crude residue was purified by preparative HPLC (C18, 5 → 95% MeCN/H_2O, 0.05% TFA) to provide 6.1 mg (92% yield) of **S056** as a purple film. ^{1}H NMR (400 MHz, MeOD) δ 7.61 (d, J = 8.0 Hz, 2H), 7.48 (d, J = 8.0 Hz, 2H), 7.25 – 7.08 (m, 4H), 7.03 – 6.90 (m, 2H), 6.87 (d, J = 3.9 Hz, 1H), 6.34 (s, 1H), 6.29 (d, J = 4.1 Hz, 1H), 4.47 (d, J = 5.4 Hz, 2H), 4.15 (s, 3H), 3.76 (t, J = 6.0 Hz, 2H), 3.58 (dd, J = 11.0, 6.0 Hz, 12H), 3.51 (d, J = 5.3 Hz, 3H), 3.36 (s, 5H), 3.22 (d, J = 7.7 Hz, 2H), 3.03 (t, J = 7.5 Hz, 2H), 2.60 (t, J = 7.7 Hz, 2H), 2.51 (t, J = 5.9 Hz, 2H), 1.82 (s, 3H); HRMS (ESI+) calc'd for $C_{40}H_{52}BF_2N_8O_8$ $[M+H]^+$ 897.4277, found 897.4287.

Synthesis of Propranolol fluorescent tracer

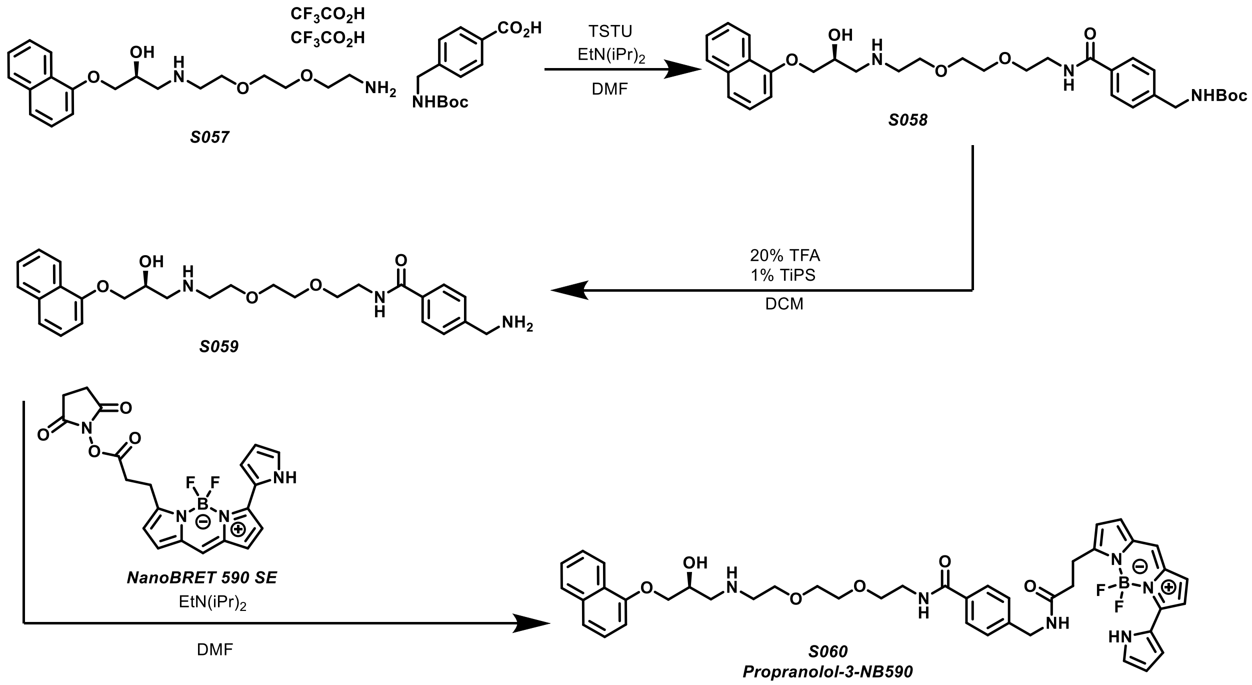

To a solution of 4-((Boc-amino)methyl)benzoic acid (7 mg, 28 μmol), DIPEA (60 μL, 0.35 mmol) in DMF (6 mL) was added TSTU (11 mg, 36 μmol) followed by **S057[1]** (30 mg, 52 μmol). The resulting solution was stirred at 22 °C for 4 hours, at which point HPLC analysis indicated full consumption of the starting material. The solvent was removed under vacuum and the crude residue was purified by preparative HPLC (C18, 5 → 95% MeCN/H_2O, 0.05% TFA) to provide 10 mg (33% yield) of **S058**. ^{1}H NMR (400 MHz, MeOD) δ 8.27 (dd, J = 7.6, 1.8 Hz, 1H), 7.81 (dd, J = 7.3, 1.9 Hz, 1H), 7.76 (d, J = 8.1 Hz, 2H), 7.54 – 7.42 (m, 3H), 7.36 (dd, J = 23.1, 7.9 Hz,

3H), 6.93 (d, J = 7.6 Hz, 1H), 4.48 – 4.36 (m, 1H), 4.34 – 4.13 (m, 4H), 3.90 – 3.74 (m, 2H), 3.71 – 3.61 (m, 6H), 3.56 (t, J = 5.7 Hz, 2H), 3.50 – 3.40 (m, 1H), 1.44 (s, 9H); MS (ESI+) calc'd for $C_{32}H_{44}N_3O_7$ $[M+H]^+$ 582.3, found 582.6.

To a solution of **S058** (10 mg, 17 μmol) in DCM (5 mL) was added TiPS (5 μL) followed by TFA (1 mL). The resulting solution was stirred at 22 °C for 1 hour, at which point HPLC analysis indicated full consumption of starting material. Volatiles were removed under reduced pressure and the crude residue was dissolved in 5 mL MeOH and volatiles removed under vacuum. The crude **S059** was dried under high vacuum and used in the next step without further purification. MS (SI) Calc'd for $C_{27}H_{36}N_3O_7$ $[M+H]^+$ 482.3, found 482.2.

To a solution of **S059** (7.2 mg, 15 μmol) and DIPEA (11 μL, 62 μmol) in DMF (6 mL) was added **NanoBRET590 SE** (2.5 mg, 5.9 μmol). The resulting solution was allowed to react at 22 °C for 2 hours at which point HPLC analysis indicated full consumption of the starting material. Solvent was removed under vacuum and the crude residue was purified by preparative HPLC (C18, 5 → 95% $MeCN/H_2O$, 0.05% TFA) to provide 8.4 mg (85% yield) of **S060** as a purple film. ^1H NMR (400 MHz, MeOD) δ 8.33 – 8.16 (m, 1H), 7.81 (dd, J = 7.5, 1.8 Hz, 1H), 7.75 (d, J = 8.1 Hz, 2H), 7.50 – 7.41 (m, 3H), 7.35 (dd, J = 23.8, 7.9 Hz, 3H), 7.25 – 7.10 (m, 4H), 7.01 (d, J = 4.6 Hz, 1H), 6.96 – 6.83 (m, 2H), 6.35 (t, J = 3.2 Hz, 1H), 6.29 (d, J = 3.9 Hz, 1H), 4.48 – 4.33 (m, 3H), 4.22 (dd, J = 9.9, 4.9 Hz, 1H), 4.15 (dd, J = 9.9, 5.6 Hz, 1H), 3.81 – 3.72 (m, 2H), 3.56 (t, J = 5.6 Hz, 2H), 3.42 (dd, J = 12.7, 3.1 Hz, 1H), 3.27 – 3.16 (m, 2H), 2.70 (t, J = 7.7 Hz, 2H); MS (ESI+) calc'd for $C_{43}H_{48}BF_2N_6O_6$ $[M+H]^+$ 793.4, found 793.6.

Synthesis of Tolterodine fluorescent tracers

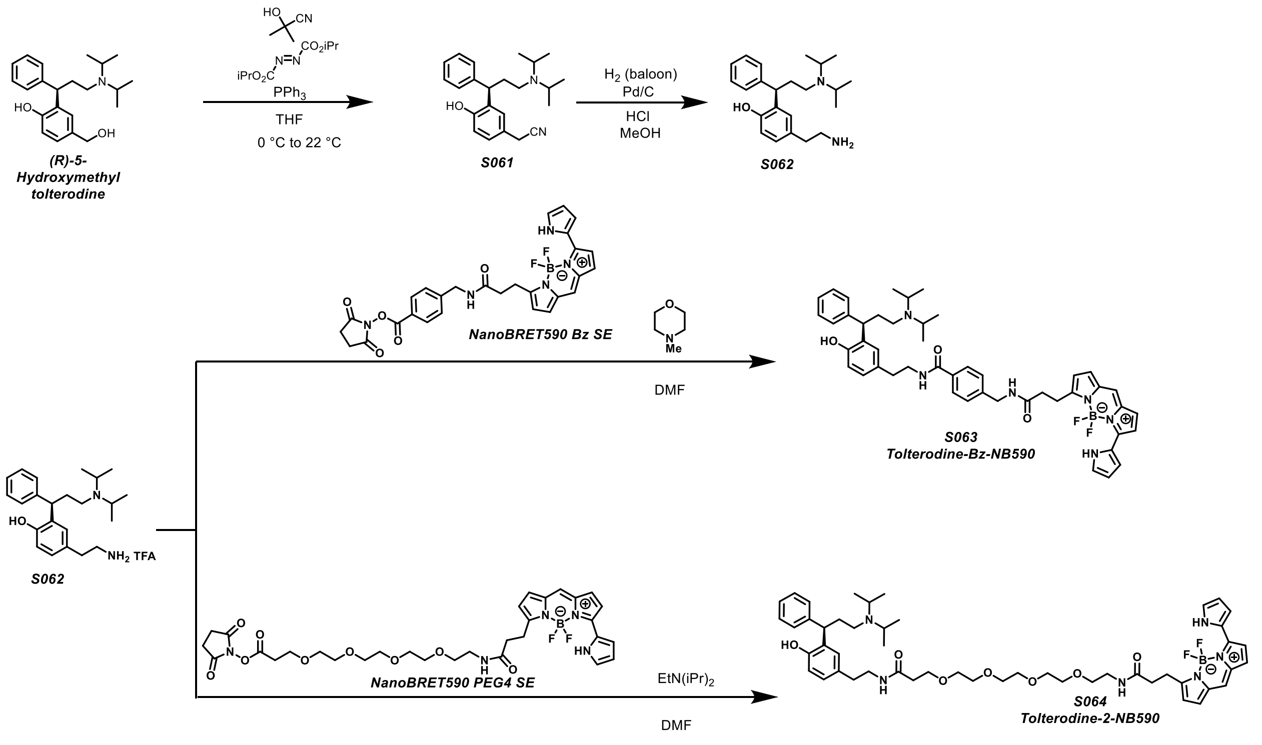

To a stirred ice-cold solution of (*R*)-5-Hydroxymethyl tolterodine (190 mg, 560 μmol) (5 mL), acetone cyanohydrine (0.10 mL, 1.1 mmol) and PPh_3 (180 mg, 700 μmol) in THF was added dropwise a solution of DEAD (135 mg, 700 μmol) in THF (5 mL) over 20 minutes. Ice bath was removed and the reaction mixture was allowed to react for 21 hours. After which time the solvent was removed under vacuum and the crude residue was purified by preparative HPLC (C18, 5 → 95% MeCN/H_2O, 0.05% TFA) to provide 41 mg (21% yield) of **S061**. ^{1}H NMR (400 MHz, MeOD) δ 9.71 (s, 1H), 8.40 (s, 1H), 7.49 – 7.27 (m, 4H), 7.21 (dt, J = 8.8, 2.5 Hz, 2H), 7.11 – 6.96 (m, 1H), 6.79 (dd, J = 16.9, 8.2 Hz, 1H), 4.36 (t, J = 7.7 Hz, 1H), 3.87 (s, 2H), 3.70 – 3.51 (m, 2H), 2.99 (d, J = 10.6 Hz, 1H), 2.84 (s, 1H), 2.38 (d, J = 8.6 Hz, 2H), 1.19 (td, J = 15.1, 6.5 Hz, 12H); MS (ESI+) calc'd for $C_{23}H_{31}N_2O$ $[M+H]^+$ 351.2, found 351.2.

A 40 mL vial, equipped with stir bar and a screw cam with septa was charged with **S061** (20 mg, 43 μmol), Pd/C (10%, 10 mg) and MeOH (3 mL). The vial was sealed and evacuated by piercing the septa with a 22G needle connected to a vacuum line. Hydrogen balloon was attached, and the vial was backfilled with hydrogen gas. Evacuation/backfill with hydrogen (3x times). Concentrated HCl (90 μL) iwas added and the solution was left stirred under H_2 baloon for two hours, after which time the solvent was removed under vacuum and the residue was purified by preparative HPLC (C18, 5 → 95% MeCN/H_2O, 0.05% TFA) to provide 5 mg (33% yield) of **S062**. ^{1}H NMR (400 MHz, DMSO-*d6*) δ 9.51 (s, 1H), 8.73 (s, 1H), 7.77 (s, 3H), 7.48 – 7.25 (m, 4H), 7.25 – 7.16 (m, 1H), 7.08 (d, J = 2.4 Hz, 1H), 7.02 – 6.87 (m, 1H), 6.76 (d, J = 8.2 Hz, 1H), 4.32 (t, J = 7.7 Hz, 1H), 2.84 (s, 1H), 2.76 – 2.68 (m, 2H), 1.19 (ddd, J = 16.9, 10.1, 6.6 Hz, 12H); MS (ESI+) calc'd for $C_{23}H_{35}N_2O$ $[M+H]^+$ 355.3, found 355.3.

To a solution of **S062** (2.0 mg, 3.6 μmol), and NMM (3 μL, 30 μmol) in DMF (5 mL) was added solution of **NanoBRET590-Bz-SE** (1.7 mg, 3.6 μmol) in DMF (1 mL). The resulting solution was allowed to react at 22 °C for 24 hours at which point HPLC analysis indicated full consumption of the starting material. Solvent was removed under vacuum and the crude residue was purified by preparative HPLC (C18, 5 → 95% MeCN/H_2O, 0.05% TFA) to provide 0.5 mg (18% yield) of **S063** as a purple film. ^{1}H NMR (400 MHz, MeOD) δ 7.77 – 7.54 (m, 2H), 7.33 (d, J = 8.1 Hz, 2H), 7.31 – 7.27 (m, 2H), 7.26 – 7.13 (m, 7H), 7.08 – 6.93 (m, 3H), 6.90 (d, J = 4.0 Hz, 1H), 6.74 (d, J = 8.1 Hz, 1H), 6.36 (dt, J = 3.8, 2.3 Hz, 1H), 6.31 (d, J = 3.9 Hz, 1H), 4.44 (d, J = 4.6 Hz, 2H), 4.35 (t, J = 7.7 Hz, 1H), 3.75 – 3.50 (m, 5H), 3.04 – 2.87 (m, 2H), 2.80 (t, J = 7.0 Hz, 2H), 2.72 (t, J = 7.7 Hz, 2H), 2.60 – 2.43 (m, 1H), 2.43 – 2.22 (m, 1H), 1.45 – 1.02 (m, 12H); HRMS (ESI+) calc'd for $C_{47}H_{54}BF_2N_6O_3$ $[M+H]^+$ 799.4313, found 799.4319.

To a solution of **S062** (1.5 mg, 3.2 μmol) and DIPEA (6 μL, 30 μmol) in DMF (6 mL) was added **NanoBRET590-PEG4 SE** (2.1 mg, 3.2 μmol). The resulting solution was allowed to react at 22 °C for 2 hours at which point HPLC analysis indicated full consumption of the starting material. Solvent was removed under vacuum and the crude residue was purified by preparative HPLC (C18, 5 → 95% MeCN/H_2O, 0.05% TFA) to provide 1.5 mg (46% yield) of **S064** as a purple film. ^{1}H NMR (400 MHz, MeOD) δ 7.36 (d, J = 7.7 Hz, 2H), 7.30 (t, J = 7.5 Hz, 2H), 7.23 (s, 1H), 7.19 (d, J = 3.3 Hz, 4H), 7.02 (d, J = 4.6 Hz, 1H), 6.99 (d, J = 2.3 Hz, 1H), 6.94 – 6.87 (m, 2H), 6.73 (d, J = 8.5 Hz, 1H), 6.34 (dd, J = 10.0, 3.6 Hz, 2H), 4.38 (t, J = 7.7 Hz, 1H), 3.78 – 3.43 (m, 18H), 3.36 (d, J = 5.3 Hz, 3H), 3.23 (d, J = 8.6 Hz, 3H), 3.02 (t, J = 8.6 Hz, 2H), 2.53 (dq, J = 16.7, 7.9 Hz, 1H), 2.47 – 2.37 (m, 1H), 2.32 (t, J = 6.0 Hz, 2H), 1.75 – 1.60 (m, 4H), 1.42 (h, J =

7.5 Hz, 4H), 1.35 – 1.16 (m, 12H); HRMS (ESI+) calc'd for $C_{50}H_{68}BF_2N_6O_7$ $[M+H]^+$ 913.5205, found 913.5220.

Synthesis of NAN190 fluorescent tracers

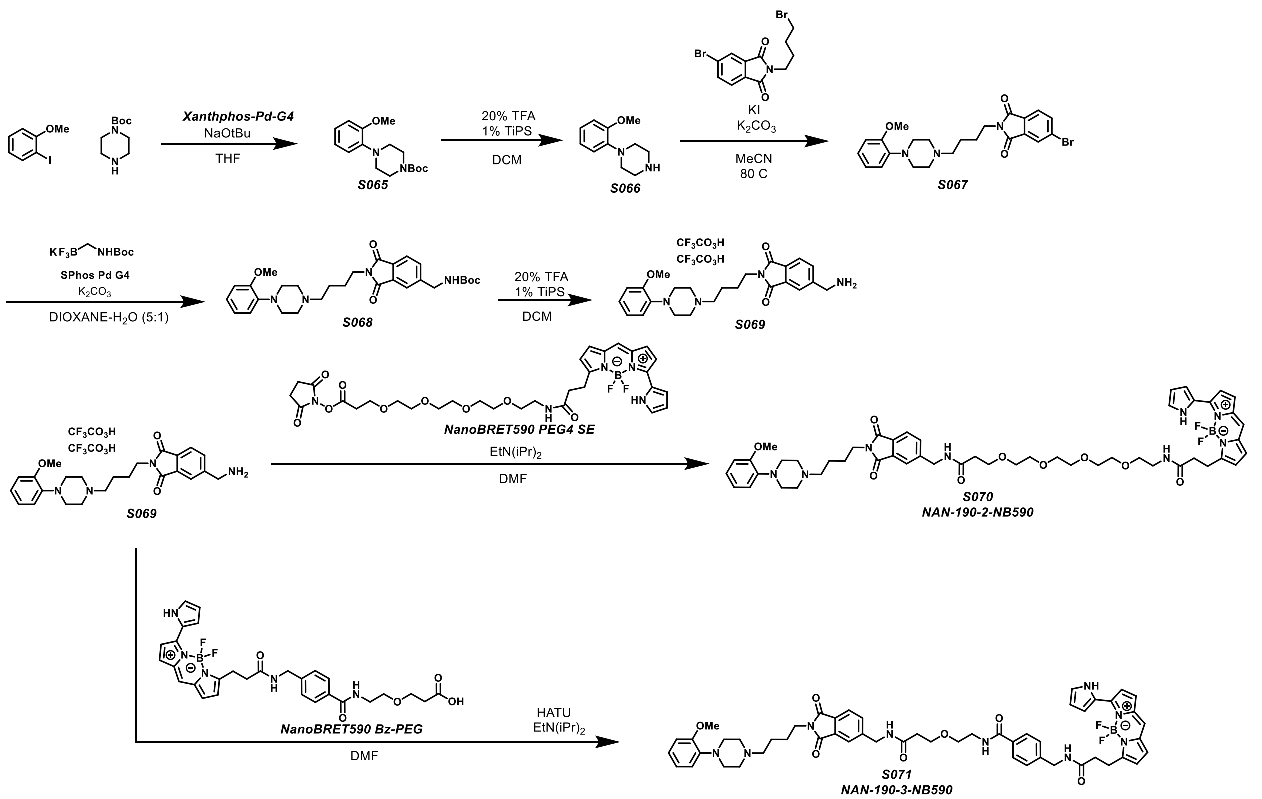

To a 20 mL vial, equipped with stir bat and a screw cap was added o-iodo-anisole (0.28 mL, 2.1 mmol), Boc-piperazine (400 mg, 2 mmol), Xanthphos-Pd-G4 (100 mg, 0.1 mmol) followed by a solution of NaOtBu in THF (1M, 7 mL, 7 mmol). The solution was purged with N_2 and heated at 60 □C for 2 hours, after which time the reaction mixture was poured into water (100 mL) and extracted into EtOAc (3 x 100 mL). The organics were combined, dried over $MgSO_4$ and the solvent was removed under vacuum. The crude residue was purified was purified by silica gel chromatography (0→10% MeOH/DCM) to provide 480 mg (77% yield) of **S065**. ^{1}H NMR (400 MHz, $CDCl_3$) δ 7.02 (dt, J = 8.8, 4.8 Hz, 1H), 6.90 (dd, J = 17.7, 6.3 Hz, 3H), 3.87 (d, J = 1.7 Hz, 3H), 3.61 (t, J = 5.1 Hz, 4H), 3.00 (t, J = 5.0 Hz, 4H), 1.49 (s, 9H); MS (SI) Calc'd for $C_{16}H_{25}N_2O_3$ $[M+H]^+$ 293.2, found 293.0.

To a solution of **S065** (480 mg, 160 μmol) in DCM (18 mL) was added TiPS (200 μL) followed by TFA (4 mL). The resulting solution was stirred at 22 °C for 1 hour, at which point HPLC analysis indicated full consumption of starting material. Volatiles were removed under reduced pressure and crude residue was dissolved in 20 mL MeOH and volatiles removed under vacuum. The crude residue was purified was purified by silica gel chromatography (0→25%

MeOH/[DCM(0.5% DIPEA)] to provide 300 mg (94% yield) of **S066**. ^{1}H NMR (400 MHz, $CDCl_3$) δ 1H NMR 7.14 – 6.97 (m, 1H), 6.97 – 6.89 (m, 2H), 6.86 (d, J = 7.9 Hz, 1H), 3.87 (s, 3H), 3.17 – 2.91 (m, 8H); MS (SI) Calc'd for $C_{11}H_{17}N_2O$ $[M+H]^+$ 193.1, found 193.2.

To a mixture of **S066** (300 mg, 1.50 mmol), 5-bromo-2-(4-bromobutyl)isoindoline-1,3-dione[7] (560 mg, 1.50 mmol), K_2CO_3 (530 mg, 1.5 mmol) and KI (26 mg, 150 μmol) in MeCN (15 mL) was heated at 80 °C for 21 hours, at which point HPLC analysis indicated full consumption of starting material. The reaction mixture was filtered through syringe filter and the volatiles were removed under reduced pressure. The crude residue was purified was purified by silica gel chromatography (0→10% MeOH/DCM) to provide 570 mg (78% yield) of **S067**. ^{1}H NMR (400 MHz, $CDCl_3$) δ 7.97 (s, 1H), 7.85 (d, J = 8.1 Hz, 1H), 7.70 (d, J = 7.7 Hz, 1H), 6.92 (td, J = 27.3, 7.6 Hz, 4H), 3.86 (s, 3H), 3.71 (q, J = 5.8, 4.7 Hz, 2H), 3.11 (s, 4H), 2.68 (s, 4H), 2.48 (t, J = 7.6 Hz, 2H), 1.73 (p, J = 7.2 Hz, 3H), 1.60 (t, J = 7.7 Hz, 3H); MS (SI) Calc'd for $C_{23}H_{27}BrN_3O_3$ $[M+H]^+$ 472.1, found 472.4.

A 75 mL pressure flask equipped with stir bar was charged with **S067**(570 mg, 1.2 mmol), K_2CO_3 (500 mg, 3.6 mmol), potassium (N-Boc-aminomethyl)trifluoroborate (350 mg, 1.5 mmol) and SPhos-Pd-G4 (48 mg, 61 μmol). The headspace was flushed with argon followed by addition of dioxane (25 mL) and degassed water (5 mL). The resulting yellow solution was degassed by passing argon through for 5 minutes. The flask was sealed and heated to 110 C for 19 hours. The solvent was removed under vacuum and the residue was dissolved in 50 mL DCM. The insoluble were filtered out and the solvent was removed under vacuum. The crude residue was purified was purified by silica gel chromatography (0→20% MeOH/DCM) to provide 70 mg (11% yield) of **S068**. ^{1}H NMR (400 MHz, $CDCl_3$) δ 1H NMR (400 MHz, Methanol-d4) δ 7.80 (d, J = 7.7 Hz, 1H), 7.76 (s, 1H), 7.70 (d, J = 7.7 Hz, 1H), 6.95 (tq, J = 23.0, 7.7 Hz, 4H), 4.37 (s, 2H), 3.85 (d, J = 1.4 Hz, 3H), 3.71 (t, J = 6.9 Hz, 2H), 3.05 (s, 4H), 2.65 (s, 4H), 2.47 (t, J = 7.8 Hz, 2H), 1.72 (p, J = 7.1 Hz, 2H), 1.59 (p, J = 7.9, 7.5 Hz, 2H), 1.46 (s, 9H); MS (SI) Calc'd for $C_{29}H_{39}N_4O_5$ $[M+H]^+$ 523.3, found 523.2.

To a solution of **S068** (70 mg, 130 μmol) in DCM (4 mL) was added TiPS (50 μL) followed by TFA (1 mL). The resulting solution was stirred at 22 °C for 1 hour, at which point HPLC analysis indicated full consumption of starting material. Volatiles were removed under reduced pressure and crude residue was dissolved in 5 mL MeOH and volatiles removed under vacuum. Crude **S069** was dried under high vacuum and used in the next step without further purification.

To a solution of **S069** (7.0 mg, 10 μmol) and DIPEA (15 μL, 80 μmol) in DMF (6 mL) was added **NanoBRET590-PEG4 SE** (5.5 mg, 8.2 μmol). The resulting solution was allowed to react at 22 °C for 2 hours at which point HPLC analysis indicated full consumption of the starting material. Solvent was removed under vacuum and the crude residue was purified by preparative HPLC (C18, 5 → 95% MeCN/H_2O, 0.05% TFA) to provide 4.9 mg (60% yield) of **S070** as a purple film. ^{1}H NMR (400 MHz, MeOD) δ 7.78 (d, J = 9.0 Hz, 2H), 7.70 (d, J = 7.7 Hz, 1H), 7.29 – 7.12 (m, 4H), 7.12 – 6.84 (m, 6H), 6.35 (dq, J = 3.9, 1.9 Hz, 1H), 6.30 (d, J = 4.0 Hz, 1H), 4.52 (d, J = 4.7 Hz, 2H), 3.85 (s, 3H), 3.72 (dt, J = 13.8, 5.8 Hz, 4H), 3.65 – 3.45 (m, 18H), 3.36 (t, J = 5.3 Hz, 2H), 3.21 (h, J = 7.4, 6.5 Hz, 8H), 2.95 (t, J = 12.4 Hz, 2H), 2.62 (t, J = 7.7 Hz, 2H), 2.50 (t, J = 5.8 Hz, 2H), 1.75 (dd, J = 8.6, 4.9 Hz, 4H); HRMS (ESI+) calc'd for $C_{51}H_{64}BF_2N_8O_9$ $[M+H]^+$ 981.4852, found 981.4855.

To a solution of **NanoBRET 590 Bz-PEG** (2.5 mg, 4.3 µmol), HATU (2.1 mg, 5.4 µmol) and DIPEA (8 µL, 43 µmol) in DMF (5 mL) was added solution of **S069** (3.7 mg, 4.3 µmol) in DMF (1 mL). The resulting solution was allowed to react at 22 °C for 2 hours at which point HPLC analysis indicated full consumption of the starting material. Solvent was removed under vacuum and the crude residue was purified by preparative HPLC (C18, 5 → 95% MeCN/H_2O, 0.05% TFA) to provide 3.4 mg (50% yield) of **S071** as a purple film. ^{1}H NMR (400 MHz, MeOD) δ 7.73 – 7.51 (m, 5H), 7.20 (ddt, J = 6.9, 4.5, 2.1 Hz, 6H), 7.11 – 7.03 (m, 1H), 7.03 – 6.99 (m, 1H), 6.97 (d, J = 8.2 Hz, 1H), 6.91 (h, J = 6.0 Hz, 3H), 6.34 (dq, J = 3.9, 1.9 Hz, 1H), 6.31 (d, J = 4.0 Hz, 1H), 4.48 (d, J = 5.0 Hz, 2H), 4.40 (s, 2H), 3.91 – 3.79 (m, 5H), 3.71 (t, J = 5.3 Hz, 2H), 3.68 – 3.45 (m, 8H), 3.26 – 3.08 (m, 4H), 2.94 (t, J = 12.3 Hz, 2H), 2.74 (t, J = 7.5 Hz, 2H), 2.58 (t, J = 5.9 Hz, 2H), 1.69 (q, J = 9.1, 6.7 Hz, 4H); HRMS (ESI+) calc'd for $C_{53}H_{59}BF_2N_9O_7$ $[M+H]^+$ 982.4593, found 982.4591.

Synthesis of Naltrexone fluorescent tracer

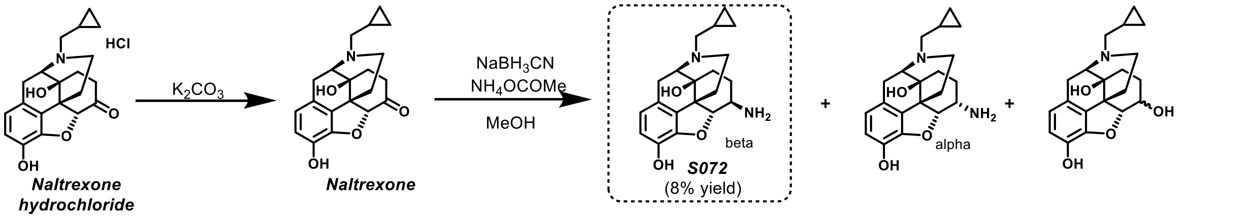

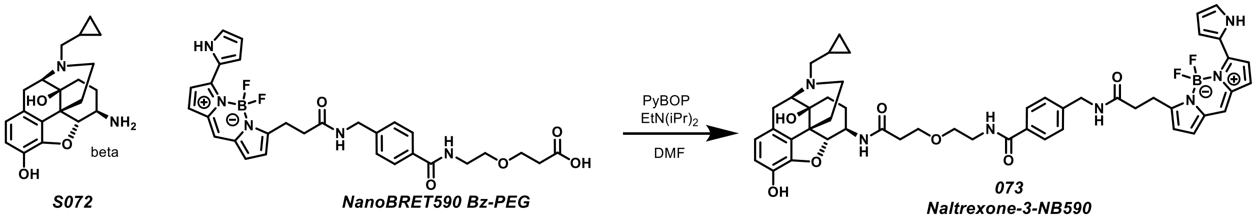

Naltrexone hydrochloride (250 mg, 660 µmol) was partitioned between DCM (10 mL) and saturated aqueous K_2CO_3 (10 mL), the resulting mixture was and stirred vigourously for 60 minutes. Organics were separated and the aqueous layer was extracted into DCM (3 x 10 ml), combined, dried $MgSO_4$, filtered and concentrated to provide 180 mg (79%) of naltrexone free base.

A solution of naltrexone (180 mg, 530 µmol) in MeOH (8 mL) was added NH_4OAc (41 mg, 530 µmol). The solution was stirred for 30 minutes after which time $NaBH_3CN$ (37 mg, 580 µmol) was added in one portion. The resulting mixture was stirred for 2 hours at 22 °C after which time H_2O (2 mL) was added to quench the reaction. Methanol was removed under vacuum and saturated aqueous K_2CO_3 (5 mL) was added to remaining residue. The aqueous mixture was

extracted into DCM (3 x 10 ml). The combined organic fractions were dried over $MgSO_4$ and solvent was removed under vacuum. The crude residue was purified by silica gel chromatography (isocratic NH_4OH-MeOH-DCM (1-6-100) to provide 15 mg (8% yield) of **S072** as a foamy white foamy residue. ^{1}H NMR (400 MHz, $CDCl_3$) δ 1H NMR (400 MHz, $CDCl_3$) δ 6.63 (dd, J = 8.1, 1.5 Hz, 1H), 6.54 (d, J = 8.0 Hz, 1H), 4.26 (d, J = 6.9 Hz, 1H, *beta isomer*), 3.49 (d, J = 1.5 Hz, 1H), 3.06 (d, J = 5.6 Hz, 1H), 2.99 (d, J = 18.2 Hz, 1H), 2.70 – 2.45 (m, 3H), 2.36 (d, J = 6.5 Hz, 2H), 2.16 (ddt, J = 29.3, 15.5, 8.3 Hz, 2H), 1.79 (dt, J = 10.9, 5.3 Hz, 2H), 1.66 – 1.53 (m, 1H), 1.41 (td, J = 12.3, 10.7, 4.9 Hz, 2H), 1.23 (d, J = 15.7 Hz, 2H), 0.84 (dh, J = 12.7, 6.0 Hz, 2H), 0.52 (d, J = 7.9 Hz, 2H), 0.12 (t, J = 5.0 Hz, 2H); MS (SI) Calc'd for $C_{20}H_{27}N_2O_3$ $[M+H]^+$ 343.2, found 343.5.

To a solution of **NanoBRET 590 Bz-PEG** (2.3 mg, 4.1 μmol), PyBOP (2.7 mg, 5.1 μmol) and DIPEA (4 μL, 22 μmol) in DMF (6 mL) was added solution of **S072** (1.4 mg, 4.1 μmol) in DMF (1 mL). The resulting solution was allowed to react at 22 °C for 16 hours at which point HPLC analysis indicated full consumption of the starting material. Solvent was removed under vacuum and the crude residue was purified by preparative HPLC (C18, 5 → 95% MeCN/H_2O, 0.05% TFA) to provide 2.3 mg (63% yield) of **S073** as a purple film. ^{1}H NMR (400 MHz, MeOD) δ 7.78 (d, J = 8.1 Hz, 2H), 7.22 (dd, J = 11.5, 6.5 Hz, 6H), 7.04 (d, J = 4.5 Hz, 1H), 6.91 (d, J = 3.9 Hz, 1H), 6.77 – 6.62 (m, 2H), 6.37 (d, J = 3.4 Hz, 1H), 6.29 (d, J = 4.0 Hz, 1H), 4.57 – 4.28 (m, 3H), 3.87 (d, J = 5.7 Hz, 1H), 3.78 (tt, J = 10.1, 5.0 Hz, 2H), 3.73 – 3.53 (m, 5H), 3.09 – 2.94 (m, 1H), 2.80 (dd, J = 13.7, 7.7 Hz, 1H), 2.72 (t, J = 7.6 Hz, 2H), 2.68 – 2.54 (m, 1H), 2.54 – 2.29 (m, 3H), 1.77 (q, J = 12.8 Hz, 1H), 1.64 – 1.19 (m, 5H), 1.03 (s, 1H), 0.77 (d, J = 7.0 Hz, 1H), 0.68 (d, J = 8.3 Hz, 1H), 0.44 (dt, J = 8.1, 4.4 Hz, 2H); HRMS (ESI+) calc'd for $C_{49}H_{53}BF_2N_7O_7$ $[M-H]^-$ 900.4073, found 900.4072.

Synthesis of XAC fluorescent tracer

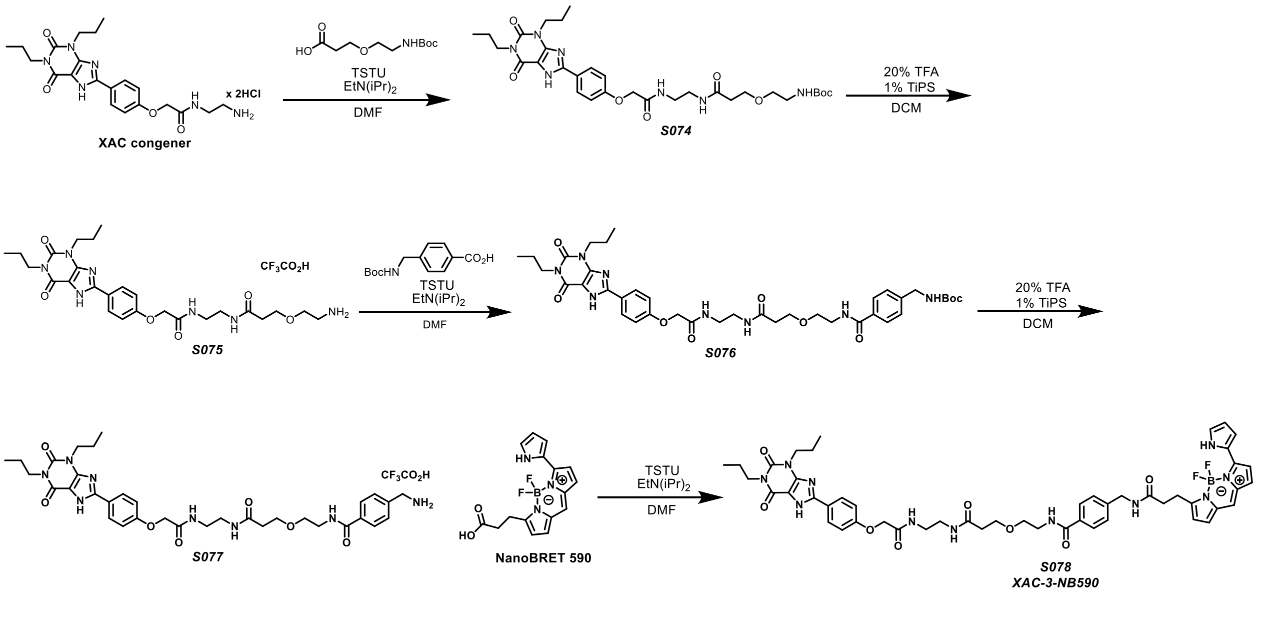

To a solution of 3-(2-((tert-butoxycarbonyl)amino)ethoxy)propanoic acid (5 mg, 20 μmol) in DMF (5 mL) was added DIPEA (34 μL, 110 μmol) and TSTU (6 mg, 20 μmol) followed by XAC congener (8.0 mg, 16 μmol). The resulting solution was stirred at 22 °C for 2 hours, at which point LCMS analysis indicated full consumption of starting material. The reaction mixture was purified by preparative RP HPLC (5→95% MeCN/H_2O buffered with 0.5% TFA) to provide 6 mg (60% yield) of **S074**. ^1H NMR (400 MHz, MeOD) δ 8.13 – 7.93 (m, 2H), 7.24 – 7.07 (m, 2H), 4.61 (s, 2H), 4.20 – 4.06 (m, 2H), 4.03 – 3.89 (m, 2H), 3.67 (t, J = 6.1 Hz, 2H), 3.55 – 3.35 (m, 6H), 3.18 (t, J = 5.6 Hz, 2H), 2.41 (t, J = 6.1 Hz, 2H), 1.92 – 1.76 (m, 2H), 1.76 – 1.59 (m, 2H), 1.41 (s, 10H), 0.98 (dt, J = 15.6, 7.5 Hz, 6H); HRMS (ESI+) calc'd for $C_{31}H_{46}N_7O_8$ $[M+H]^+$ 644.3402, found 644.3424.

To a solution of **S074** (6 mg, 9 μmol) in DCM (5 mL) was added TiPS (5 μL) followed by TFA (1 mL). The resulting solution was stirred at 22 °C for 1 hour, at which point HPLC analysis indicated full consumption of starting material. Volatiles were removed under reduced pressure and the crude residue was dissolved in 5 mL MeOH and volatiles removed under vacuum. The crude residue of **S075** was dried under high vacuum and used in the next step without further purification.

To a solution of 4-((Boc-amino)methyl)benzoic acid (3 mg, 10 μmol) in DMF (5 mL) was added DIPEA (11 μL, 64 μmol) and TSTU (4 mg, 12 μmol) followed by **S075** (6 mg, 9 μmol). The resulting solution was stirred at 22 °C for 2 hours, at which point LCMS analysis indicated full consumption of starting material. The reaction mixture was purified by preparative RP HPLC (5→95% MeCN/H_2O buffered with 0.5% TFA) to provide 4 mg (56% yield) of **S076**. ^1H NMR (400 MHz, MeOD) δ 1H NMR 8.09 – 7.85 (m, 2H), 7.76 (d, J = 8.0 Hz, 2H), 7.33 (d, J = 8.1 Hz, 2H), 7.22 – 6.91 (m, 2H), 4.58 (s, 2H), 4.25 (s, 2H), 4.20 – 4.05 (m, 2H), 4.05 – 3.89 (m, 2H), 3.71 (t, J = 6.0 Hz, 2H), 3.60 (t, J = 5.5 Hz, 2H), 3.57 – 3.48 (m, 2H), 3.45 – 3.35 (m, 3H), 2.43 (t, J = 5.9 Hz, 2H), 1.93 – 1.76 (m, 2H), 1.76 – 1.63 (m, 2H), 1.44 (s, 9H), 0.98 (dt, J = 14.7, 7.4 Hz, 6H).; HRMS (ESI+) calc'd for $C_{39}H_{53}N_8O_9$ $[M+H]^+$ 777.3930, found 777.3947.

To a solution of **S076** (4mg, 59 μmol) in DCM (5 mL) was added TiPS (5 μL) followed by TFA (1 mL). The resulting solution was stirred at 22 °C for 1 hour, at which point HPLC analysis indicated full consumption of starting material. Volatiles were removed under reduced pressure and the crude residue was dissolved in 5 mL MeOH and volatiles removed under vacuum. The crude residue of **S077** was dried under high vacuum and used in the next step without further purification.

To a solution of **S077** (1.7 mg, 2.5 μmol), TSTU (1.0 mg, 3.2 μmol) and DIPEA (3 μL, 20 μmol) in DMF (6 mL) was added **NanoBRET 590** (1.0 mg, 3.2 μmol). The resulting solution was stirred at 22 °C for 18 hours, at which point LCMS analysis indicated full consumption of starting material. The reaction mixture was purified by preparative RP HPLC (5→95% MeCN/H_2O buffered with 0.5% TFA) to provide 0.8 mg (32% yield) of **S078** as a purple film. ^1H NMR (400 MHz, MeOD) δ 8.07 – 7.85 (m, 2H), 7.85 – 7.67 (m, 2H), 7.32 (d, J = 8.3 Hz, 2H), 7.26 – 7.13 (m, 4H), 7.13 – 7.04 (m, 2H), 6.98 (d, J = 4.6 Hz, 1H), 6.86 (d, J = 3.9 Hz, 1H), 6.33 (dt, J = 4.2, 2.3 Hz, 1H), 6.26 (d, J = 3.9 Hz, 1H), 4.55 (s, 2H), 4.41 (s, 2H), 4.19 – 4.03 (m, 2H), 4.03 – 3.88 (m,

2H), 3.72 (t, J = 5.9 Hz, 2H), 3.67 – 3.51 (m, 4H), 2.69 (t, J = 7.6 Hz, 2H), 2.43 (t, J = 5.9 Hz, 2H), 1.81 (h, J = 7.4 Hz, 2H), 1.75 – 1.60 (m, 2H), 0.97 (dt, J = 9.7, 7.5 Hz, 6H); HRMS (ESI+) calc'd for $C_{50}H_{57}N_{11}O_8$ $[M+H]^+$ 988.4447, found 988.4442.

Synthesis of Loxapine fluorescent tracer

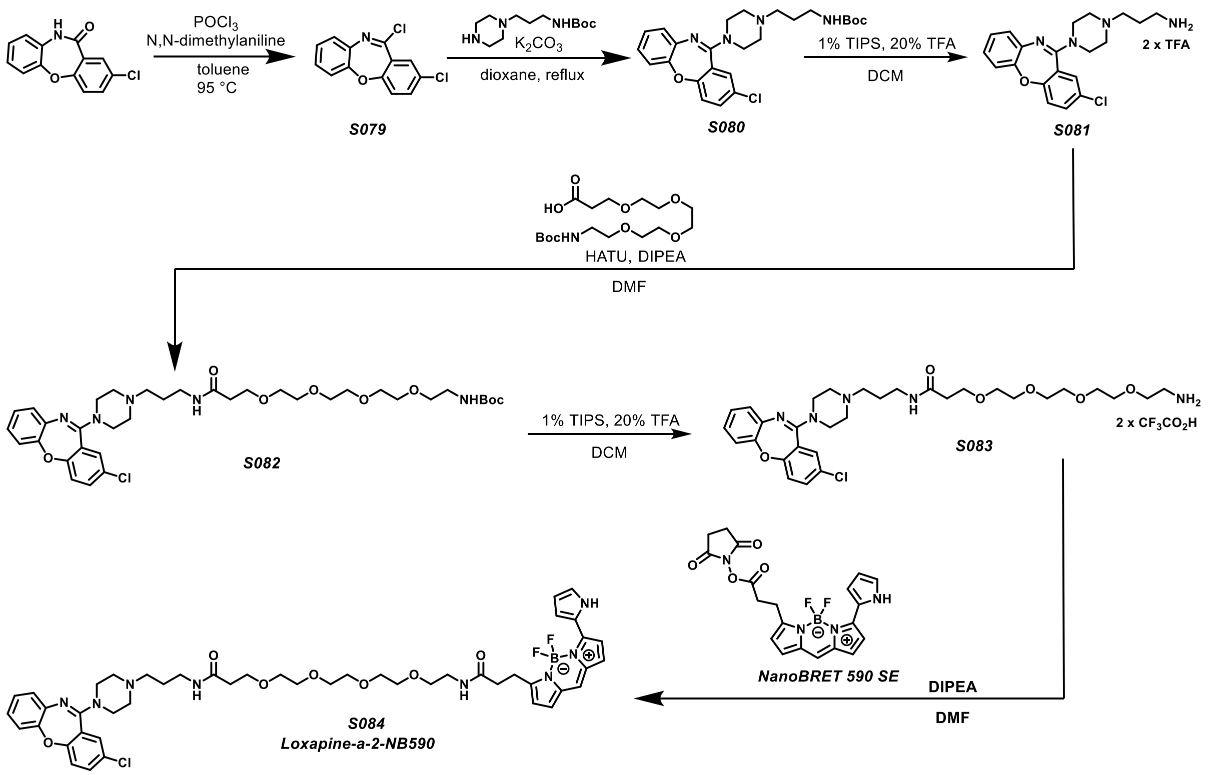

A 25 mL flask, equipped with stir bar, was charged with 2-chlorodibenzo[b,f][1,4]oxazepin-11(10H)-one (100 mg, 0.4 mmol), *N*,*N*-dimethylaniline (0.21 mL, 1.6 mmol), $POCl_3$ (114 µL, 1.14 mmol), and toluene (4 mL). The resulting suspension was heated to 95°C for 2.5 hours, and a dark brown solution formed. Solvent was removed under reduced pressure, and the residue dissolved in in a mixture of dioxane (2 mL) and aqueous 2M Na_2CO_3 (3 mL). The resulting solution was heated at 80°C for 50 minutes, dioxane removed under reduced pressure, and the residue extracted in EtOAc (3 x 10 mL). Combined EtOAc solutions were dried over $MgSO_4$, filtered, and the solvent removed under reduced pressure. The residue was purified by silica gel chromatography (0 → 30% EtOAc/hexanes) to provide 35 mg (33% yield) of imidoyl chloride **S079** as a white solid. ^{1}H NMR (400 MHz, $CDCl_3$) δ 7.71 (d, J = 2.6 Hz, 1H), 7.47 (dd, J = 8.7, 2.5 Hz, 1H), 7.33 (dd, J = 7.5, 2.1 Hz, 1H), 7.26 (td, J = 7.5, 1.7 Hz, 1H, overlap with CHCl3), 7.21 (td, J = 7.5, 1.7 Hz, 1H), 7.15 (dd, J = 7.6, 1.7 Hz, 1H), 7.13 (d, J = 8.7 Hz, 1H); MS (ESI) calc'd for $C_{13}H_8Cl_2NO$ $[M+H]^+$ 264.00 found 263.87.

A 10 mL microwave vial, equipped with stir bar, was charged with **S079** (35 mg, 0.13 mmol), tert-butyl(3-(piperazin-1-yl)propyl)carbamate (65 mg, 0.27 mmol), K_2CO_3 (46 mg, 0.33

mmol), and dioxane (3 mL). The vial was placed into a microwave reactor and heated to 120°C for 7 hours. HPLC analysis confirmed consumption of the starting material, and the solution filtered and concentrated under reduced pressure. The crude residue was purified by flash chromatography (gradient elution, 0→20% MeOH/DCM, yielding 32 mg (51% yield) of amidine **S080** as a yellow solid. ^{1}H NMR (400 MHz, MeOD) δ 7.51 (dd, J = 8.7, 2.6 Hz, 1H), 7.40 (d, J = 2.6 Hz, 1H), 7.29 (d, J = 8.7 Hz, 1H), 7.17 – 7.05 (m, 3H), 7.01 (ddd, J = 7.8, 6.7, 2.4 Hz, 1H), 3.53 (br. s, 4H), 3.10 (d, J = 6.8 Hz, 2H), 2.62 (br. s, 4H), 2.54 – 2.40 (m, 2H), 1.72 (p, J = 6.9 Hz, 2H), 1.44 (s, 9H); HRMS (ESI) calc'd for $C_{25}H_{32}ClN_4O_{3NN}$ [M+H]$^+$ 471.2163 found 471.2152.

A 25 mL flask, equipped with stir bar, was charged with **S080** (32 mg, 68 µmol) and a cleavage cocktail (10 mL, 80:20:1 DCM/TFA/TIPS). The resulting light-yellow solution was stirred at 22°C for 1 hour, at which point, HPLC indicated complete consumption of the staring material, and the solvent removed under reduced pressure. The residue was dissolved in 10 mL MeOH, and the solvent removed under reduced pressure to provide 16 mg of primary amine **S081** as a yellow oil. This material was further used without additional purification. MS (ESI) calc'd for $C_{20}H_{24}ClN_4O$ [M+H]$^+$ 371.16 found 371.22.

A 25 mL flask, equipped with stir bar, was charged with **S081** (25 mg, 37 µmol), 2,2-dimethyl-4-oxo-3,8,11,14,17-pentaoxa-5-azaicosan-20-oic acid (31 mg, 84 µmol), HATU (32 mg, 84 µmol), DIPEA (66 µL, 0.47 mmol), and DMF (6 mL). The resulting light-yellow solution was stirred at 22°C for 18 hours, at which point, HPLC indicated complete consumption of the staring material, and the solvent removed under reduced pressure. The crude residue was purified by preparative HPLC (C18, 5 → 95% MeCN/H_2O, 0.05% TFA) yielding 41 mg (85% yield) of carbamate **S082** as a clear oil. ^{1}H NMR (400 MHz, DMSO-d6) δ 9.58 (br. s, 1H), 8.05 (t, J = 5.8 Hz, 1H), 7.68 (dd, J = 8.7, 2.6 Hz, 1H), 7.57 (d, J = 2.6 Hz, 1H), 7.44 (d, J = 8.7 Hz, 1H), 7.23 (dd, J = 7.8, 1.5 Hz, 1H), 7.18 – 6.90 (m, 3H), 6.75 (t, J = 5.7 Hz, 1H), 3.61 (t, J = 6.4 Hz, 2H), 3.57 – 3.44 (m, 16H), 3.36 (t, J = 6.2 Hz, 2H), 3.26 (s, 2H), 3.17 – 3.09 (m, 4H), 3.05 (q, J = 6.0 Hz, 2H), 2.35 (d, J = 6.4 Hz, 2H), 1.97 – 1.73 (m, 2H), 1.37 (s, 9H); MS (ESI) calc'd for $C_{36}H_{53}ClN_5O_8$ [M+H]$^+$ 718.36 found 718.41.

A 25 mL flask, equipped with stir bar, was charged with **S082** (41 mg, 57 µmol) and a cleavage cocktail (10 mL, 80:20:1 DCM/TFA/TIPS). The resulting light-yellow solution was stirred at 22°C for 1 hour, at which point, HPLC indicated complete consumption of the staring material, and the solvent removed under reduced pressure. The residue was dissolved in 10 mL MeOH, and the solvent removed under reduced pressure to provide 35 mg (99% yield) primary amine **S083** as a yellow oil. This material was further used without additional purification. MS (ESI) calc'd for $C_{31}H_{45}ClN_5O_6$ [M+H]$^+$ 618.31 found 618.16.

A 25 mL flask, equipped with stir bar, was charged with **S083** (35 mg, 56 µmol), BODIPY 576/589 SE (8.0 mg, 19 µmol), DIPEA (50 µL, 0.28 mmol), and DMF (8 mL). The resulting deep purple solution was stirred at 22°C for 20 hours, at which point, HPLC indicated complete consumption of the staring material, and the reaction mixture purified by preparative HPLC (C18,

5 → 95% MeCN/H_2O, 0.05% TFA) yielding 16.4 mg (94% yield) of amide **S084** as a deep purple film. ^1H NMR (400 MHz, MeOD) δ 10.74 (s, 1H), 7.56 (dd, J = 8.7, 2.6 Hz, 1H), 7.47 (d, J = 2.6 Hz, 1H), 7.32 (d, J = 8.7 Hz, 1H), 7.24 (s, 1H), 7.22 – 7.18 (m, 3H), 7.18 – 7.12 (m, 3H), 7.12 – 7.05 (m, 1H), 6.92 (d, J = 3.9 Hz, 1H), 6.42 – 6.27 (m, 2H), 4.19 (s, 2H), 3.72 (t, J = 5.8 Hz, 2H), 3.63 – 3.55 (m, 14H), 3.52 (t, J = 5.5 Hz, 2H), 3.37 (t, J = 5.5 Hz, 2H), 3.28 (d, J = 7.7 Hz, 2H), 3.17 (t, J = 7.2 Hz, 23H), 2.65 (t, J = 7.7 Hz, 2H), 2.46 (t, J = 5.8 Hz, 2H), 1.92 (q, J = 6.8 Hz, 2H); HRMS (ESI) calc'd for $C_{47}H_{57}BClF_2N_8O_7$ $[M+H]^+$ 929.4100 found 929.4094.

Synthesis of Olanzapine fluorescent tracer

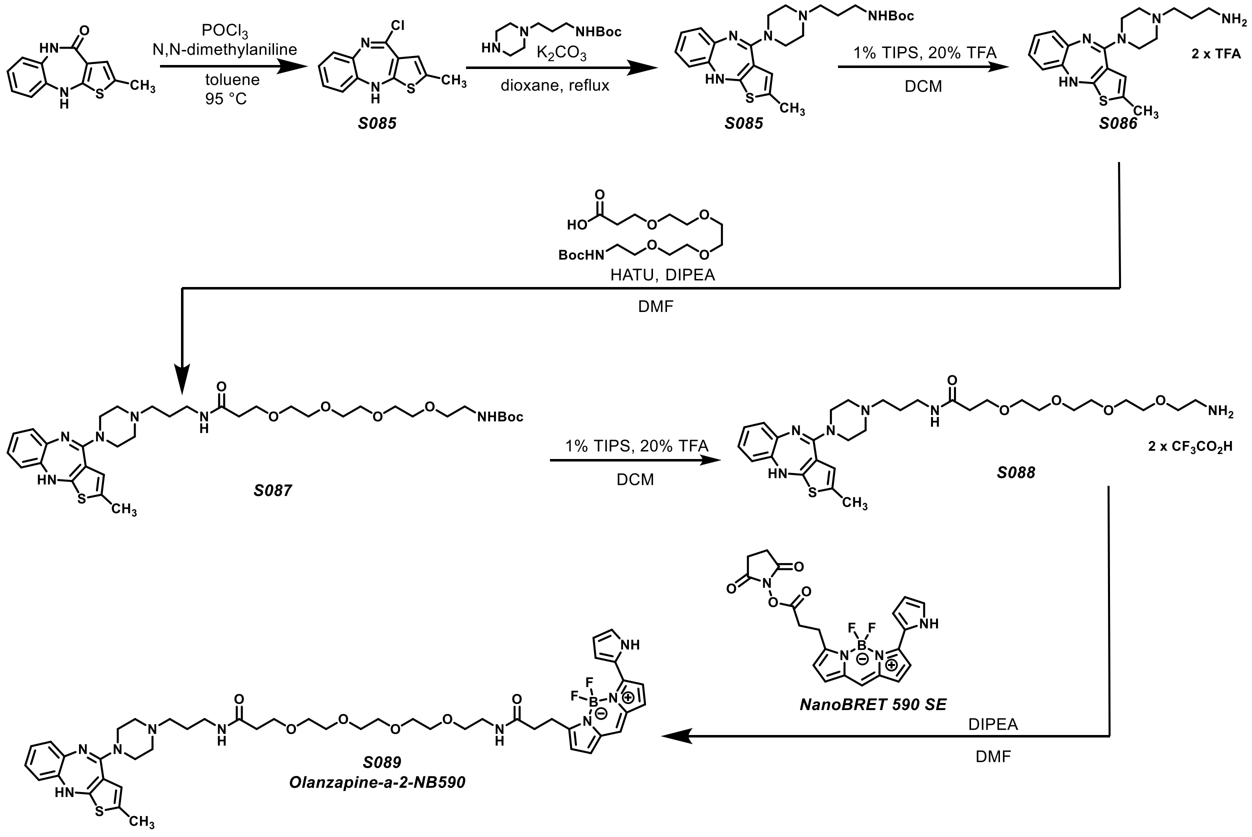

A 25 mL flask, equipped with stir bar, was charged with 2-methyl-5,10-dihydro-4H-benzo[b]thieno[2,3-e][1,4]diazepin-4-one (166 mg, 721 μmol), *N*,*N*-dimethylaniline (0.37 mL, 2.9 mmol), $POCl_3$ (200 μL, 2 mmol), and toluene (8 mL). The resulting suspension was heated to 95°C for 2.5 hours, and a dark brown solution formed. The solvent was removed under reduced pressure, and the residue dissolved in a mixture of dioxane (4 mL) and aqueous 2M Na_2CO_3 (6 mL). The resulting solution was heated at 80°C for 50 minutes, dioxane removed under reduced pressure, and the residue extracted in EtOAc (3 x 25 mL). Combined EtOAc solutions were dried over $MgSO_4$, filtered, and the solvent removed under reduced pressure. The residue was purified by

silica gel chromatography (0→20% EtOAc/hexanes) to provide 7 mg (4% yield) of imidoyl chloride **S085** as solid. ^{1}H NMR (400 MHz, DMSO-d_6) δ 8.22 (s, 1H), 7.03 (td, J = 7.6, 1.6 Hz, 1H), 6.92 (td, J = 7.6, 1.6 Hz, 1H), 6.80 (dd, J = 7.8, 1.5 Hz, 1H), 6.57 (dd, J = 7.8, 1.5 Hz, 1H), 6.44 (d, J = 1.5 Hz, 1H), 2.21 (d, J = 1.3 Hz, 3H).; MS (ESI) calc'd for $C_{12}H_{10}ClN_2S$ [M+H]$^+$ 249.03 found 249.02.

A 10 mL microwave vial, equipped with stir bar, was charged with **S085** (7.0 mg, 28 μmol), tert-butyl (3-(piperazin-1-yl)propyl)carbamate (14 mg, 56 μmol), K_2CO_3 (10 mg, 70 μmol), and dioxane (2 mL). The vial was placed into a microwave reactor and heated to 120 °C for 2 hours. HPLC analysis confirmed consumption of the starting material, and the solution was filtered and concentrated under reduced pressure. The crude residue was purified by preparative HPLC (C18, 5 → 95% MeCN/H_2O, 0.05% TFA), yielding 5.5 mg (43% yield) of carbamate **S086** as a yellow oily solid. ^{1}H NMR (400 MHz, MeOD) δ 7.29 (td, J = 7.6, 1.6 Hz, 1H), 7.25 (dd, J = 8.0, 1.6 Hz, 1H), 7.21 – 7.11 (m, 1H), 6.92 (dd, J = 8.0, 1.3 Hz, 1H), 6.62 (q, J = 1.3 Hz, 1H), 4.08 (br. s, 4H), 3.74 – 3.50 (r. s, 4H), 3.29 – 3.22 (m, 2H), 3.19 (t, J = 6.6 Hz, 2H), 2.38 (d, J = 1.3 Hz, 3H), 2.07 – 1.89 (m, 2H), 1.45 (s, 9H); HRMS (ESI) calc'd for $C_{24}H_{34}N_5O_2S$ [M+H]$^+$ 456.2433 found 456.2427.

A 25 mL flask, equipped with stir bar, was charged with **S086** (5.5 mg, 12 μmol) and a cleavage cocktail (7 mL, 80:20:1 DCM/TFA/TIPS). The resulting light-yellow solution was stirred at 22°C for 1 hour, at which point, HPLC indicated complete consumption of the staring material, and the solvent removed under reduced pressure. The residue was dissolved in 10 mL MeOH, and the solvent removed under reduced pressure to provide 4.2 mg (98% yield) of primary amine **S087** as a yellow oil. This material was further used without additional purification. MS (ESI) calc'd for $C_{19}H_{26}N_5S$ [M+H]$^+$ 356.19 found 356.08.

A 25 mL flask, equipped with stir bar, was charged with **S087** (4.2 mg, 12 mmol), 2,2-dimethyl-4-oxo-3,8,11,14,17-pentaoxa-5-azaicosan-20-oic acid (5.4 mg, 15 μmol), HATU (5.6 mg, 15 μmol), DIPEA (16 μL, 11 μmmol), and DMF (6 mL). The resulting light-yellow solution was stirred at 22°C for 3 hours, at which point, HPLC indicated complete consumption of the staring material, and the solvent removed under reduced pressure. The crude residue was purified by preparative HPLC (C18, 5 → 95% MeCN/H_2O, 0.05% TFA) yielding 9 mg (quantitative) of carbamate **S088** as a yellow oil. ^{1}H NMR (400 MHz, MeOD) δ 7.39 – 7.27 (m, 1H), 7.25 (dd, J = 8.0, 1.5 Hz, 1H), 7.22 – 7.07 (m, 1H), 6.93 (dd, J = 8.0, 1.3 Hz, 1H), 6.63 (q, J = 1.2 Hz, 1H), 4.10 (br. s, 4H), 3.77 (t, J = 5.9 Hz, 2H), 3.62 - 3.59 (m, 16H), 3.56 (q, J = 5.5 Hz, 4H), 3.40 (dd, J = 7.0, 5.6 Hz, 2H), 3.25 (d, J = 7.1 Hz, 2H), 2.51 (t, J = 5.8 Hz, 2H), 2.39 (d, J = 1.2 Hz, 3H), 2.09 – 1.95 (m, 2H), 1.43 (s, 9H); HRMS (ESI) calc'd for $C_{35}H_{54}N_6O_7SNa$ [M+Na]$^+$ 725.3672 found 725.3661.

A 25 mL flask, equipped with stir bar, was charged with **S088** (8 mg, 12 μmol) and a cleavage cocktail (7 mL, 80:20:1 DCM/TFA/TIPS). The resulting light-yellow solution was stirred at 22°C for 1 hour, at which point, HPLC indicated complete consumption of the staring material,

and the solvent removed under reduced pressure. The residue was dissolved in 10 mL MeOH, and the solvent removed under reduced pressure to provide 6 mg (85% yield) primary amine SL-1528 as a yellow oil. This material was further used without additional purification. MS (ESI) calc'd for $C_{30}H_{47}N_6O_5S$ $[M+H]^+$ 603.33 found 603.08.

A 25 mL flask, equipped with stir bar, was charged with **S088** (6 mg, 10 μmol), **NanoBRET590 SE** (3.5 mg, 8 μmol), DIPEA (14 μL, 82 μmol), and DMF (8 mL). The resulting deep purple solution was stirred at 22°C for 3 hours, at which point, HPLC indicated complete consumption of the staring material, and the reaction mixture purified by preparative HPLC (C18, 5 → 95% MeCN/H_2O, 0.05% TFA) yielding 2 mg (27% yield) of amide **S089** as a deep purple film. ^{1}H NMR (400 MHz, MeOD) δ 7.28 (ddd, J = 8.0, 7.2, 1.7 Hz, 1H), 7.25 (s, 1H), 7.24 – 7.18 (m, 4H), 7.15 (ddd, J = 8.0, 7.2, 1.3 Hz, 1H), 7.01 (d, J = 4.6 Hz, 1H), 6.96 – 6.85 (m, 2H), 6.55 (q, J = 1.2 Hz, 1H), 6.34 (td, J = 4.2, 1.8 Hz, 2H), 4.03 (s, 4H), 3.74 (t, J = 5.8 Hz, 2H), 3.59 (d, J = 3.7 Hz, 12H), 3.53 (t, J = 5.6 Hz, 2H), 3.46 (br. s, 4H), 3.41 – 3.34 (m, 4H), 3.27 (d, J = 7.6 Hz, 2H), 3.18 (t, J = 7.0 Hz, 2H), 2.64 (t, J = 7.6 Hz, 2H), 2.48 (t, J = 5.7 Hz, 2H), 2.34 (d, J = 1.2 Hz, 3H), 1.95 (p, J = 6.8 Hz, 2H); MS (ESI) calc'd for $C_{46}H_{59}BF_2N_9O_6S$ $[M+H]^+$ 914.44 found 914.26.

Synthesis of Quetiapine fluorescent tracer

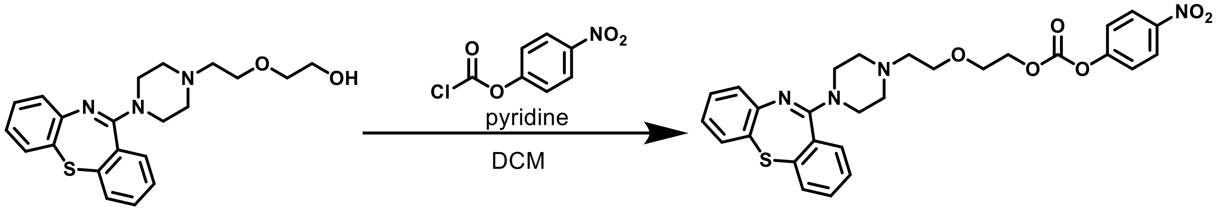

Quetiapine ***S090***

A 50 mL flask, equipped with stir bar, was charged with quetiapine (263 mg, 686 μmol), 4-nitrophenyl chloroformate (200 mg, 1 mmol), and DCM (30 mL). The resulting solution was cooled to 0ºC under Ar and pyridine (166 μL, 2.06 mmol) was added dropwise. The solution was allowed to warm up to 22°C and left stirred for 20 hours, at which point, solvent removed under reduced pressure and residue purified by silica gel chromatography (0 → 50% MeOH/DCM) to provide 121 mg (32% yield) of carbonate **S090** as a yellow oily solid. ^{1}H NMR (400 MHz, $CDCl_3$) δ 8.35 – 8.10 (m, 2H), 7.51 (dt, J = 7.3, 1.2 Hz, 1H), 7.44 – 7.35 (m, 3H), 7.35 – 7.27 (m, 3H), 7.18 (t, J = 7.7 Hz, 1H), 7.06 (dd, J = 8.0, 1.5 Hz, 1H), 6.99 – 6.78 (m, 1H), 4.52 – 4.35 (m, 2H), 3.85 – 3.75 (m, 2H), 3.70 (s, 2H), 3.37 (d, J = 151.4 Hz, 4H), 2.76 – 2.38 (m, 5H); MS (ESI) calc'd for $C_{28}H_{29}N_4O_6S$ $[M+H]^+$ 549.18 found 549.03.

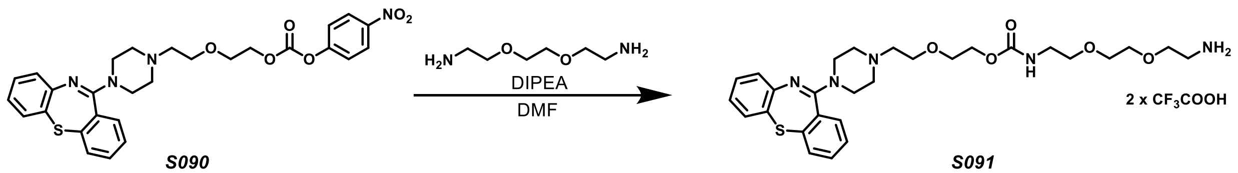

S090 *S091*

A 250 mL flask, equipped with stir bar, was charged with **S090** (60 mg, 110 µmol), 2,2'-(ethane-1,2-diylbis(oxy))bis(ethan-1-amine) (81 mg, 0.55 mmol), DIPEA (180 µL, 1.0 mmol), and DMF (100 mL). The resulting yellow solution was stirred for 18 hours at 22°C, at which point, HPLC indicated complete consumption of the staring material, and solvent removed under reduced pressure, and the residue was purified by preparative HPLC (C18, 5 → 95% MeCN/H_2O, 0.05% TFA), yielding 86 mg (quantitative) of amide **S091** as a deep purple film. ^{1}H NMR (400 MHz, MeOD) δ 7.71 (dd, J = 7.8, 1.3 Hz, 1H), 7.68 – 7.46 (m, 4H), 7.42 – 7.28 (m, 2H), 7.21 (ddd, J = 7.7, 6.8, 2.1 Hz, 1H), 4.22 (t, J = 4.6 Hz, 2H), 4.17 – 3.79 (m, 6H), 3.79 – 3.62 (m, 10H), 3.57 (d, J = 9.9 Hz, 2H), 3.52 (t, J = 5.7 Hz, 2H), 3.51 – 3.40 (m, 2H), 3.27 (t, J = 5.7 Hz, 2H), 3.12 (t, J = 5.1 Hz, 2H); HRMS (ESI) calc'd for $C_{27}H_{39}N_5O_5S$ $[M+H]^+$ 558.2750 found 558.2746.

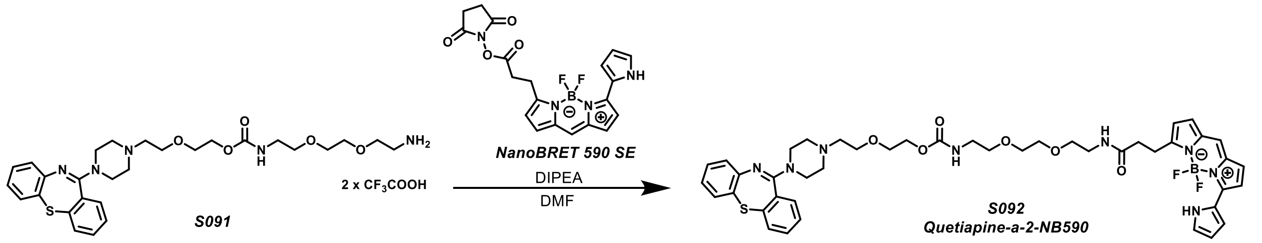

S091 *S092* *Quetiapine-a-2-NB590*

A 25 mL flask, equipped with stir bar, was charged with **S091** (8 mg, 10 µmol), **NanoBRET590 SE** (4 mg, 9 µmol), DIPEA (16 µL, 94 µmol), and DMF (8 mL). The resulting deep purple solution was stirred at 22°C for 16 hours, at which point, HPLC indicated complete consumption of the staring material, and the reaction mixture purified by preparative HPLC (C18, 5 → 95% MeCN/H_2O, 0.05% TFA) yielding 5.1 mg (63 % yield) of amide **S092** as a deep purple film. ^{1}H NMR (400 MHz, MeOD) δ δ 7.57 (dd, J = 7.7, 1.2 Hz, 1H), 7.51 – 7.35 (m, 4H), 7.28 – 7.22 (m, 2H), 7.22 – 7.16 (m, 3H), 7.09 (dd, J = 8.0, 1.4 Hz, 1H), 7.04 – 6.87 (m, 3H), 6.39 – 6.22 (m, 2H), 4.20 (t, J = 4.6 Hz, 2H), 3.83 – 3.78 (m, 2H), 3.69 (t, J = 4.5 Hz, 3H), 3.62 – 3.42 (m, 12H), 3.42 – 3.35 (m, 6H), 3.29 – 3.16 (m, 4H), 2.66 (t, J = 7.7 Hz, 2H); HRMS (ESI) calc'd for $C_{44}H_{52}BF_2N_8O_6S$ $[M+H]^+$ 869.3792 found 869.3784.

Synthesis of Risperidone fluorescent tracer

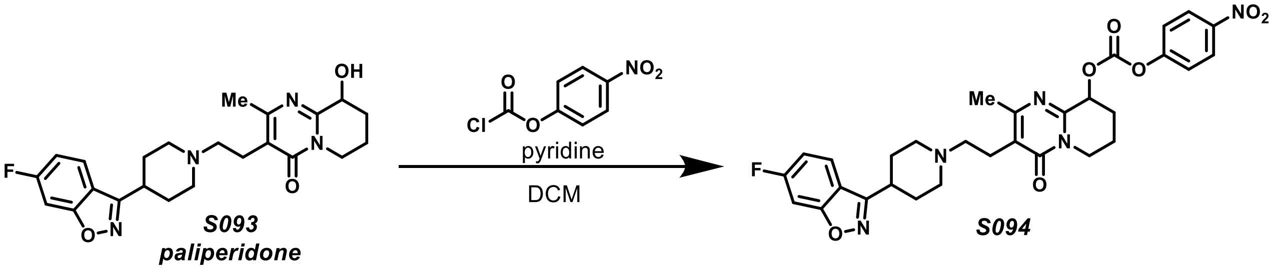

A 50 mL flask, equipped with stir bar, was charged with paliperidone (220 mg, 516 μmol), pyridine (1 mL), and DCM (10 mL). To the resulting solution, 4-nitrophenyl chloroformate (200 mg, 1 mmol) was slowly added. The solution was stirred at 22°C for 20 hours, at which point, the solution was purified by silica gel chromatography (0→50% MeOH/DCM) to provide carbonate **S094** as a yellow solid. MS (ESI) calc'd for $C_{30}H_{31}FN_5O_7$ $[M+H]^+$ 592.22 found 592.11.

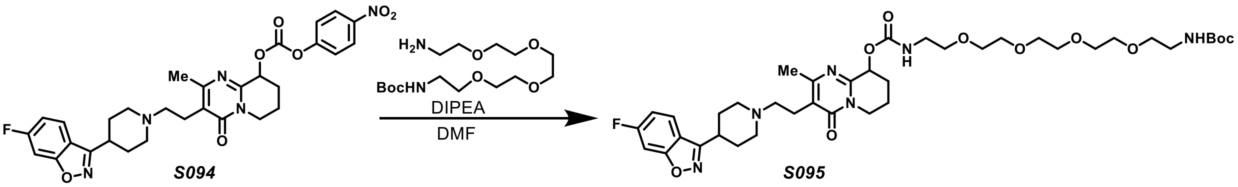

A 25 mL flask, equipped with stir bar, was charged with **S094** (19 mg, 32 μmol), tert-butyl (14-amino-3,6,9,12-tetraoxatetradecyl)carbamate (13 mg, 39 μmol), DIPEA (17 μL, 97 μmol), and MeCN (10 mL). The resulting yellow solution was stirred for 1 hour at 22°C, at which point, HPLC indicated complete consumption of the staring material, solvent removed under reduced pressure, and the residue purified by preparative HPLC (C18, 5 → 95% MeCN/H_2O, 0.05% TFA) yielding 13 mg (51% yield) of carbamate **S095** as a clear oil. ^{1}H NMR (400 MHz, MeOD) δ 8.01 – 7.75 (m, 1H), 7.45 (dd, J = 8.8, 2.2 Hz, 1H), 7.22 (td, J = 9.0, 2.2 Hz, 1H), 5.63 (t, J = 4.6 Hz, 1H), 4.17 – 4.00 (m, 1H), 4.00 – 3.79 (m, 3H), 3.79 – 3.52 (m, 16H), 3.50 (t, J = 5.7 Hz, 3H), 3.26 – 3.14 (m, 3H), 3.11 – 2.85 (m, 2H), 2.56 – 2.42 (m, 2H), 2.38 (d, J = 10.8 Hz, 3H), 2.31 – 2.15 (m, 2H), 2.15 – 1.90 (m, 4H), 1.43 (s, 9H); MS (ESI) calc'd for $C_{39}H_{58}FN_6O_{10}$ $[M+H]^+$ 789.42 found 789.29.

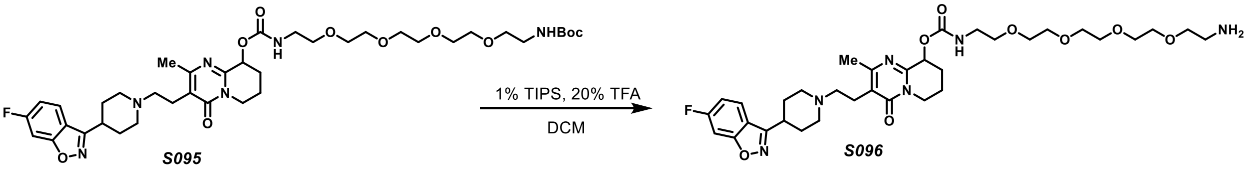

A 25 mL flask, equipped with stir bar, was charged with carbamate **S095** (13 mg, 16 μmol) and a cleavage cocktail (7 mL, 80:20:1 DCM/TFA/TIPS). The resulting light-yellow solution was stirred at 22°C for 1 hour, at which point, HPLC indicated complete consumption of the staring

material, and the solvent removed under reduced pressure. The residue was dissolved in 10 mL MeOH, and solvent removed under reduced pressure to provide 12 mg (quantitative) primary amine **S096** as a yellow oil, which was further used without additional purification. MS (ESI) calc'd for $C_{34}H_{50}FN_6O_8$ $[M+H]^+$ 689.37 found 689.34.

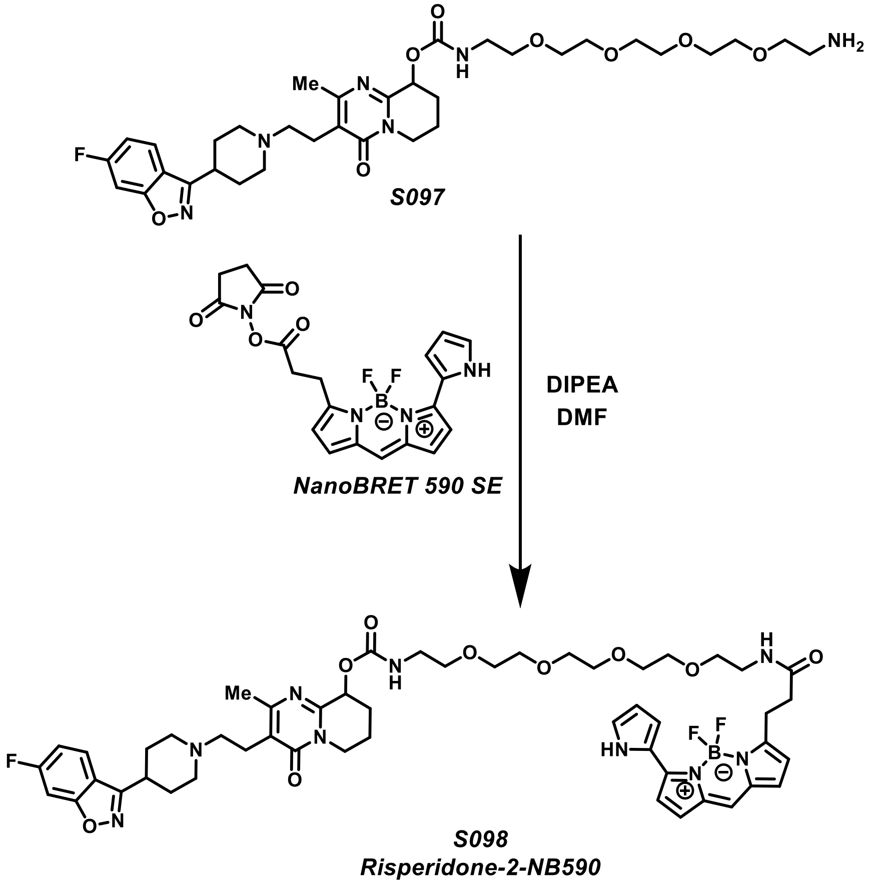

A 25 mL flask, equipped with stir bar, was charged with **S096** (12 mg, 17 µmol), **NanoBRET590 SE** (6 mg, 14 µmol), DIPEA (18 µL, 99 µmol), and DMF (7 mL). The resulting deep purple solution was stirred at 22°C for 2.5 hours, at which point, HPLC indicated complete consumption of the staring material, and the reaction mixture was purified by preparative HPLC (C18, 5 → 95% MeCN/H_2O, 0.05% TFA) yielding 3.6 mg (26 % yield) of amide **S097** as a deep purple film. ^1H NMR (400 MHz, MeOD) δ 7.86 (dd, J = 8.8, 5.0 Hz, 1H), 7.52 – 7.37 (m, 1H), 7.29 – 7.09 (m, 5H), 7.00 (d, J = 4.6 Hz, 1H), 6.92 (d, J = 4.0 Hz, 1H), 6.40 – 6.15 (m, 2H), 5.63 (d, J = 3.9 Hz, 1H), 4.05 (dd, J = 14.3, 4.8 Hz, 1H), 3.85 (t, J = 15.4 Hz, 3H), 3.53 (td, J = 5.4, 2.2 Hz, 5H), 3.37 (td, J = 5.2, 2.6 Hz, 3H), 3.28 – 3.07 (m, 6H), 2.97 (dq, J = 7.8, 3.9 Hz, 2H), 2.64 (t, J = 7.7 Hz, 2H), 2.43 (d, J = 14.9 Hz, 2H), 2.32 (d, J = 12.1 Hz, 3H), 2.24 – 1.94 (m, 5H); HRMS (ESI) calc'd for $C_{50}H_{62}BF_3N_9O_9$ $[M+H]^+$ 1001.4773 found 1000.4716.

Synthesis of Aminopotentidine fluorescent tracer

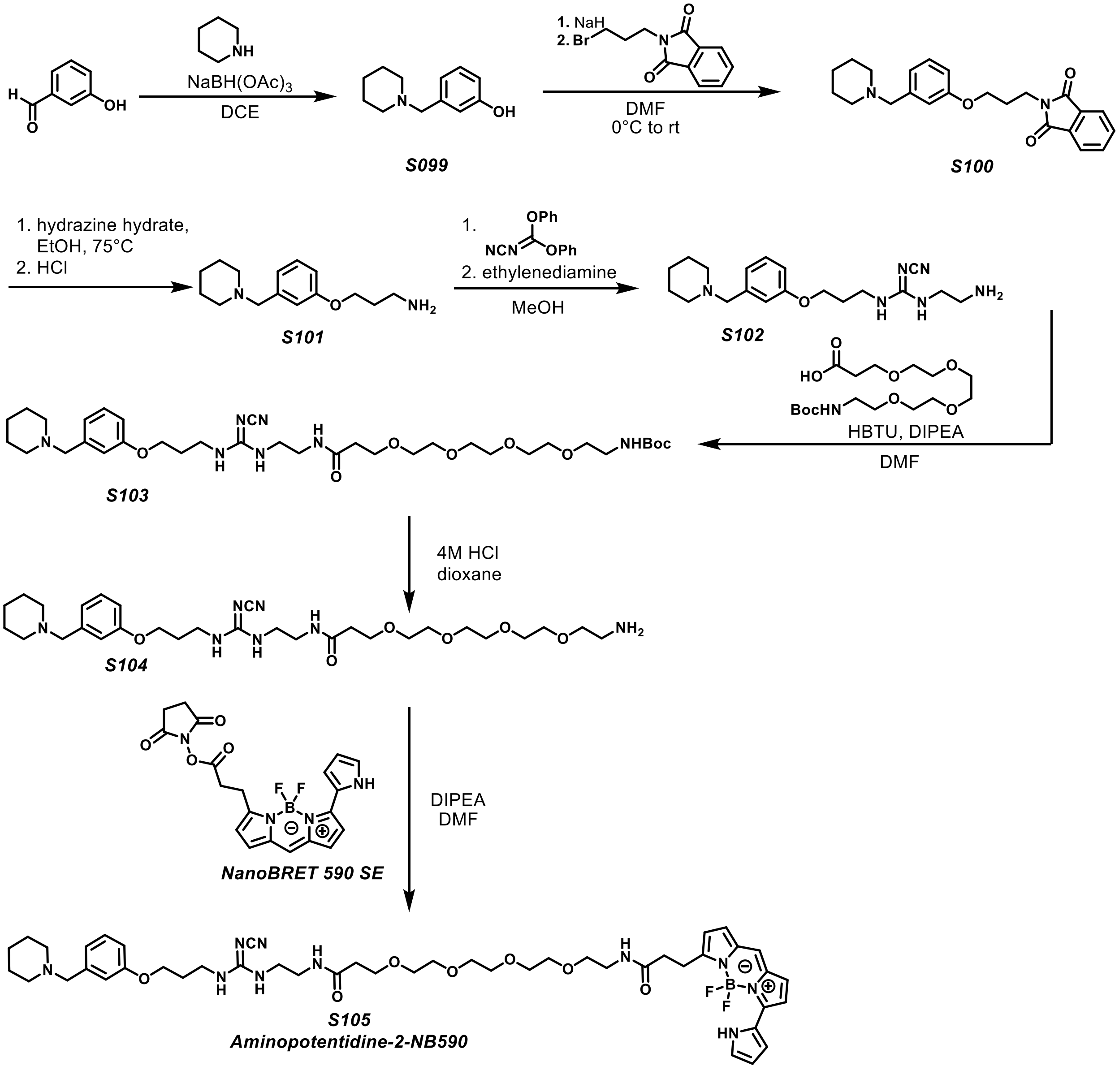

To a stirred solution of 3-hydroxybenzaldehyde (3.00 g, 24.6 mmol) and piperidine (2.30 g, 27.0 mmol) in 1,2-dichloroethane (60.0 mL) under nitrogen, was added sodium triacetoxyborohydride (7.29 g, 34.4 mmol) portion-wise. After 2 hours the reaction mixture was cooled to 0°C and methanol (20.0 mL) was added. The mixture was stirred for additional 30 minutes after which time the volatiles were removed under reduced pressure. The crude residue was partitioned between EtOAc and water. The pH of aqueous layer was adjusted to pH 7 and extracted 2 x EtOAc. The organic layers were combined, dried over $MgSO_4$ and the solvent was removed under vacuum to afford **S099** (3.32 g, 71%) as an off-white solid. MS (ESI) calc'd for $C_{12}H_{18}NO$ $[M+H]^+$ 192.1 found 191.9.

To a solution of **S099** (0.20 g, 1.1 mmol) in DMF (4.0 mL) at 0°C under nitrogen, was added sodium hydride (46.0 mg, 1.15 mmol, 60% in mineral oil). After stirring for 1hour 45 minutes *N*-(3-bromopropyl)phthalimide (0.28 g, 1.05 mmol) was added. The mixture was then allowed to warm to room temperature and was stirred for another 24 hours. Water was added, acidified to pH 4 and the mixture was extracted into Et_2O. The aqueous layer was basified to pH 10 and then extracted again with Et_2O (x 2). The organic layers were combined, dried over $MgSO_4$ and concentrated under vacuum. The crude residue was purified by silica gel chromatography 5:95 MeOH/DCM to provide 140 mg (33% yield) of **S100** as a pale-yellow oil. MS (ESI) calc'd for $C_{23}H_{27}N_2O_3$ $[M+H]^+$ 379.2 found 379.0.

To a solution of **S100** (0.684 g, 1.81 mmol) in MeOH (10 mL) was added hydrazine monohydrate (0.175 mL, 3.61 mmol) . The mixture was stirred for 4 hours at 70°C after which time another 2 equiv. of hydrazine monohydrate were added. After 22 hours the reaction mixture was cooled to room temperature and the solvent was removed under vacuum. 6N $HCl_{(aq)}$ was added and the mixture was heated at 60°C for 15 minutes. It was then cooled to room temperature and the precipitate was removed by vacuum filtration. The filtrate was basified to pH9 and extracted with a 1:1 mixture of EtOAc:Et_2O. The combined organic layers were dried over $MgSO_4$ and the solvent was removed under vacuum to afford crude **S101** 360 mg (81% yield) as a yellow oil. ^1H NMR (400 MHz, $CDCl_3$) δ 7.82 – 7.79 (m, 2H), 7.73 – 7.69 (m, 2H), 7.20 – 7.18 (m, 1H), 7.96 – 6.86 (m, 2H), 6.73 – 6.71 (m, 1H), 4.06 – 4.03 (m, 2H), 3.92 – 3.89 (m, 2H), 3.57 – 3.53 (m, 2H), 2.51 – 2.45 (m, 4H), 2.21 – 2.15 (m, 2H), 1.67 – 1.63 (m, 4H), 1.48 – 1.42 (m, 2H).

To a solution of **S101 (**200 mg, 800 umol) in MeOH (5.0 mL) was added diphenylcyanocarbonimidate (190 mg, 810 umol) was added. The mixture was stirred at room temperature for 5 hours after which time ethylene diamine (0.97 g, 16 mmol) was added. The reaction mixture was stirred at 60°C for 2 hours, after which time the solvent was removed under vacuum. The yellow residue was dissolved in $CHCl_3$ and washed with water and twice with 2M $NaOH_{(aq)}$. The organic layer was dried over $MgSO_4$ and the solvent was removed under vacuum. The yellow residue was purified by silica gel chromatography (10 → 15% [1M NH_3 in MeOH/]/DCM) to provide 233 mg (81% yield) of amine **S102** as a yellow oil. HRMS (ESI) calc'd for $C_{19}H_{31}N_6O$ $[M+H]^+$ 359.2554 found 359.2583.

To a solution of 2,2-dimethyl-4-oxo-3,8,11,14,17-pentaoxa-5-azaicosan-20-oic-acid (65 mg, 0.18 mmol) in DMF (2.5 mL) was added HBTU (74 mg, 0.20 mmol) followed by DIPEA (46 µL, 0.36 mmol). The mixture was stirred at room temperature for 5 minutes and **S102** (64 mg, 0.18 mmol) was added. The reaction mixture was stirred for 18 hours and the solvent was removed under vacuum. The crude yellow oil was purified by silica gel chromatography purified (5% 1M NH_3 in MeOH/DCM) to provide 233 mg (81% yield) of carbamate **S103** as a yellow oil. MS (ESI) calc'd for $C_{35}H_{60}N_7O_8$ $[M+H]^+$ 706.54 found 705.7.

To a solution of **S103** in MeOH (0.5 mL), 4M HCl in dioxane (0.5 mL) was added. The mixture was stirred for 2 hours and the solvent was removed under vacuum to afford crude **S104**

as a pale-yellow oil which was used directly in the next step. MS (ESI) calc'd for $C_{30}H_{52}N_7O_6$ $[M+H]^+$ 606.4 found 606.0.

To a solution of **S104** (4.4 mg, 6.5 µmol) in DMF (0.2 mL) was added DIPEA (3 µL) followed by **NanoBRET590 SE** (1.4 mg, 3.3 µmol) in DMF (1.0 mL). The mixture was stirred at room temperature with the exclusion of light for 2.5 hours. The solvent was removed under vacuum and the crude purple solid was purified by Preparative TLC (5% 1N NH_3 in MeOH/DCM) to provide 0.6 mg (20% yield) of conjugate **S105** as a purple solid. ^{1}H NMR (400 MHz, MeOD) δ 7.32 – 7.30 (m, 1H), 7.22 (s, 1H), 7.21 – 7.19 (m, 3H), 7.07 – 6.92 (m, 5H), 6.36 – 6.33 (m, 2H), 4.10 – 4.08 (m, 2H), 3.69 – 3.66 (m, 2H), 3.61 – 3.50 (m, 14H), 3.40 – 3.30 (m, 4H), 2.67 – 2.63 (m, 2H), 2.42 – 2.39 (m, 2H), 2.16 – 2.05 (m, 2H); HRMS (ESI) calc'd for $C_{46}H_{64}BF_2N_{10}O_7$ $[M+H]^+$ 917.5015 found 917.5067.

Synthesis of Pirenzepine fluorescent tracer

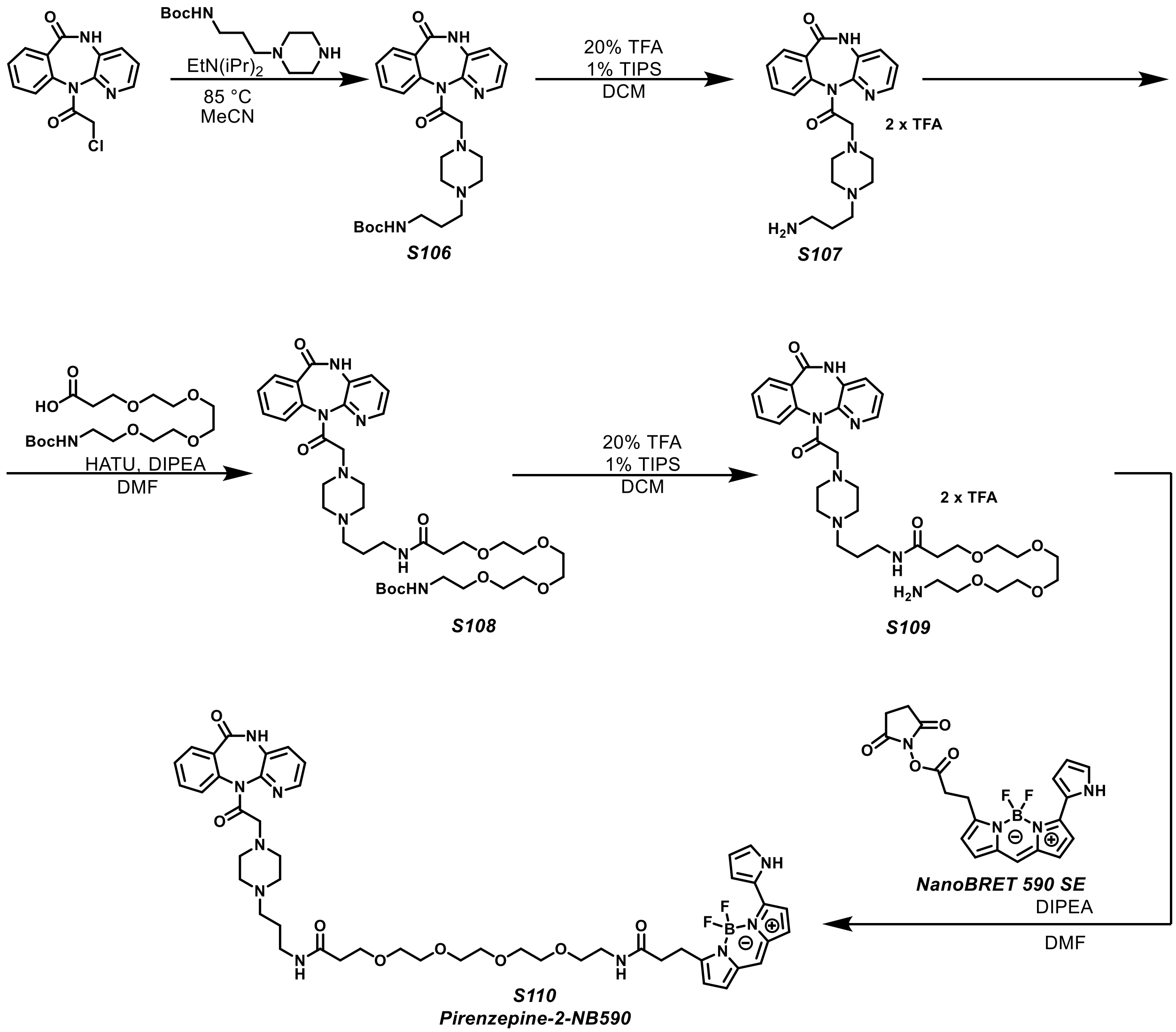

A 20 mL microwave vial, equipped with stir bar, was charged with 11-(2-chloroacetyl)-5,11-dihydro-6H-benzo[e]pyrido[3,2-b][1,4]diazepin-6-one (150 mg, 0.52 mmol), 1-(3-*N*-Boc-propyl)-piperazine (130 mg, 0.52 mmol) and MeCN (7 mL). The resulting solution was heated to 85°C for 5 hours in microwave after which time, the solvent was removed under reduced pressure, and the crude residue was purified by silica gel chromatography (0 → 100% MeOH/DCM) to provide 140 mg (54% yield) of **S106** as a yellow solid. 1H NMR (400 MHz, DMSO-d_6) δ 10.86 (s, 1H), 8.20 (s, 1H), 7.79 (d, J = 7.8 Hz, 1H), 7.65 (t, J = 8.1 Hz, 2H), 7.54 – 7.22 (m, 3H), 6.75 (s, 1H), 3.82 – 3.49 (m, 1H), 3.04 – 2.69 (m, 3H), 2.16 (s, 5H), 1.92 (d, J = 10.6 Hz, 2H), 1.45 (d, J = 6.5 Hz, 2H), 1.36 (s, 9H); MS (ESI) calc'd for $C_{26}H_{35}N_6O_4$ $[M+H]^+$ 495.3 found 495.2.

A 50 mL flask, equipped with stir bar, was charged with **S106** (140 mg, 280 μmol) and a cleavage cocktail (15 mL, 80:20:1 DCM/TFA/TIPS). The resulting light-yellow solution was stirred at 22°C for 1 hour, at which point, HPLC indicated complete consumption of the staring material, and the solvent removed under reduced pressure. The residue was dissolved in 10 mL MeOH, and solvent removed under reduced pressure to provide crude amine **S107** as a yellow oil. This material was further used without additional purification.

A 25 mL flask, equipped with stir bar, was charged with **S107** (46 mg, 63 μmol), 2,2-dimethyl-4 oxo-3,8,11,14,17-pentaoxa-5-azaicosan-20-oic acid (29 mg, 78 μmol), HATU (30 mg, 78 μmol), EtN(*i*Pr)$_2$ (110 μL, 0.62 mmol), and DMF (6 mL). The resulting light-yellow solution was stirred at 22°C for 3 hours, at which point, HPLC indicated complete consumption of the staring material, and the solvent removed under reduced pressure. The crude residue was purified by preparative HPLC (C18, 5 → 95% MeCN/H_2O, 0.05% TFA) yielding 25 mg (54% yield) of amide **S108** as a clear oil. ^{1}H NMR (400 MHz, MeOD) δ 8.34 (s, 1H), 7.90 (d, *J* = 7.9 Hz, 1H), 7.72 (d, *J* = 8.3 Hz, 2H), 7.54 (m, 2H), 7.48 (dd, *J* = 8.0, 4.8 Hz, 1H), 3.73 (t, *J* = 6.1 Hz, 5H), 3.65 – 3.55 (m, 24H), 3.50 (td, *J* = 5.7, 4.4 Hz, 6H), 3.21 (td, *J* = 5.6, 3.1 Hz, 5H), 3.15 – 2.98 (m, 4H), 2.77 – 2.59 (m, 2H), 2.55 (t, *J* = 6.3 Hz, 2H), 2.47 (t, *J* = 5.8 Hz, 3H), 1.98 – 1.80 (m, 2H), 1.43 (d, *J* = 3.5 Hz, 17H); HRMS (ESI) calc'd for $C_{37}H_{56}N_7O_9$ $[M+H]^+$ 742.4129 found 742.4134.

A 25 mL flask, equipped with stir bar, was charged with **S108** (14 mg, 19 μmol) and a cleavage cocktail (5 mL, 80:20:1 DCM/TFA/TIPS). The resulting light-yellow solution was stirred at 22°C for 1 hour, at which point, HPLC indicated complete consumption of the staring material, and the solvent removed under reduced pressure. The residue was dissolved in 10 mL MeOH, and solvent removed under reduced pressure to provide crude amine **S109** as a yellow oil. This material was further used without additional purification. MS (ESI) calc'd for $C_{32}H_{48}N_7O_7$ $[M+H]^+$ 642.4 found 642.4.

To a solution of **S109** (12 mg, 18 μmol) in DMF (8 mL) was added DIPEA (32 μL, 180 μmol) followed by **NanoBRET 590 PEG4 SE** (7.7 mg, 18 μmol). The resulting solution was allowed to react at 22ºC for 24 hours at which point HPLC analysis indicated full consumption of the starting material. Solvent was removed under vacuum, and the crude residue was purified by preparative RP HPLC (5→95% MeCN/H_2O buffered with 0.5% TFA) to provide 2.6 mg (15%

yield) of **S110** as a purple film. HPLC: 99% purity at 254 nm; ^{1}H NMR (400 MHz, MeOD) δ 10.73 (s, 1H), 8.30 (s, 1H), 7.96 – 7.88 (m, 1H), 7.75 – 7.68 (m, 2H), 7.61 – 7.48 (m, 2H), 7.45 (dd, *J* = 8.1, 4.8 Hz, 1H), 7.25 (s, 1H), 7.21 (dd, *J* = 3.6, 2.8 Hz, 3H), 7.02 (d, *J* = 4.6 Hz, 1H), 6.93 (d, *J* = 4.0 Hz, 1H), 6.48 – 6.19 (m, 2H), 3.69 (t, *J* = 5.9 Hz, 2H), 3.63 – 3.50 (m, 14H), 3.45 (s, 1H), 3.38 (t, *J* = 5.5 Hz, 3H), 3.29 – 3.20 (m, 3H), 3.03 (t, *J* = 7.1 Hz, 2H), 2.66 (dd, *J* = 8.3, 7.1 Hz, 3H), 2.43 (t, *J* = 5.8 Hz, 2H), 1.85 (p, *J* = 6.9 Hz, 2H); HRMS (SI) Calc'd $C_{48}H_{60}BF_2N_{10}O_8^+$ $[M+H]^+$ 953.4651, found 953.4661.

Synthesis of CTX-0294885 fluorescent tracer

Synthesis of CTX-0294885 fluorescent tracer was performed according to published protocol [8].

REFERENCES FOR SUPPLEMENTARY INFORMATION PART 2

5. Ottesen, L.K., F. Ek, and R. Olsson, *Iron-catalyzed cross-coupling of imidoyl chlorides with Grignard reagents.* Org Lett, 2006. **8**(9): p. 1771-3.
6. Viault, G., et al., *Design, synthesis and biological evaluation of fluorescent ligands for MT1 and/or MT2 melatonin receptors.* RSC Advances, 2016. **6**(67): p. 62508-62521.
7. Qian, L., et al., *Intracellular Delivery of Native Proteins Facilitated by Cell-Penetrating Poly(disulfide)s.* Angew Chem Int Ed Engl, 2018. **57**(6): p. 1532-1536.
8. Vasta, J.D., et al., *Quantitative, Wide-Spectrum Kinase Profiling in Live Cells for Assessing the Effect of Cellular ATP on Target Engagement.* Cell Chem Biol, 2018. **25**(2): p. 206-214 e11.
